# Cu-Catalyzed Reductive Coupling of Allylic and Carbonyl Electrophiles

**DOI:** 10.1021/acs.orglett.6c00497

**Published:** 2026-03-12

**Authors:** Vaughn Beale, Ming Chen

**Affiliations:** Department of Chemistry, 1757 Virginia Tech, Blacksburg, Virginia 24061, United States

## Abstract

We report herein Cu-catalyzed syntheses of secondary and tertiary homoallylic alcohols. Compared to conventional carbonyl addition with nucleophilic allylation reagents, this process utilizes a reductive coupling strategy in which electrophilic allylic esters couple with carbonyl compounds with B_2_pin_2_ as the reductant. Notably, the approach employs an earth-abundant metal catalyst without using cryogenic conditions.

## Graphical abstract

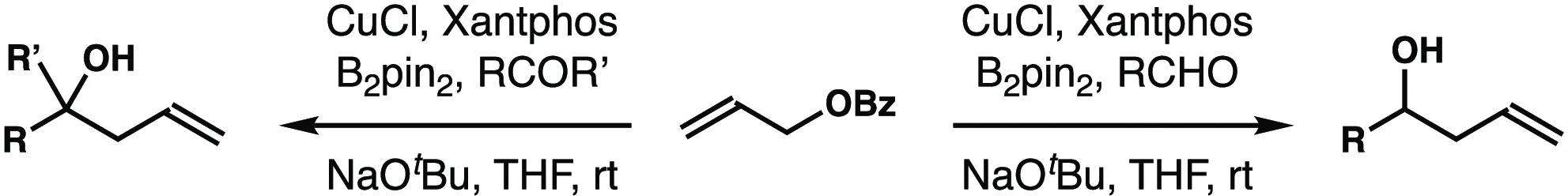

Homoallylic alcohols, including secondary and tertiary alcohols, are common structural motifs in numerous natural products and pharmaceuticals (Figure 1).^1–3^ Owing to the biological relevance and structural diversity of these molecules, significant effort has been devoted to the development of methods that could allow for the access to homoallylic alcohols.^4,5^ Traditionally, carbonyl addition with nucleophilic allylmetal reagents has been one of the most adopted approaches to synthesize homoallylic alcohols (Scheme 1a). Over the past few decades, many classes of nucleophilic allylation reagents have been developed, including those derived from main group elements or transition metals, preformed, or *in situ* generated. Notable recent achievements in this research area comprise the development of novel catalysts for asymmetric allyl addition, the scope expansion to previously nonreactive substrates, and the implementation of innovative catalytic allyl addition processes.^6^ In spite of these advances, the development of approaches that are complementary to currently available methods is valuable.

**Figure 1:**
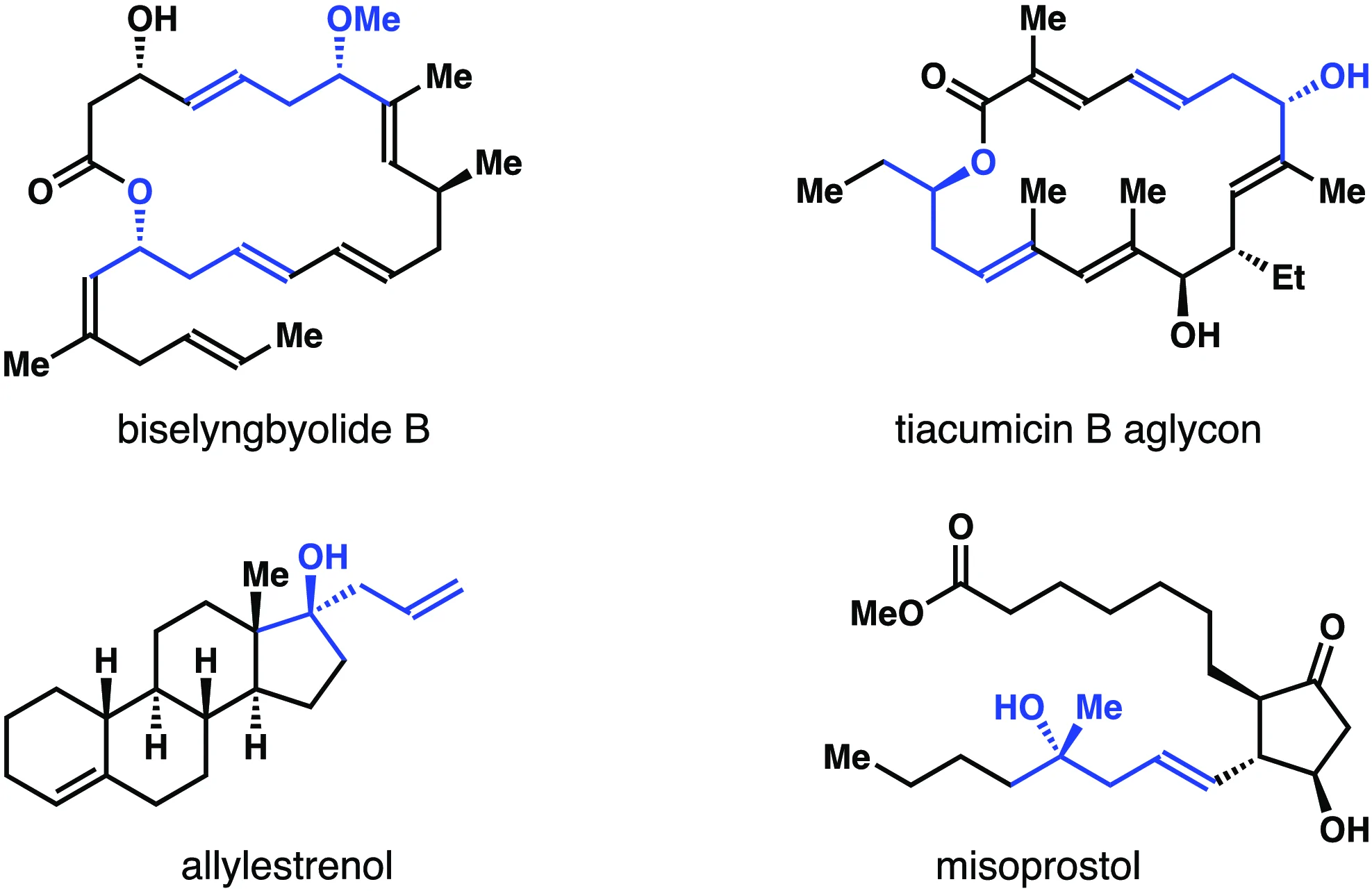
Selected molecules containing secondary or tertiary homoallylic alcohol structural motif.

**Scheme 1:**
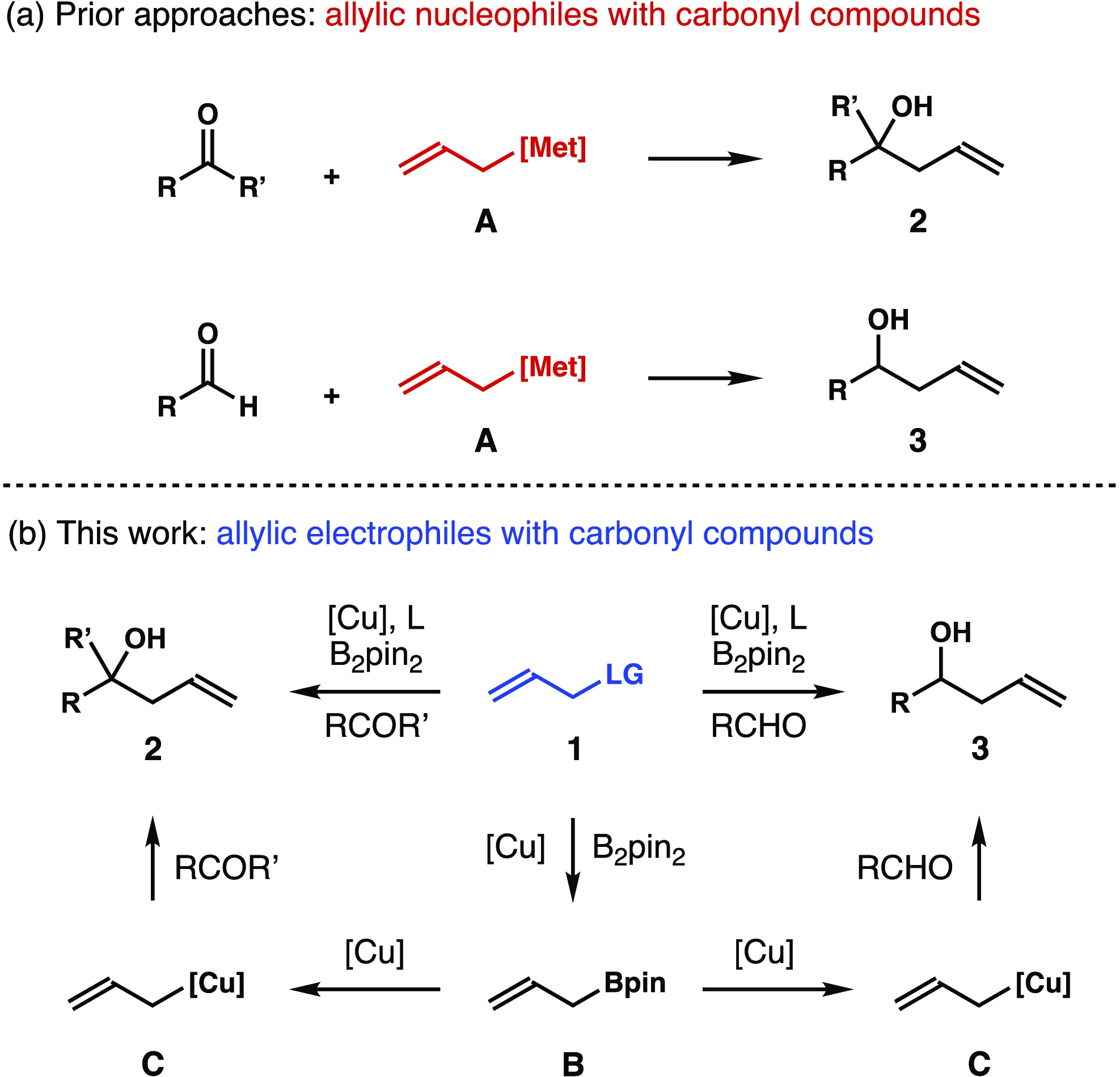
Approaches to Homoallylic Alcohols

As part of our program on allylation chemistry, we were intrigued about the possibility of coupling an allylic electrophile with carbonyl compounds to generate homoallylic alcohols under proper conditions.^7^ As illustrated in Scheme 1b, we envisioned that allylic electrophile **1** could participate in Cu-catalyzed allylic borylation to generate allylboronate intermediate **B**.^8^ Because this transformation is typically conducted under basic conditions, we suspect that allylboronate **B** could undergo base-promoted transmetalation with the copper catalyst to form allylcopper species **C** *in situ*.^9^ This reaction sequence would invert the polarity of allylic electrophile **1** to nucleophilic allylcopper **C**.^10^ Subsequent addition of **C** to ketones or aldehydes should produce homoallylic alcohols **2** or **3**. We report herein the successful realization of this proposal to synthesize homoallylic alcohols. There are a few attractive features of this approach. First, while conventional approaches utilize allylic metal reagents that are often sensitive to moisture and oxygen, the approach employs allylic esters as allyl donors that are much more stable. Second, the reaction does not require any precious metal; it utilizes a catalyst derived from an inexpensive and earth-abundant metal. Lastly, this process allows for the coupling of two electrophilic species with B_2_pin_2_ as the reductant.

While a plethora of allylation reagents can react with aldehydes to produce secondary homoallylic alcohols, methods that permit the access to tertiary homoallylic alcohols are underdeveloped.^11,12^ We started the investigation by using allylic electrophile **1** and acetophenone as the model substrates to generate tertiary homoallylic alcohols **2a**. As shown in Scheme 2, when the reaction was conducted with allylic acetate **1a** using CuCl and Xantphos as the catalytic system with KO^*t*^Bu as the base, product **2a** was isolated in 55% yield (entry 1). The reaction of allylic tosylate **1b** under the same reaction conditions gave **2a** in a slightly diminished yield (47%, entry 2). While the yield of **2a** was improved to 68% with allylic carbamate **1c**, poor results were obtained with allylic carbamate **1d** (entries 3 and 4). The reaction with allylic benzoate **1e** proceeded with a yield similar to that of **1c** (entry 5). Changing the catalyst to CuOAc or the base to LiO^*t*^Bu did not improve the yields of **2a** in the reactions with **1e** (entries 6 and 7). However, when the reaction was conducted with NaO^*t*^Bu as the base, a substantial increase of the yield was observed, and **2a** was isolated in 98% yield (entry 8).

**Scheme 2:**
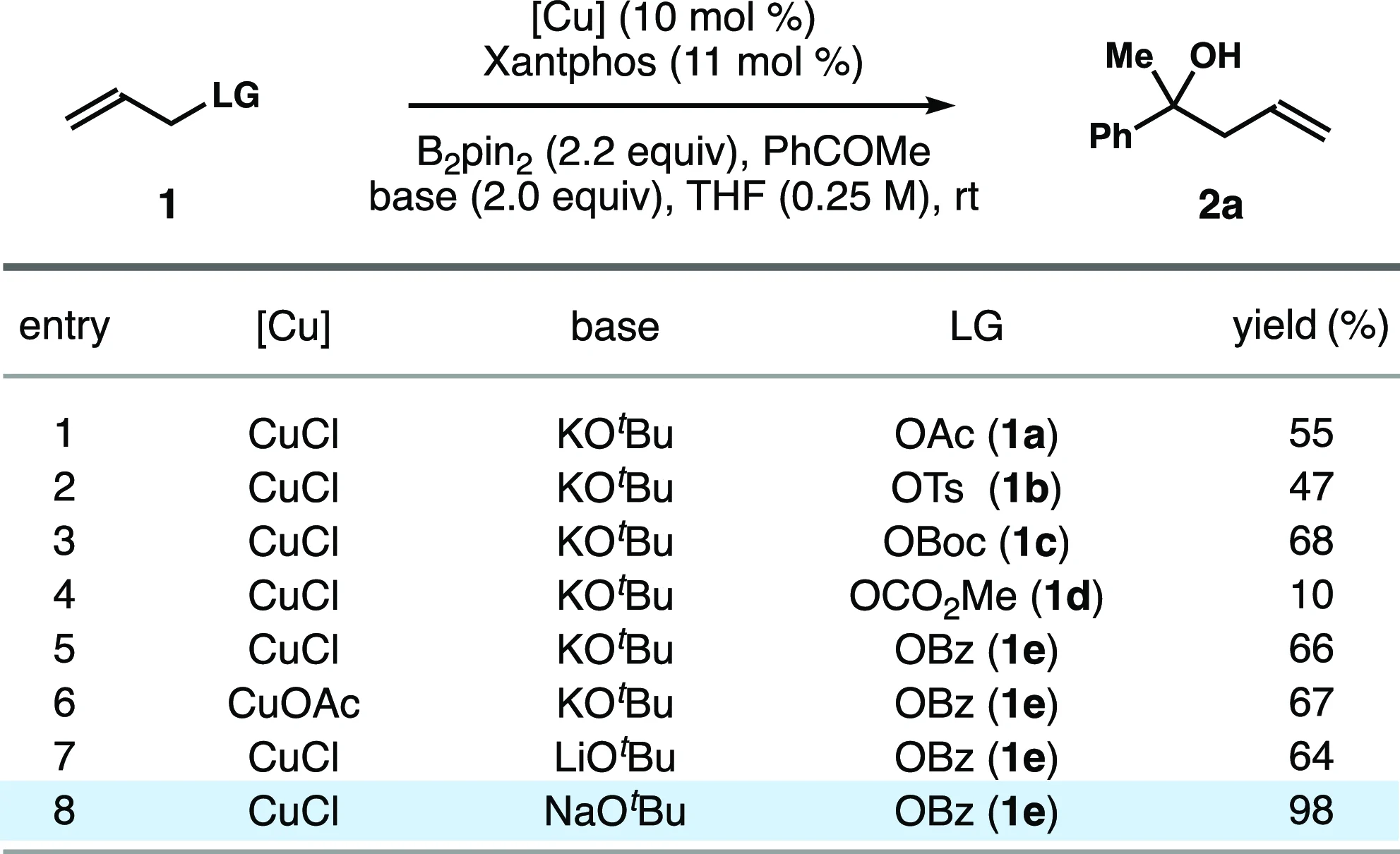
Investigation of the Reaction Conditions^a^ ^,^ ^b^

a Allylic electrophile **1** (0.3 mmol, 3 equiv), [Cu] (10 mol %), Xantphos (11 mol %), B_2_pin_2_ (2.2 equiv), base (2.0 equiv), ketone (0.1 mmol, 1.0 equiv), THF (0.25 M), rt.
b Yields of isolated products are listed.

Scheme 3 summarizes the results of the reactions of **1e** with representative ketones under the developed conditions. The reaction of an aromatic ketone with an electron-withdrawing substituent at the *para* position gave tertiary alcohol **2b** in 89% yield. Aromatic ketones with an electron-donating substituent at the *para* position also reacted under the standard conditions to deliver products **2c**-**d** in 72–79% yields. The reactions tolerate halogen-substituted aromatic ketones with different substitution patterns, affording alcohols **2e**-**h** in 75–92% yields. The ketone with a heterocyclic thiophen group is suitable for the reaction to form alcohol **2i** in 92% yield. The reaction with 1-tetralone furnished product **2j** in 66% yield. In addition to aromatic ketones, the reaction also proceeded with dialkyl ketones. For instance, 1-(4-ethylphenyl)-2-propanone reacted to give alcohol **2k** in 42% yield. The reaction with cyclohexanone generated achiral product **2l** in 50% yield. In these cases, the results are inferior to those from aromatic ketones presumably owing to their lower reactivities and potential enolization issues. The reactions using 2 equiv of **1e** gave the desired products with comparable yields.

**Scheme 3:**
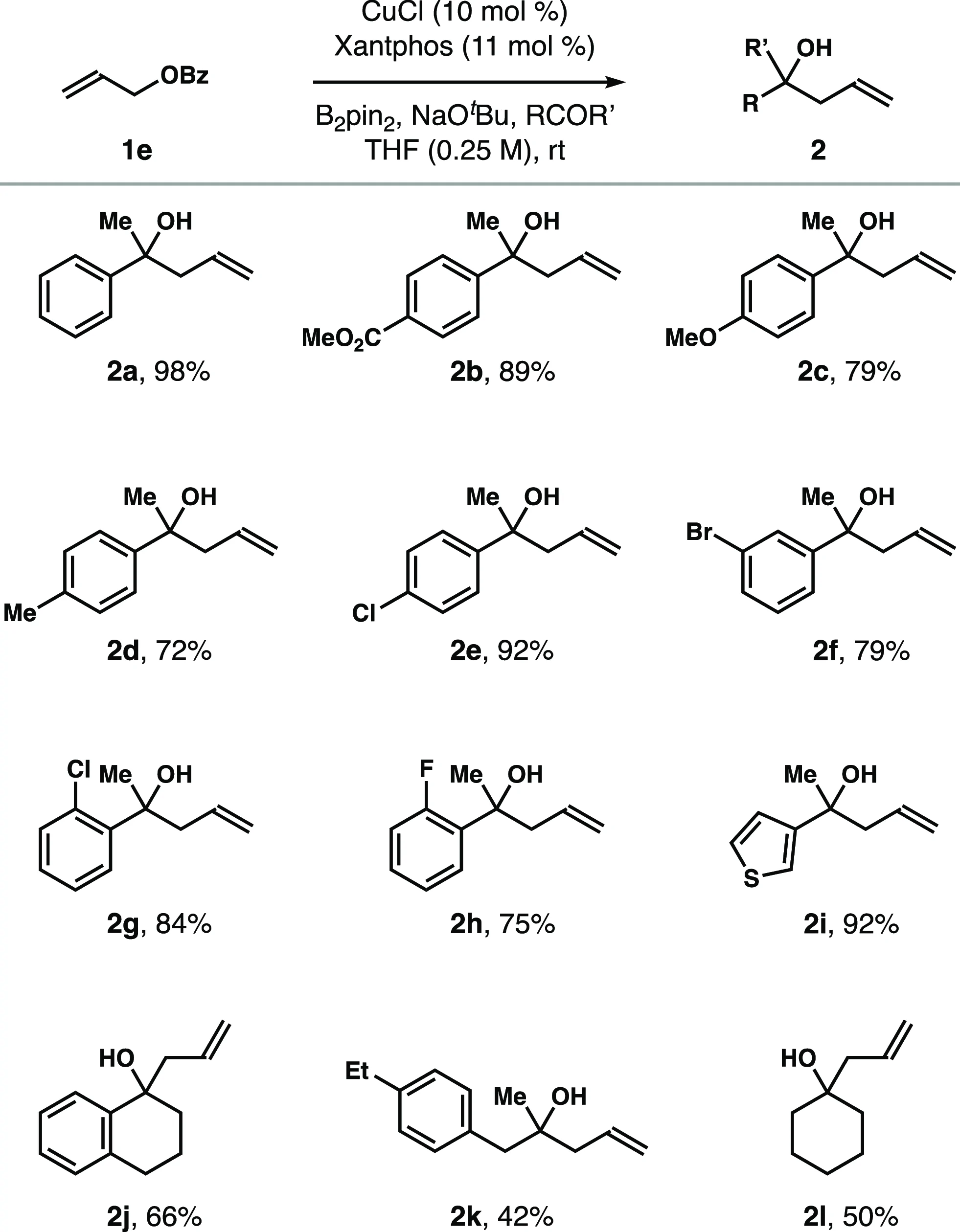
Reactions with Representative Ketones^a^ ^,^ ^b^

a Allylic benzoate **1e** (0.3 mmol, 3 equiv), CuCl (10 mol %), Xantphos (11 mol %), B_2_pin_2_ (2.2 equiv), NaO^*t*^Bu (2.0 equiv), ketone (0.1 mmol, 1.0 equiv), THF (0.25 M), rt.
b Yields of isolated products are listed.

Next, we investigated the reactions of allylic benzoate **1e** with aldehydes as the substrate. As shown in Scheme 4, the reactions worked generally well with a variety of aromatic and heteroaromatic aldehydes. For instance, the reactions with aromatic aldehydes with different electronic properties and substitution patterns gave secondary alcohol products **3a**-**g** in 83–98% yields. Heteroaromatic aldehydes, such as thiophene-2-carbaldehyde and furan-3-carbaldehyde, reacted under the standard conditions to give **3h**-**i** in 94–97% yields. The reaction proceeded with α,β-unsaturated aldehydes and aliphatic aldehydes, although the yields of **3j**-**l** are significantly lower than those from aromatic aldehydes.

**Scheme 4:**
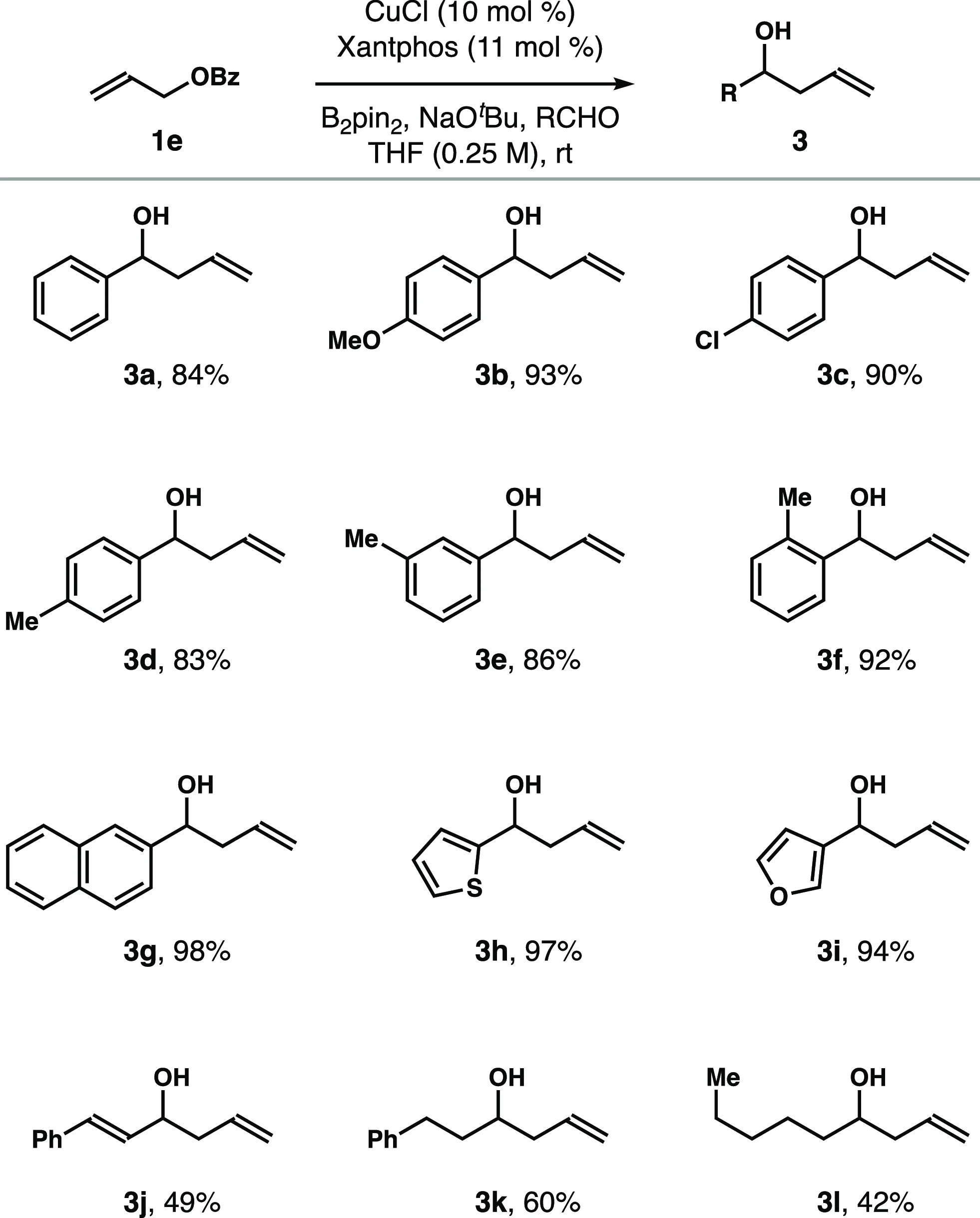
Reactions with Representative Aldehydes^a^ ^,^ ^b^

a Allylic benzoate **1e** (0.3 mmol, 3 equiv), CuCl (10 mol %), Xantphos (11 mol %), B_2_pin_2_ (2.2 equiv), NaO^*t*^Bu (2.0 equiv), ketone (0.1 mmol, 1.0 equiv), THF (0.25 M), rt.
b Yields of isolated products are listed.

We also explored the reactions with other allylic electrophiles. As illustrated in Scheme 5, the reactions of methallylic benzoate **4** with ketones gave products **5a**-**b** in 43–65% yields. The reaction with benzaldehyde gave alcohol **5c** in 31% yield. Additionally, commercially available allylic acetate **6** appended with an allylsilane unit reacted with ketones to give tertiary alcohols **7a**-**b** in 61–69% yields. A similar reaction of **6** with benzaldehyde afforded alcohol **7c** in 53% yield. Notably, the allylsilane group in the products remains intact under the reaction conditions, which provides a handle for further derivatization of products **7**.^13^

**Scheme 5:**
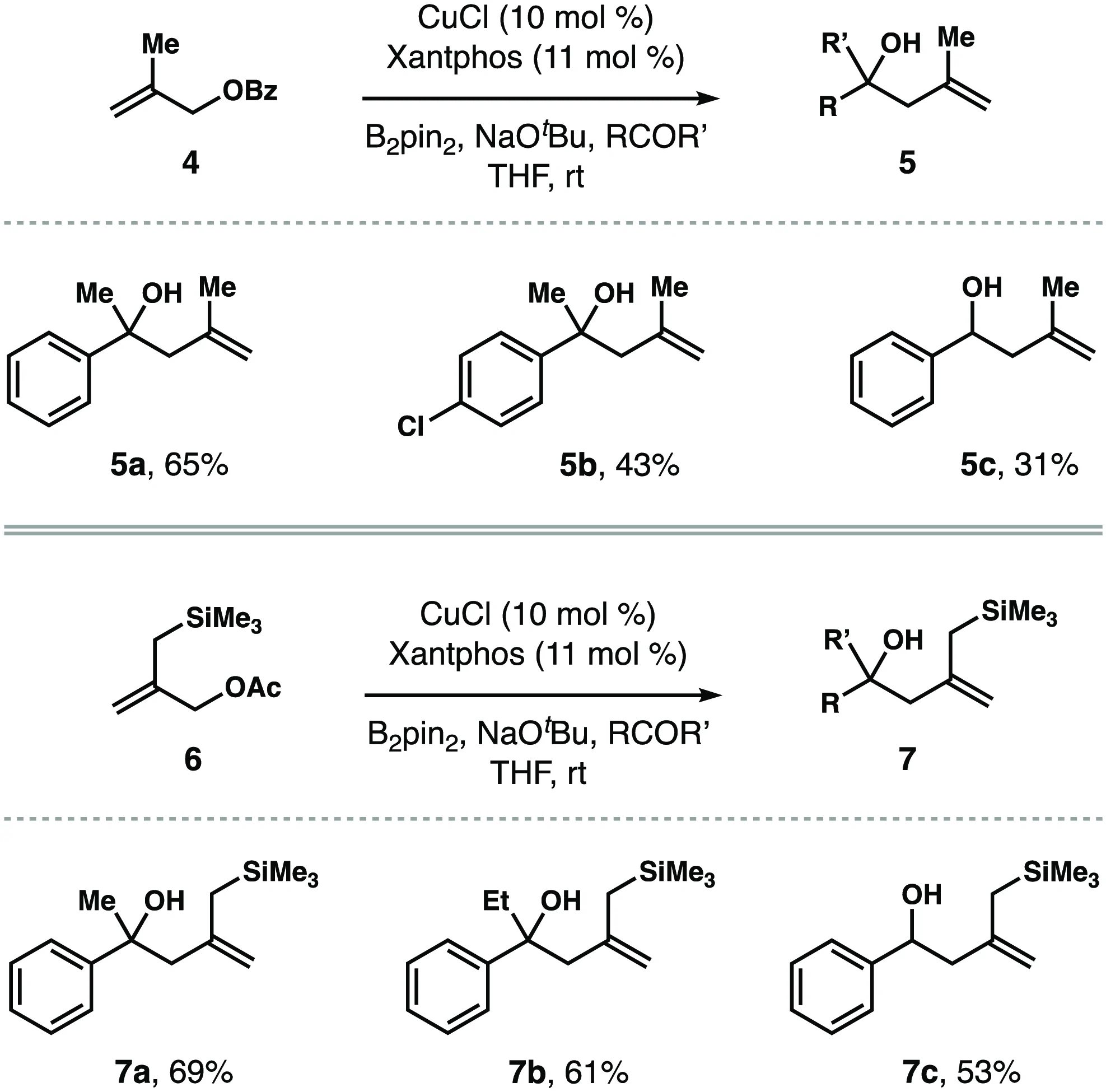
Reactions with Other Allylic Electrophiles^a^ ^,^ ^b^

a Reaction conditions: Allylic electrophile **4** (0.3 mmol, 3 equiv) or **6** (0.2 mmol, 2 equiv), CuCl (10 mol %), Xantphos (11 mol %), B_2_pin_2_ (2.2 equiv), NaO^*t*^Bu (2.0 equiv), ketone or aldehyde (0.1 mmol, 1.0 equiv), THF (0.25 M), rt.
b Yields of isolated products are listed.

As shown in Scheme 1, allylic boronate **B** was proposed to be an intermediate in the reaction process. To obtain evidence to support this analysis, we prepared allylboronate **B**. Upon exposure of **B** to the reaction conditions [CuCl (10 mol %), Xantphos (11 mol %), NaO^*t*^Bu (1.1 equiv)], product **2a** was isolated in 95% yield (Scheme 6). The results provide concrete evidence to support the intermediacy of allylboronate **B**.

**Scheme 6:**
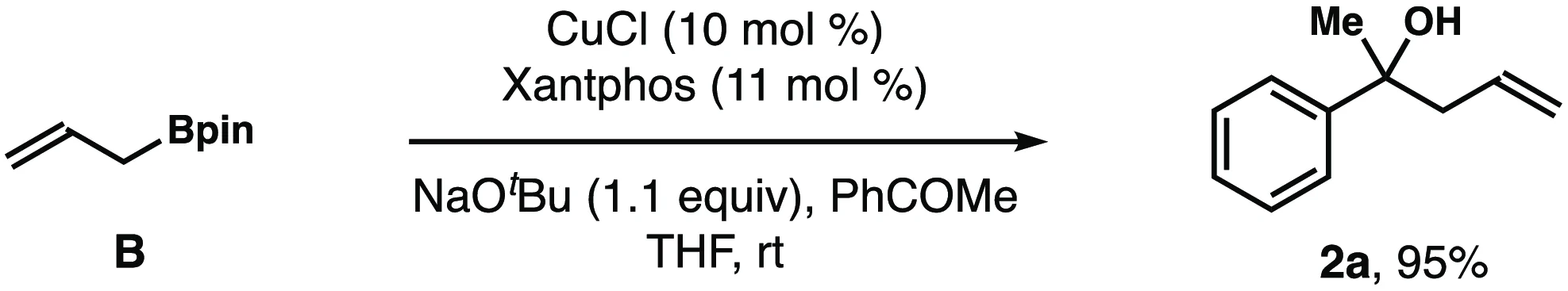
Validation of the Reaction Intermediate

To obtain enantioenriched alcohol products, we investigated catalytic asymmetric reactions with **1e** and acetophenone using an enantiomerically enriched ligand. As shown in Scheme 7, among several ligands we surveyed, the reaction with (*R*,*R*)-^*i*^PrDuphos proceeded with the highest enantioselectivity, and alcohol **2a** was isolated in 82% yield with 90:10 er. The results provide a good entry point to generate enantioenriched tertiary homoallylic alcohols using this asymmetric reductive coupling strategy.

**Scheme 7:**
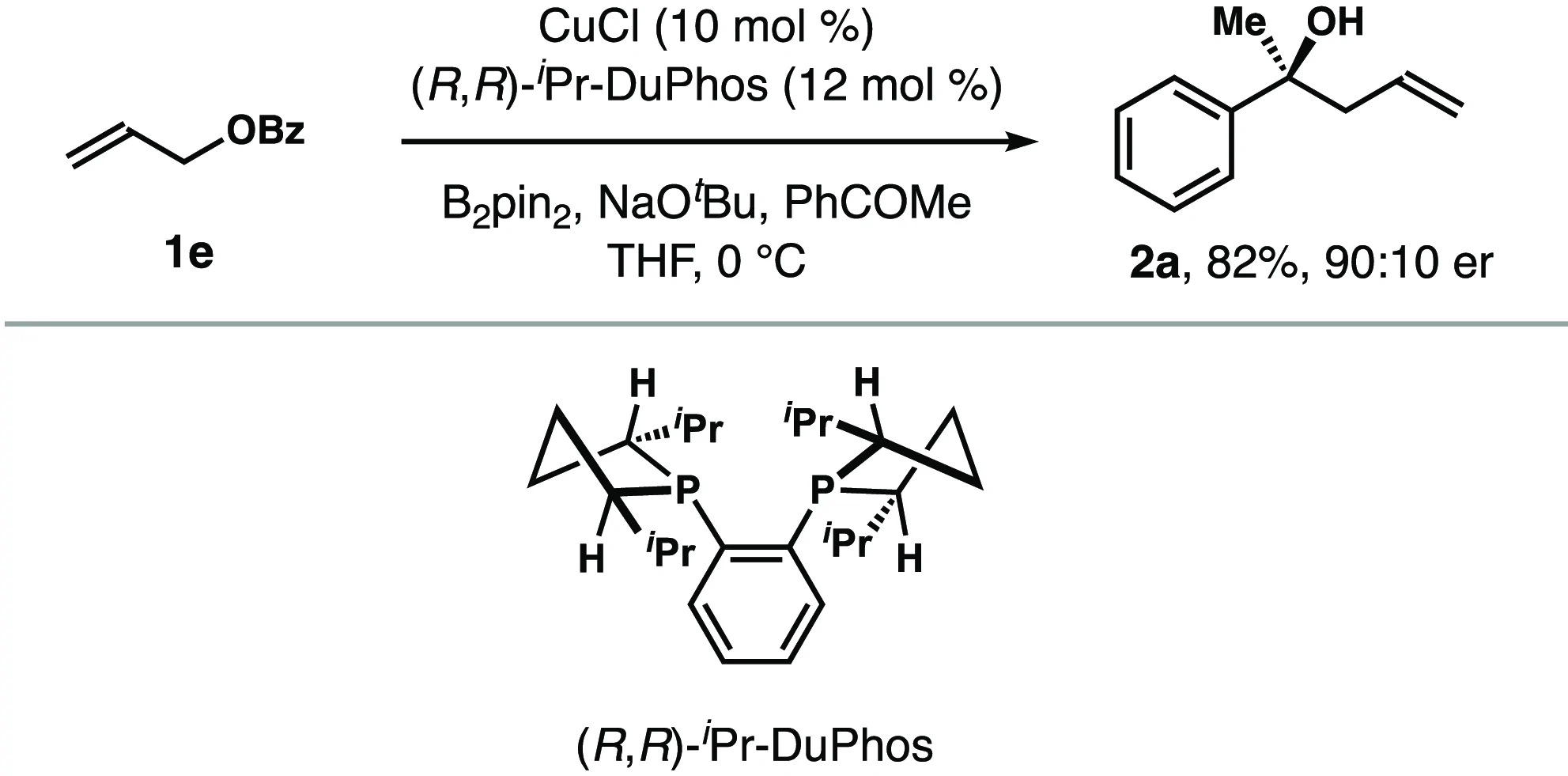
Asymmetric Reductive Coupling

In summary, we developed a protocol to synthesize secondary and tertiary homoallylic alcohols. This approach allows for the coupling of electrophilic allylic esters and carbonyl compounds with B_2_pin_2_ as the stoichiometric reductant. Meanwhile, the process employs an inexpensive and earth-abundant copper salt as the catalyst without the use of cryogenic conditions. Additionally, respectable enantioselectivity of the tertiary alcohol product was achieved from the asymmetric reductive coupling reactions. Synthetic applications of this method are currently under investigation.

## Supplementary Material



## Data Availability

The data underlying this study are available in the published article and its Supporting Information.

## Notes

Experimental procedures, characterization data (PDF)
