# Supplementary material for: Cu-Catalyzed Reductive Coupling of Allylic and Carbonyl Electrophiles
Source: Org Lett. 2026 Mar 12;28(12):3797–801. doi: 10.1021/acs.orglett.6c00497 (PMC13730441; doi:10.1021/acs.orglett.6c00497)
Supplement: Supplementary file 1 [file ol6c00497_si_001.pdf]

Cu-Catalyzed Reductive Coupling of Allylic and Carbonyl Electrophiles

*Vaughn Beale and Ming Chen**

Department of Chemistry, Virginia Tech

E-mail: mzc0102@vt.edu

Table of Contents

1. General Information *SI-2*

2. Experimental Procedures and Tabulated Spectroscopic Data *SI-3*

3. References *SI-15*

4. ^{1}H, ^{13}C, and ^{19}F Spectra *SI-16*

General Experimental Details. All reaction solvents were purified before use. Tetrahydrofuran was purified by passing through a solvent column composed of activated A-1 alumina. Unless indicated otherwise, all reactions were conducted under an atmosphere of argon using flame-dried or oven-dried (140 °C) glassware. The term "concentrated under reduced pressure" refers to the removal of solvents and other volatile materials using a rotary evaporator with the water bath temperature below 30 °C, followed by removal of the residual solvent at high vacuum (< 0.2 mbar).

Proton nuclear magnetic resonance (^{1}H NMR) spectra were acquired on commercial instruments (500, 600 MHz) at Virginia Tech NMR facility. Carbon-13 nuclear magnetic resonance (^{13}C NMR) spectra were acquired at 126 and 151 MHz. The proton signal for residual non-deuterated solvent (δ 7.26 for $CHCl_3$, δ 2.05 for acetone) was used as an internal reference for ^{1}H NMR spectra. For ^{13}C NMR spectra, chemical shifts are reported relative to the δ 77.36 resonance of $CHCl_3$ and acetone (δ 29.84). Coupling constants are reported in Hz. High-resolution mass spectra were recorded on a commercial high-resolution mass spectrometer (high-resolution hybrid QTOF) via the Micro Mass/Analytical Facility operated by the Department of Biochemistry, Virginia Tech.

Analytical thin layer chromatography (TLC) was performed on ChemScene 60 GF254 glass plates precoated with a 0.20 mm thickness of silica gel. The TLC plates were visualized with UV light and/or by staining with $KMnO_4$. Column chromatography was generally performed using Kieselgel 60 (230-400 mesh) silica gel, typically using a 50-100:1 weight ratio of silica gel to crude product.

Ketone and aldehyde substrates were purchased from commercial sources. Allylic electrophiles **1** were prepared according to the literature procedures.[1]

*General Procedure A: Synthesis of Homoallylic Alcohols **2***

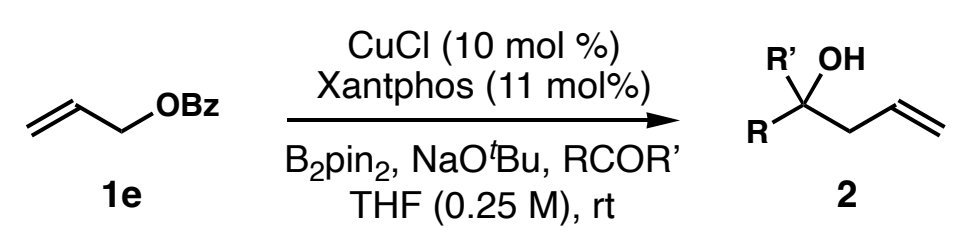

In a glovebox, an oven-dried 1-dram vial equipped with a magnetic stir bar was charged with CuCl (2 mg, 0.02 mmol, 10 mol %), Xantphos (13 mg, 0.022 mmol, 11 mol %), NaO^{t}Bu (38 mg, 0.4 mmol, 2.0 equiv), and THF (0.8 mL) under an argon atmosphere. After stirring for 10 minutes at ambient temperature, B_2pin_2 (112 mg, 0.44 mmol, 2.2 equiv) was added, and the reaction mixture was stirred for 10 minutes. Allylic benzoate **1e** [1] (97 mg, 0.6 mmol, 3.0 equiv) was then added to the reaction mixture, followed by the addition of the corresponding ketone (0.2 mmol, 1.0 equiv). The resulting mixture was stirred for 24 h at ambient temperature. Upon completion, ethyl acetate (2.0 mL) and water (2.0 mL) were added. The organic layer was separated, and the aqueous phase was extracted with ethyl acetate (2 × 2.0 mL). The combined organic extracts were filtered through a short pad of silica gel and then concentrated under reduced pressure. The crude product was purified by flash column chromatography (gradient elution with hexanes and ethyl acetate) to give product **2**.

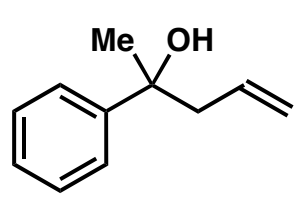

2-phenylpent-4-en-2-ol (2a) Prepared according to the general procedure A. The crude mixture was purified by flash column chromatography (gradient elution with hexane and ethyl acetate, 100:1 to 40:1) to give compound **2a** as a white solid in 98% yield (31.8 mg). ^{1}H NMR (500 MHz, $CDCl_3$) δ 7.43 – 7.46 (m, 2H), 7.33 – 7.37 (m, 2H), 7.23 – 7.26 (m, 1H), 5.60 – 5.63 (m, 1H), 5.11 – 5.16 (m, 2H), 2.67 – 2.72 (m, 1H), 2.48 – 2.53 (m, 1H), 1.55 (s, 3H). ^{13}C NMR (126 MHz, $CDCl_3$) δ 148.0, 134.0, 128.5, 127.0, 125.1, 119.8, 74.0, 48.8, 30.3. The spectral data are consistent with those reported in the literature.[2]

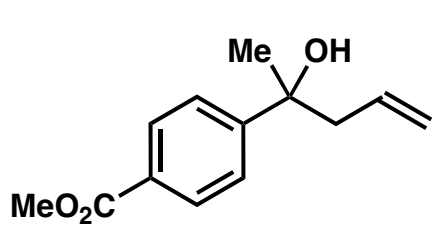

methyl 4-(2-hydroxypent-4-en-2-yl)benzoate (2b) Prepared according to the general procedure A. The crude mixture was purified by flash column chromatography (gradient elution with hexane and ethyl acetate, 20:1 to 5:1) to give compound **2b** as a colorless oil in 89% yield (39 mg). ^{1}H NMR (500 MHz, $CDCl_3$) δ 8.00 – 8.02 (m, 2H), 7.50 – 7.52 (m, 2H), 5.54 – 5.62 (m, 1H), 5.12 – 5.16 (m, 2H), 3.91 (s, 3H), 2.66 – 2.71 (m, 1H), 2.49 – 2.53 (m, 1H), 1.56 (s, 3H). ^{13}C NMR (126 MHz, $CDCl_3$) δ 167.3, 153.1, 133.4, 129.9, 128.9, 125.2, 120.4,

74.0, 52.4, 48.6, 30.2. The spectral data are consistent with those reported in the literature.[3]

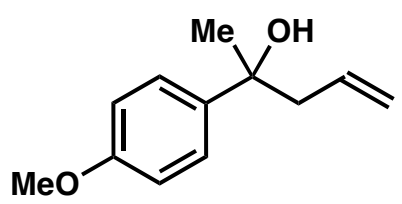

2-(4-methoxyphenyl)pent-4-en-2-ol (2c) Prepared according to the general procedure A. The crude mixture was purified by flash column chromatography (gradient elution with hexane and ethyl acetate, 50:1 to 10:1) to give compound **2c** as a colorless oil in 79% yield (30 mg). ^{1}H NMR (500 MHz, $CDCl_3$) δ 7.34 – 7.39 (m, 2H), 6.86 – 6.89 (m, 2H), 5.60 – 5.64 (m, 1H), 5.10 – 5.15 (m, 2H), 3.81 (s, 3H), 2.64 – 2.68 (m, 1H), 2.46 – 2.51 (m, 1H), 1.53 (s, 3H). ^{13}C NMR (126 MHz, $CDCl_3$) δ 158.6, 140.2, 134.2, 126.3, 119.7, 113.8, 73.7, 55.6, 48.9, 30.3. The spectral data are consistent with those reported in the literature.[4]

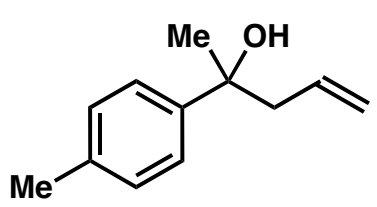

2-(*p*-tolyl)pent-4-en-2-ol (2d) Prepared according to the general procedure A. The crude mixture was purified by column chromatography (gradient elution with hexane and ethyl acetate, 100:1 to 40:1) to give compound **2d** as a colorless oil in 72% yield (25 mg). ^{1}H NMR (500 MHz, $CDCl_3$) δ 7.33 (d, J = 8.2 Hz, 2H), 7.16 (d, J = 7.9 Hz, 2H), 5.60 – 5.68 (m, 1H), 5.11 – 5.16 (m, 2H), 2.66 – 2.70 (m, 1H), 2.47 – 2.52 (m, 1H), 2.35 (s, 3H), 1.54 (s, 3H). ^{13}C NMR (126 MHz, $CDCl_3$) δ 145.1, 136.5, 134.1, 129.2, 125.0, 119.7, 73.8, 48.8, 30.3, 21.3. The spectral data are consistent with those reported in the literature.[4]

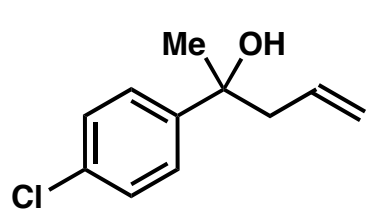

2-(4-chlorophenyl)pent-4-en-2-ol (2e) Prepared according to the general procedure A. The crude mixture was purified by column chromatography (gradient elution with hexane and ethyl acetate, 100:1 to 40:1) to give compound **2e** as a colorless oil in 92% yield (36 mg). ^{1}H NMR (500 MHz, $CDCl_3$) δ 7.36 – 7.39 (m, 2H), 7.29 – 7.32 (m, 2H), 5.56 – 5.64 (m, 1H), 5.12 – 5.16 (m, 2H), 2.63 – 2.67 (m, 1H), 2.46 – 2.51 (m, 1H), 1.53 (s, 3H). ^{13}C NMR (126 MHz, $CDCl_3$) δ 146.5, 133.6, 132.8, 128.6, 126.7, 120.3, 73.7, 48.7, 30.3. The spectral data are consistent with those reported in the literature.[5]

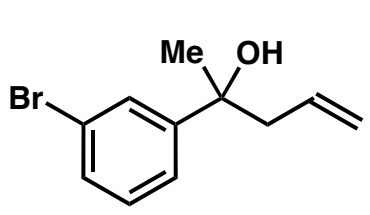

2-(3-bromophenyl)pent-4-en-2-ol (2f) Prepared according to the general procedure A. The crude mixture was purified by column chromatography (gradient elution with hexane and ethyl acetate, 100:1 to 40:1) to give compound **2f** as a white solid in 79% yield (38 mg). ^{1}H NMR (500 MHz, $CDCl_3$) δ 7.61 – 7.62 (m, 1H), 7.34 – 7.38 (m, 2H), 7.21 (t, J = 7.9 Hz, 1H), 5.56 – 5.63 (m, 1H), 5.13 – 5.17 (m, 2H), 2.63 – 2.68 (m, 1H), 2.46 – 2.50 (m, 1H), 1.53 (s, 3H). ^{13}C NMR (126

MHz, $CDCl_3$) δ 150.4, 133.4, 130.12, 130.08, 128.5, 123.9, 122.9, 120.4, 73.7, 48.7, 30.3. The spectral data are consistent with those reported in the literature.[4]

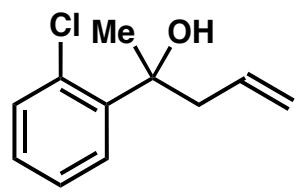

2-(2-chlorophenyl)pent-4-en-2-ol (2g) Prepared according to the general procedure A. The crude mixture was purified by column chromatography (gradient elution with hexane and ethyl acetate, 100:1 to 40:1) to give compound **2g** as a colorless oil in 84% yield (33 mg). ^{1}H NMR (500 MHz, $CDCl_3$) δ 7.69 (dd, J = 7.9, 1.7 Hz, 1H), 7.35 (dd, J = 7.9, 1.5 Hz, 1H), 7.24 – 7.28 (m, 1H), 7.17 – 7.21 (m, 1H), 5.50 – 5.59 (m, 1H), 5.08 – 5.16 (m, 2H), 3.20 – 3.24 (m, 1H), 2.61 – 2.65 (m, 1H), 1.70 (s, 3H). ^{13}C NMR (126 MHz, $CDCl_3$) δ 144.0, 134.0, 131.7, 131.1, 128.7, 128.3, 127.2, 119.7, 74.6, 45.5, 27.6. HRMS (ESI$^+$) m/z: $[M+H]^+$ Calcd for $C_{11}H_{13}ClO$ 197.0728; Found 197.0736.

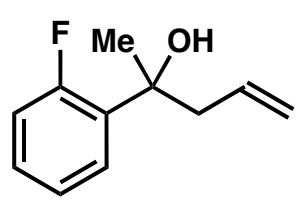

2-(2-fluorophenyl)pent-4-en-2-ol (2h) Prepared according to the general procedure A. The crude mixture was purified by column chromatography (gradient elution with hexane and ethyl acetate, 100:1 to 40:1) to give compound **2h** as a colorless oil in 75% yield (29 mg). ^{1}H NMR (500 MHz, $CDCl_3$) δ 7.52 – 7.56 (m, 1H), 7.21 – 7.26 (m, 1H), 7.11 – 7.14 (m, 1H), 7.02 (ddd, J = 12.2, 8.1, 1.2 Hz, 1H), 5.54 – 5.63 (m, 1H), 5.09 – 5.16 (m, 2H), 2.88 – 2.92 (m, 1H), 2.52 – 2.57 (m, 1H), 1.62 (s, 3H). ^{13}C NMR (126 MHz, $CDCl_3$) δ 159.7 (d, J = 245.0 Hz), 134.3 (d, J = 12.1 Hz), 134.0, 129.0 (d, J = 8.5 Hz), 127.8 (d, J = 4.6 Hz), 124.3 (d, J = 3.2 Hz), 119.9, 116.3 (d, J = 23.9 Hz), 73.0 (d, J = 4.1 Hz), 46.9 (d, J = 4.3 Hz), 28.6 (d, J = 3.6 Hz). ^{19}F NMR (376 MHz, CDCl3) δ -113.0. The spectral data are consistent with those reported in the literature.[6]

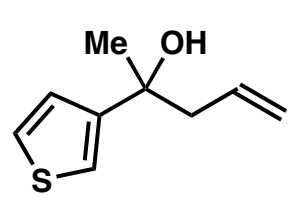

2-(thiophen-3-yl)pent-4-en-2-ol (2i) Prepared according to the general procedure A. The crude mixture was purified by column chromatography (gradient elution with hexane and ethyl acetate, 100:1 to 40:1) to give compound **2i** as a light yellow solid in 92% yield (31 mg). ^{1}H NMR (500 MHz, $CDCl_3$) δ 7.28 – 7.29 (m, 1H), 7.16 (dd, J = 3.0, 1.4 Hz, 1H), 7.06 (dd, J = 5.0, 1.3 Hz, 1H), 5.63 – 5.72 (m, 1H), 5.11 – 5.16 (m, 2H), 2.61 – 2.66 (m, 1H), 2.48 – 2.53 (m, 1H), 1.54 (s, 3H). ^{13}C NMR (126 MHz, $CDCl_3$) δ 150.0, 133.9, 126.2, 125.9, 119.8, 119.7, 73.0, 48.6, 29.8. The spectral data are consistent with those reported in the literature.[7]

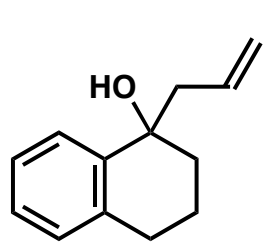

1-allyl-1,2,3,4-tetrahydronaphthalen-1-ol (2j) Prepared according to the general procedure A. The crude mixture was purified by flash column

chromatography (gradient elution with hexane and ethyl acetate, 100:1 to 40:1) to give compound **2j** as a colorless oil in 66% yield (25 mg). ^{1}H NMR (500 MHz, $CDCl_3$) δ 7.56 (dd, *J* = 7.7, 1.5 Hz, 1H), 7.16 – 7.24 (m, 2H), 7.08 (dd, *J* = 7.6, 1.4 Hz, 1H), 5.79 – 5.84 (m, 1H), 5.12 – 5.16 (m, 2H), 2.75 – 2.80 (m, 2H), 2.61 (d, *J* = 7.4 Hz, 2H), 2.02 – 2.06 (m, 1H), 1.80 – 1.90 (m, 3H). ^{13}C NMR (126 MHz, $CDCl_3$) δ 142.2, 137.1, 134.4, 129.2, 127.5, 126.7, 126.6, 119.0, 72.3, 47.4, 36.5, 30.1, 20.1. The spectral data are consistent with those reported in the literature.[8]

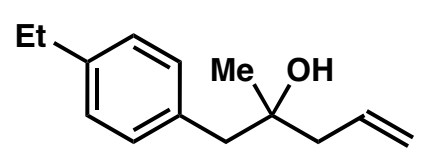

1-(4-ethylphenyl)-2-methylpent-4-en-2-ol (2k) Prepared according to the general procedure A. The crude mixture was purified by flash column chromatography (gradient elution with hexane and ethyl acetate, 100:1 to 40:1) to give compound **2k** as a colorless oil in 42% yield (17 mg). ^{1}H NMR (500 MHz, $CDCl_3$) δ 7.12 – 7.16 (m, 4H), 5.89 – 5.98 (m, 1H), 5.11 – 5.18 (m, 2H), 2.77 (d, *J* = 13.4 Hz, 1H), 2.71 (d, *J* = 13.4 Hz, 1H), 2.64 (q, *J* = 7.6 Hz, 2H), 2.21 – 2.29 (m, 2H), 1.24 (t, *J* = 7.6 Hz, 3H), 1.15 (s, 3H). ^{13}C NMR (126 MHz, $CDCl_3$) δ 142.8, 134.8, 134.5, 130.9, 128.1, 119.0, 72.4, 47.8, 46.6, 28.8, 26.9, 15.9. HRMS (ESI^+) m/z: $[M+H]^+$ Calcd for $C_{14}H_{20}O$ 205.1587; Found 205.1591.

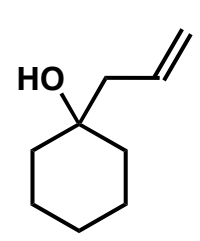

1-allylcyclohexan-1-ol (2l) Prepared according to the general procedure A. The crude mixture was purified by flash column chromatography (gradient elution with hexane and ethyl acetate, 100:1 to 50:1) to give compound **2l** as a colorless oil in 50% yield (14 mg). ^{1}H NMR (500 MHz, $CDCl_3$) δ 5.84 – 5.91 (m, 1H), 5.08 – 5.16 (m, 2H), 2.20 – 2.22 (m, 2H), 1.40 – 1.63 (m, 8H), 1.24 – 1.30 (m, 2H). ^{13}C NMR (126 MHz, $CDCl_3$) δ 134.1, 119.0, 71.3, 47.0, 37.7, 26.1, 22.5. The spectral data are consistent with those reported in the literature.[7]

*General Procedure B: Synthesis of Homoallylic Alcohols **3***

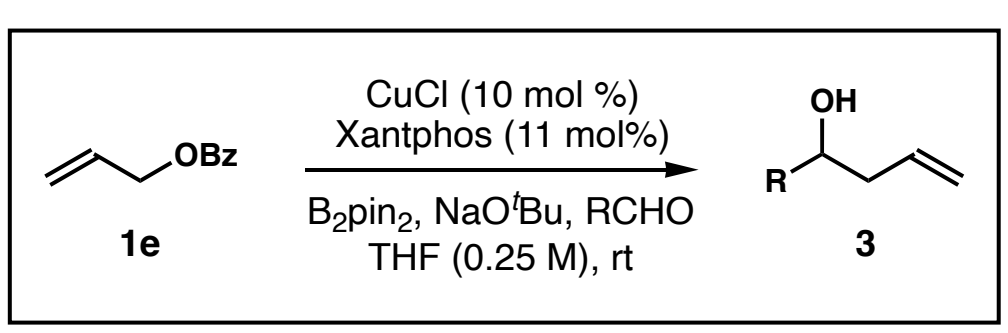

In a glovebox, an oven-dried 1-dram vial equipped with a magnetic stir bar was charged with CuCl (2 mg, 0.02 mmol, 10 mol %), Xantphos (13 mg, 0.022 mmol, 11 mol %), NaO^{t}Bu (38 mg, 0.4 mmol, 2.0 equiv), and THF (0.8 mL) under an argon atmosphere.

After stirring for 10 minutes at ambient temperature, B_2pin_2 (112 mg, 0.44 mmol, 2.2 equiv) was added, and the reaction mixture was stirred for 10 minutes. Allylic benzoate **1e** (97 mg, 0.6 mmol, 3.0 equiv) was then added to the reaction mixture, followed by the addition of the corresponding aldehyde (0.2 mmol, 1.0 equiv). The resulting mixture was stirred for 24 h at ambient temperature. Upon completion, ethyl acetate (2.0 mL) and water (2.0 mL) were added. The organic layer was separated, and the aqueous phase was extracted with ethyl acetate (2 × 2.0 mL). The combined organic extracts were filtered through a short pad of silica gel and then concentrated under reduced pressure. The crude product was purified by flash column chromatography (gradient elution with hexanes and ethyl acetate) to give product **3**.

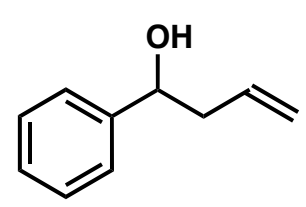

1-phenylbut-3-en-1-ol (3a) Prepared according to the general procedure B. The crude mixture was purified by flash column chromatography (gradient elution with hexane and ethyl acetate, 100:1 to 30:1) to give compound **3a** as a colorless oil in 84% yield (25 mg). ^{1}H NMR (500 MHz, $CDCl_3$) δ 7.34 – 7.38 (m, 4H), 7.27 – 7.30 (m, 1H), 5.78 – 5.86 (m, 1H), 5.13 – 5.20 (m, 2H), 4.75 (dd, *J* = 7.8, 5.1 Hz, 1H), 2.47 – 2.56 (m, 2H). ^{13}C NMR (126 MHz, $CDCl_3$) δ 144.2, 134.8, 128.8, 127.9, 126.2, 118.8, 73.6, 44.2. The spectral data are consistent with those reported in the literature.[9]

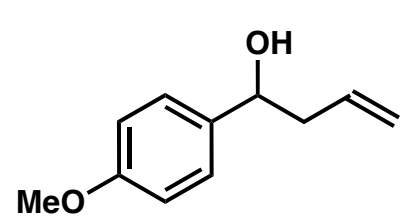

1-(4-methoxyphenyl)but-3-en-1-ol (3b) Prepared according to the general procedure B. The crude mixture was purified by flash column chromatography (gradient elution with hexane and ethyl acetate, 50:1 to 10:1) to give compound **3b** as a colorless oil in 93% yield (33 mg). ^{1}H NMR (500 MHz, $CDCl_3$) δ 7.27 – 7.30 (m, 2H), 6.87 – 6.90 (m, 2H), 5.76 – 5.85 (m, 1H), 5.12 – 5.18 (m, 2H), 4.70 (t, *J* = 6.5 Hz, 1H), 3.81 (s, 3H), 2.49 – 2.52 (m, 2H). ^{13}C NMR (126 MHz, $CDCl_3$) δ 159.4, 136.4, 135.0, 127.4, 118.6, 114.2, 73.3, 55.6, 44.1. The spectral data are consistent with those reported in the literature.[9]

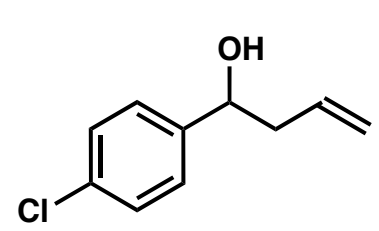

1-(4-chlorophenyl)but-3-en-1-ol (3c) Prepared according to the general procedure B. The crude mixture was purified by column chromatography (gradient elution with hexane and ethyl acetate, 100:1 to 40:1) to give compound **3c** as a colorless oil in 90% yield (33 mg). ^{1}H NMR (500 MHz, $CDCl_3$) δ 7.29 – 7.34 (m, 4H), 5.74 – 5.83 (m, 1H), 5.15 – 5.19 (m, 2H), 4.73 (dd, *J* = 7.9, 4.9 Hz, 1H), 2.42 – 2.54 (m, 2H). ^{13}C NMR (126 MHz, $CDCl_3$) δ 142.6, 134.3, 133.5, 128.9, 127.6, 119.3, 72.9, 44.2. The spectral data are consistent with those reported in the literature.[10]

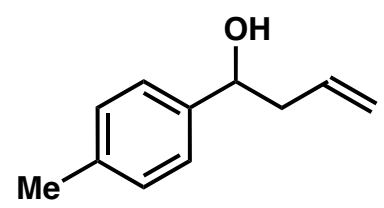

1-(*p*-tolyl)but-3-en-1-ol (3d) Prepared according to the general procedure B. The crude mixture was purified by column chromatography (gradient elution with hexane and ethyl acetate, 100:1 to 40:1) to give compound **3d** as a colorless oil in 83% yield (27 mg). ^{1}H NMR (500 MHz, $CDCl_3$) δ 7.25 (d, J = 7.7 Hz, 2H), 7.16 (d, J = 7.9 Hz, 2H), 5.77 – 5.85 (m, 1H), 5.12 – 5.19 (m, 2H), 4.71 (dd, J = 7.5, 5.5 Hz, 1H), 2.46 – 2.54 (m, 2H), 2.35 (s, 3H). ^{13}C NMR (126 MHz, $CDCl_3$) δ 141.3, 137.6, 134.9, 129.5, 126.1, 118.7, 73.5, 44.1, 21.5. The spectral data are consistent with those reported in the literature.[9]

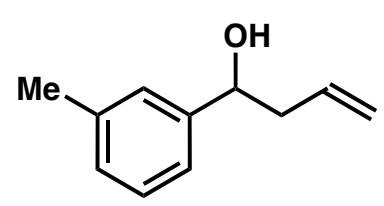

1-(*m*-tolyl)but-3-en-1-ol (3e) Prepared according to the general procedure B. The crude mixture was purified by column chromatography (gradient elution with hexane and ethyl acetate, 100:1 to 40:1) to give compound **3e** as a colorless oil in 86% yield (28 mg). ^{1}H NMR (500 MHz, $CDCl_3$) δ 7.23 – 7.26 (m, 1H), 7.14 – 7.19 (m, 2H), 7.08 – 7.10 (m, 1H), 5.78 – 5.85 (m, 1H), 5.13 – 5.20 (m, 2H), 4.71 (dd, J = 7.8, 5.1 Hz, 1H), 2.49 – 2.53 (m, 2H), 2.36 (s, 3H). ^{13}C NMR (126 MHz, $CDCl_3$) δ 144.2, 138.4, 134.9, 128.68, 128.66, (zooming the spectrum) 126.8, 123.2, 118.7, 73.7, 44.2, 21.8. The spectral data are consistent with those reported in the literature.[11]

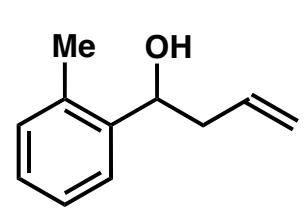

1-(*o*-tolyl)but-3-en-1-ol (3f) Prepared according to the general procedure B. The crude mixture was purified by flash column chromatography (gradient elution with hexane and ethyl acetate, 100:1 to 40:1) to give compound **3f** as a light yellow solid in 92% yield (30 mg). ^{1}H NMR (500 MHz, $CDCl_3$) δ 7.49 (dd, J = 7.7, 1.4 Hz, 1H), 7.22 – 7.25 (m, 1H), 7.13 – 7.19 (m, 2H), 5.82 – 5.89 (m, 1H), 5.15 – 5.22 (m, 2H), 4.98 (dd, J = 8.3, 4.3 Hz, 1H), 2.42 – 2.53 (m, 2H), 2.35 (s, 3H). ^{13}C NMR (126 MHz, $CDCl_3$) δ 142.3, 135.1, 134.7, 130.7, 127.6, 126.6, 125.5, 118.7, 70.0, 43.0, 19.4. The spectral data are consistent with those reported in the literature.[9]

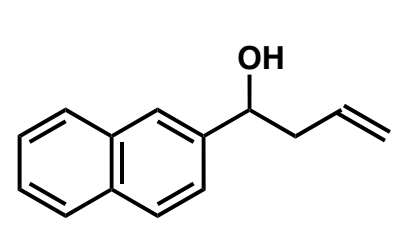

1-(naphthalen-2-yl)but-3-en-1-ol (3g) Prepared according to the general procedure B. The crude mixture was purified by flash column chromatography (gradient elution with hexane and ethyl acetate, 100:1 to 40:1) to give compound **3g** as a white solid in 98% yield (40 mg). ^{1}H NMR (500 MHz, $CDCl_3$) δ 7.81 – 7.85 (m, 4H), 7.45 – 7.50 (m, 3H), 5.80 – 5.87 (m, 1H), 5.15 – 5.22 (m, 2H), 4.92 (dd, J = 7.8, 5.0 Hz, 1H), 2.57 – 2.64 (m, 2H). ^{13}C NMR (126 MHz, $CDCl_3$) δ 141.6, 134.7, 133.6, 133.3, 128.6, 128.3, 128.0, 126.5, 126.2, 124.9, 124.3, 119.0, 73.7, 44.1. The spectral data are consistent with those reported in the literature.[12]

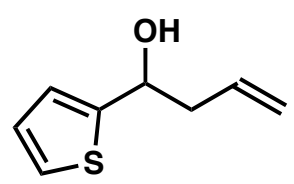

1-(thiophen-2-yl)but-3-en-1-ol (3h) Prepared according to the general procedure B. The crude mixture was purified by column (gradient elution with hexane and ethyl acetate, 100:1 to 40:1) to give compound **3h** as a colorless oil in 97% yield (30 mg). ^{1}H NMR (500 MHz, $CDCl_3$) δ 7.25 – 7.26 (m, 1H), 6.97 – 7.00 (m, 2H), 5.80 – 5.88 (m, 1H), 5.16 – 5.22 (m, 2H), 4.99 – 5.02 (m, 1H), 2.59 – 2.67 (m, 2H). ^{13}C NMR (126 MHz, $CDCl_3$) δ 148.1, 134.2, 127.0, 124.9, 124.0, 119.2, 69.7, 44.1. The spectral data are consistent with those reported in the literature.[9]

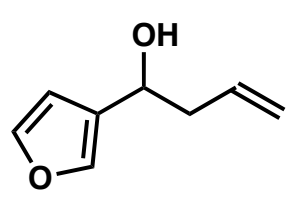

1-(furan-3-yl)but-3-en-1-ol (3i) Prepared according to the general procedure B. The crude mixture was purified by column chromatography (gradient elution with hexane and ethyl acetate, 100:1 to 40:1) to give compound **3i** as a colorless oil in 94% yield (26 mg). ^{1}H NMR (500 MHz, $CDCl_3$) δ 7.39 – 7.41 (m, 2H), 6.41 – 6.42 (m, 1H), 5.77 – 5.88 (m, 1H), 5.14 – 5.21 (m, 2H), 4.73 (dd, J = 7.4, 5.4 Hz, 1H), 2.48 – 2.57 (m, 2H). ^{13}C NMR (126 MHz, $CDCl_3$) δ 143.7, 139.4, 134.4, 128.8, 119.0, 108.9, 66.4, 42.8. The spectral data are consistent with those reported in the literature.[13]

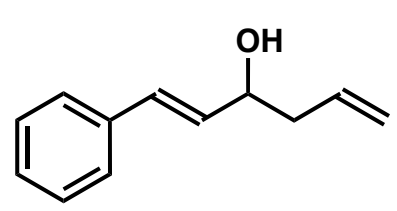

(*E*)-1-phenylhexa-1,5-dien-3-ol (3j) Prepared according to the general procedure B. The crude mixture was purified by flash column chromatography (gradient elution with hexane and ethyl acetate, 100:1 to 30:1) to give compound **3j** as a colorless oil in 49% yield (17 mg). ^{1}H NMR (500 MHz, $CDCl_3$) δ 7.38 – 7.40 (m, 2H), 7.32 (dd, J = 8.5, 6.8 Hz, 2H), 7.23 – 7.26 (m, 1H), 6.61 (d, J = 16.0, 1.2 Hz, 1H), 6.25 (dd, J = 15.9, 6.3 Hz, 1H), 5.82 – 5.91 (m, 1H), 5.16 – 5.21 (m, 2H), 4.35 – 4.39 (m, 1H), 2.37 – 2.47 (m, 2H). ^{13}C NMR (126 MHz, $CDCl_3$) δ 137.0, 134.4, 131.9, 130.7, 128.9, 128.0, 126.8, 118.9, 72.0, 42.4. The spectral data are consistent with those reported in the literature.[9]

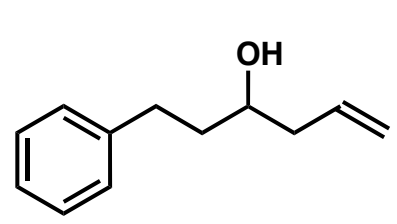

1-phenylhex-5-en-3-ol (3k) Prepared according to the general procedure B. The crude mixture was purified by flash column chromatography (gradient elution with hexane and ethyl acetate, 100:1 to 25:1) to give compound **3k** as a colorless oil in 60% yield (21 mg). ^{1}H NMR (500 MHz, $CDCl_3$) δ 7.27 – 7.30 (m, 2H), 7.17 – 7.22 (m, 3H), 5.81 – 5.83 (m, 1H), 5.13 – 5.17 (m, 2H), 3.67 – 3.69 (m, 1H), 2.81 (ddd, J = 13.7, 8.9, 6.6 Hz, 1H), 2.69 (ddd, J = 13.7, 9.0, 7.3 Hz, 1H), 2.31 – 2.33 (m, 1H), 2.17 – 2.22 (m, 1H), 1.77 – 1.82 (m, 2H). ^{13}C NMR (126 MHz, $CDCl_3$) δ 142.4, 134.9, 128.79, 128.75, 126.2, 118.7, 70.3, 42.4, 38.8, 32.4. The spectral data are consistent with those reported in the literature.[9]

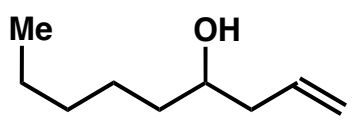

non-1-en-4-ol (3l) Prepared according to the general procedure B. The crude mixture was purified by column chromatography (gradient elution with hexane and ethyl acetate, 100:1 to 40:1) to give compound **3l** as a colorless oil in 42% yield (12 mg). ^{1}H NMR (500 MHz, $CDCl_3$) δ 5.81 – 5.85 (m, 1H), 5.12 – 5.16 (m, 2H), 3.63 – 3.66 (m, 1H), 2.28 – 2.30 (m, 1H), 2.13 – 2.17 (m, 1H), 1.44 – 1.47 (m, 2H), 1.25 – 1.34 (m, 6H), 0.88 – 0.91 (m, 3H). ^{13}C NMR (126 MHz, $CDCl_3$) δ 135.3, 118.4, 71.0, 42.3, 37.1, 32.2, 25.7, 23.0, 14.4. The spectral data are consistent with those reported in the literature.[9]

General Procedure C: Synthesis of Homoallylic Alcohols ***5***

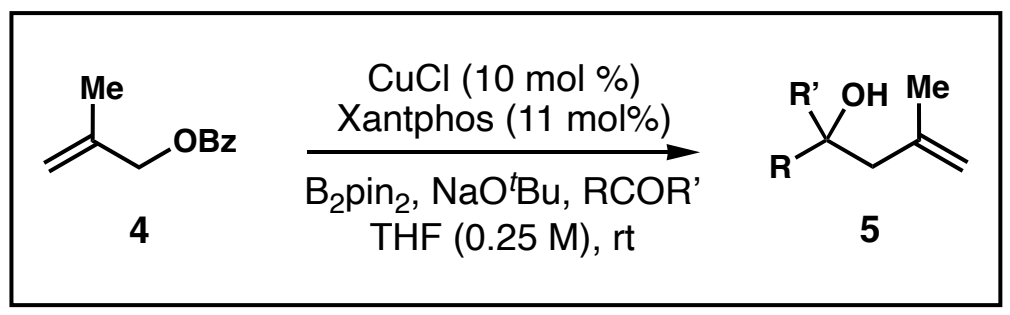

In a glovebox, an oven-dried 1-dram vial equipped with a magnetic stir bar was charged with CuCl (2 mg, 0.02 mmol, 10 mol %), Xantphos (13 mg, 0.022 mmol, 11 mol %), NaO^{t}Bu (38 mg, 0.4 mmol, 2.0 equiv), and THF (0.8 mL) under an argon atmosphere. After stirring for 10 minutes at ambient temperature, B_2pin_2 (112 mg, 0.44 mmol, 2.2 equiv) was added, and the reaction mixture was stirred for 10 minutes. Allylic electrophile **4** (106 mg, 0.6 mmol, 3.0 equiv) was then added to the reaction mixture, followed by the addition of the corresponding ketone or aldehyde (0.2 mmol, 1.0 equiv). The resulting mixture was stirred for 24 h at ambient temperature. Upon completion, ethyl acetate (2.0 mL) and water (2.0 mL) were added. The organic layer was separated, and the aqueous phase was extracted with ethyl acetate (2 × 2.0 mL). The combined organic extracts were filtered through a short pad of silica gel and then concentrated under reduced pressure. The crude product was purified by flash column chromatography (gradient elution with hexanes and ethyl acetate) to give product **5**.

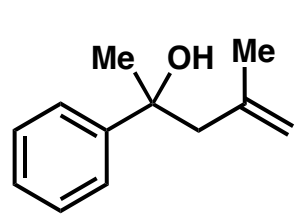

4-methyl-2-phenylpent-4-en-2-ol (5a) Prepared according to the general procedure C. The crude mixture was purified by column chromatography (gradient elution with hexanes and ethyl acetate, 100:1 to 40:1) to give compound **5a** as a colorless oil in 65% yield (23 mg). ^{1}H NMR (500 MHz, $CDCl_3$) δ 7.43 – 7.47 (m, 2H), 7.31 – 7.35 (m, 2H), 7.21 – 7.24 (m, 1H), 4.89 – 4.90 (m, 1H), 4.74 – 4.75 (m, 1H), 2.62 – 2.66 (m, 1H), 2.51 – 2.54 (m, 1H), 1.57 (s, 3H), 1.40 (s, 3H). ^{13}C NMR (126 MHz, $CDCl_3$) δ 148.3, 142.9, 128.4, 126.8, 125.1, 116.0, 73.5, 52.4, 31.1, 24.5. The spectral data are consistent with those reported in the literature.[14]

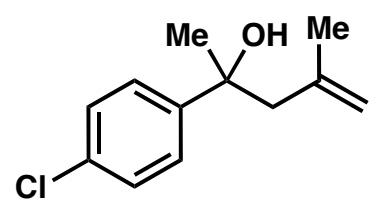

2-(4-chlorophenyl)-4-methylpent-4-en-2-ol (5b) Prepared according to the general procedure C. The crude mixture was purified by flash column chromatography (gradient elution with hexanes and ethyl acetate, 100:1 to 40:1) to give compound **5b** as a colorless oil in 43% yield (18 mg). ^{1}H NMR (500 MHz, $CDCl_3$) δ 7.37 – 7.40 (m, 2H), 7.28 – 7.31 (m, 2H), 4.90 – 4.91 (m, 1H), 4.740 – 4.745 (m, 1H), 2.59 – 2.63 (m, 1H), 2.49 – 2.52 (m, 1H), 1.54 (s, 3H), 1.42 (s, 3H). ^{13}C NMR (126 MHz, $CDCl_3$) δ 147.0, 142.5, 132.7, 128.5, 126.7, 116.4, 73.3, 52.3, 31.2, 24.6. HRMS (ESI$^+$) m/z: $[M–H_2O+H]^+$ Calcd for $C_{12}H_{14}Cl$ 193.0779; Found 193.0785.

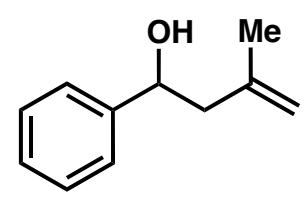

3-methyl-1-phenylbut-3-en-1-ol (5c) Prepared according to the general procedure C. The crude mixture was purified by column chromatography (gradient elution with hexane and ethyl acetate, 100:1 to 40:1) to give compound **5c** as a colorless oil in 31% yield (10 mg). ^{1}H NMR (500 MHz, $CDCl_3$) δ 7.34 – 7.40 (m, 4H), 7.26 – 7.29 (m, 1H), 4.93 – 4.94 (m, 1H), 4.86 – 4.87 (m, 1H), 4.82 (t, *J* = 6.8 Hz, 1H), 2.43 (d, *J* = 7.1 Hz, 2H), 1.81 (s, 3H). ^{13}C NMR (126 MHz, $CDCl_3$) δ 144.4, 142.8, 128.8, 127.8, 126.1, 114.5, 71.8, 48.8, 22.7. The spectral data are consistent with those reported in the literature.[14]

General Procedure D: Synthesis of Homoallylic Alcohols 7

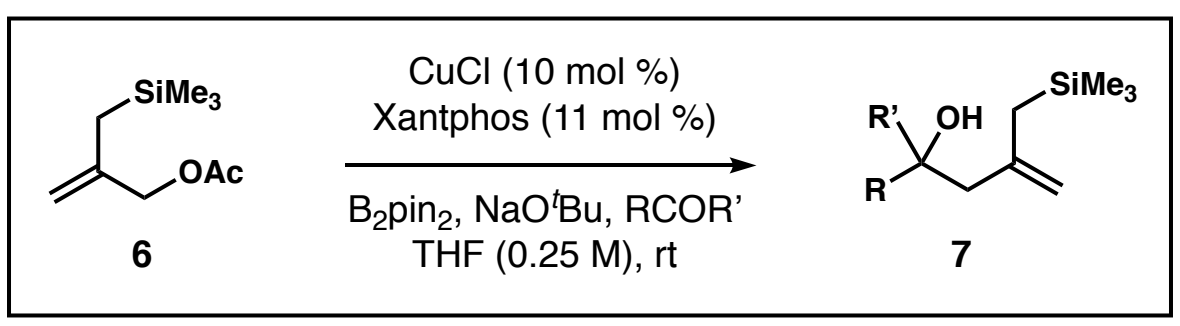

In a glovebox, an oven-dried 1-dram vial equipped with a magnetic stir bar was charged with CuCl (2 mg, 0.02 mmol, 10 mol %), Xantphos (13 mg, 0.022 mmol, 11 mol %), NaO^{t}Bu (38 mg, 0.4 mmol, 2.0 equiv), and THF (0.8 mL) under an argon atmosphere. After stirring for 10 minutes at ambient temperature, B_2pin_2 (112 mg, 0.44 mmol, 2.2 equiv) was added, and the reaction mixture was stirred for 10 minutes. Allylic electrophile **6** (75 mg, 0.4 mmol, 2.0 equiv) was then added to the reaction mixture, followed by the addition of the corresponding ketone or aldehyde (0.2 mmol, 1.0 equiv). The resulting mixture was stirred for 24 h at ambient temperature. Upon completion, ethyl acetate (2.0 mL) and water (2.0 mL) were added. The organic layer was separated, and the aqueous phase was extracted with ethyl acetate (2 × 2.0 mL). The combined organic extracts were filtered through a short pad of silica gel and then concentrated

under reduced pressure. The crude product was purified by flash column chromatography (gradient elution with hexanes and ethyl acetate) to give product **7**.

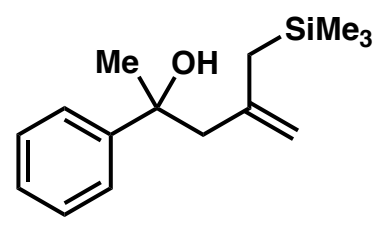

2-phenyl-4-((trimethylsilyl)methyl)pent-4-en-2-ol (7a) Prepared according to the general procedure D. The crude mixture was purified by flash column chromatography to give compound **7a** in 69% yield as colorless oil. A 1 mmol-scale reaction was also conducted, and **7a** was isolated 73% yield (181 mg). ^{1}H NMR (600 MHz, d^6-acteone) δ 7.50 (d, *J* = 7.4 Hz, 2H), 7.29 (dd, *J* = 7.6, 7.6 Hz, 2H), 7.18 (dd, *J* = 7.2, 7.2 Hz, 1H), 4.57 (s, 1H), 4.55 (s, 1H), 3.84 (s, 1H), 2.49 (d, *J* = 13.4 Hz, 1H), 2.42 (d, *J* = 13.3 Hz, 1H), 1.54 (s, 3H), 1.44 (s, 2H), -0.04 (s, 9H). ^{13}C NMR (151 MHz, d^6-acteone) δ 150.1, 145.3, 128.4, 126.8, 126.0, 112.0, 74.3, 53.1, 30.1 (*via* DEPT 135), 28.3, -1.3. HRMS (ESI$^+$) m/z: [M+Na]$^+$ Calcd for $C_{15}H_{24}OSiNa$ 271.1494; Found: 271.1506.

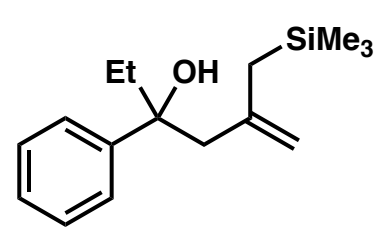

phenyl-5-((trimethylsilyl)methyl)hex-5-en-3-ol (7b) Prepared according to the general procedure D. The crude mixture was purified by flash column chromatography to give compound **7b** in 61% yield as colorless oil. ^{1}H NMR (600 MHz, d^6-acteone) δ 7.45 (d, *J* = 7.6 Hz, 2H), 7.30 (dd, *J* = 7.7, 7.7 Hz, 2H), 7.18 (t, *J* = 7.3, 7.3 Hz, 1H), 4.55 (s, 1H), 4.54 (s, 1H), 3.49 (s, 1H), 2.54 (d, *J* = 13.4 Hz, 1H), 2.47 (d, *J* = 13.4 Hz, 1H), 1.89 – 2.0 (m, 1H), 1.76 – 1.89 (m, 1H), 1.44 (d, *J* = 13.2 Hz, 1H), 1.22 (d, *J* = 13.2 Hz, 1H), 0.69 (t, *J* = 7.4 Hz, 3H), -0.06 (s, 9H). ^{13}C NMR (151 MHz, d^6-acteone) δ 147.6, 145.0, 128.3, 126.6(two overlapping carbon signals), 112.1, 76.6, 52.1, 35.9, 28.4, 8.2, -1.3. HRMS (EI$^+$) m/z: [M]$^+$ Calcd for $C_{16}H_{26}OSi$ 262.1753; Found 262.1743.

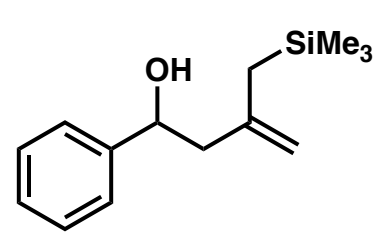

1-phenyl-3-((trimethylsilyl)methyl)but-3-en-1-ol (7c) Prepared according to the general procedure D. The crude mixture was purified by flash column chromatography to give compound **7c** in 53% yield as colorless oil. ^{1}H NMR (600 MHz, $CDCl_3$) δ 7.32 – 7.37 (m, 4H), 7.24 – 7.27 (m, 1H), 4.77 (s, 2H), 4.73 (s, 1H), 2.29 – 2.36 (m, 3H), 1.62 (d, *J* = 13.1 Hz, 1H), 1.56 (d, *J* = 13.5 Hz, 1H), 0.02 (s, 9H). ^{13}C NMR (151 MHz, $CDCl_3$) δ 144.7, 144.2, 128.7, 127.8, 126.1, 111.5, 71.3, 49.5, 26.7, −1.0. HRMS (ESI) m/z: [M+Na]$^+$ Calcd for $C_{14}H_{22}OSiNa$ 257.1338; Found: 257.1334.

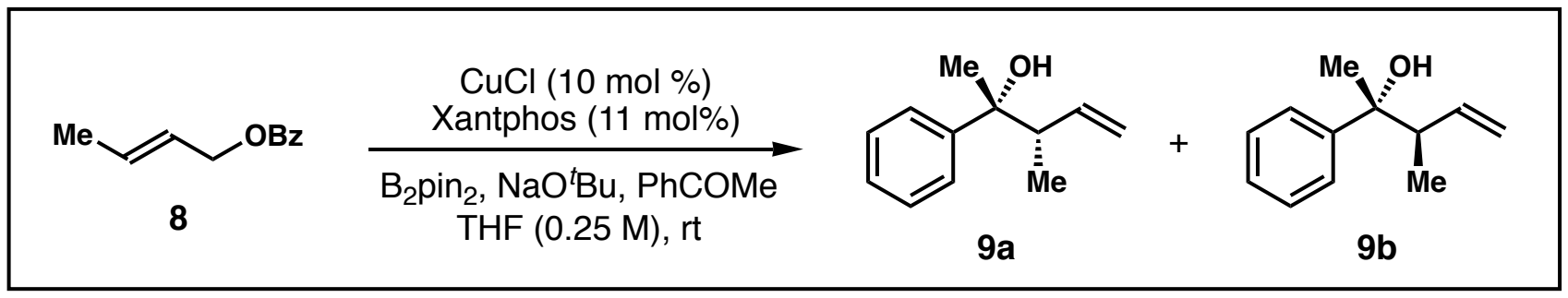

3-methyl-2-phenylpent-4-en-2-ol (9) In a glovebox, an oven-dried 1-dram vial equipped with a magnetic stir bar was charged with CuCl (2 mg, 0.02 mmol, 10 mol %), Xantphos (13 mg, 0.022 mmol, 11 mol %), NaO^{t}Bu (38 mg, 0.4 mmol, 2.0 equiv), and THF (0.8 mL) under an argon atmosphere. After stirring for 10 minutes at ambient temperature, B_2pin_2 (112 mg, 0.44 mmol, 2.2 equiv) was added, and the reaction mixture was stirred for 10 minutes. Allylic benzoate **8** (106 mg, 0.6 mmol, 3.0 equiv) was then added to the reaction mixture, followed by the addition of acetophenone (24 mg, 0.2 mmol, 1.0 equiv). The resulting mixture was stirred for 24 h at ambient temperature. Upon completion, ethyl acetate (2.0 mL) and water (2.0 mL) were added. The organic layer was separated, and the aqueous phase was extracted with ethyl acetate (2 × 2.0 mL). The combined organic extracts were filtered through a short pad of silica gel and then concentrated under reduced pressure. The crude product was purified by flash column chromatography (gradient elution with hexane and ethyl acetate, 100:1 to 40:1) to give a 3:1 mixture of **9a** and **9b** as colorless oil in 71% yield (25 mg). Major product **9a**: ^{1}H NMR (500 MHz, $CDCl_3$) δ 7.40 – 7.45 (m, 2H, overlapped with **9b**), 7.32 – 7.36 (m, 2H, overlapped with **9b**), 7.22 – 7.26 (m, 1H, overlapped with **9b**), 5.83 (ddd, J = 17.0, 10.5, 8.2 Hz, 1H), 5.09 – 5.13 (m, 2H, overlapped with **9b**), 2.52 – 2.62 (m, 1H, overlapped with **9b**), 1.54 (s, 3H, overlapped with **9b**), 0.87 (d, J = 6.9 Hz, 3H). ^{13}C NMR (126 MHz, $CDCl_3$) δ 147.3, 140.2, 128.2 (overlapped with **9b**), 126.8, 125.5, 116.7, 76.1, 49.3, 28.9, 15.1. Minor product **9b**: ^{1}H NMR (500 MHz, $CDCl_3$) δ 7.40 – 7.45 (m, 2H, overlapped with **9a**), 7.32 – 7.36 (m, 2H, overlapped with **9a**), 7.22 – 7.26 (m, 1H, overlapped with **9a**), 5.72 (ddd, J = 17.1, 10.6, 7.6 Hz, 1H), 5.09 – 5.13 (m, 2H, overlapped with **9a**), 2.52 – 2.62 (m, 1H, overlapped with **9a**), 1.54 (s, 3H, overlapped with **9a**), 0.97 (d, J = 6.9 Hz, 3H). ^{13}C NMR (126 MHz, $CDCl_3$) δ 147.4, 140.3, 128.2 (overlapped with **9a**), 127.0, 125.8, 117.0, 76.0, 49.1, 26.2, 14.5. The spectral data are consistent with those reported in the literature.[15]

Cu-Catalyzed Asymmetric Reductive Coupling

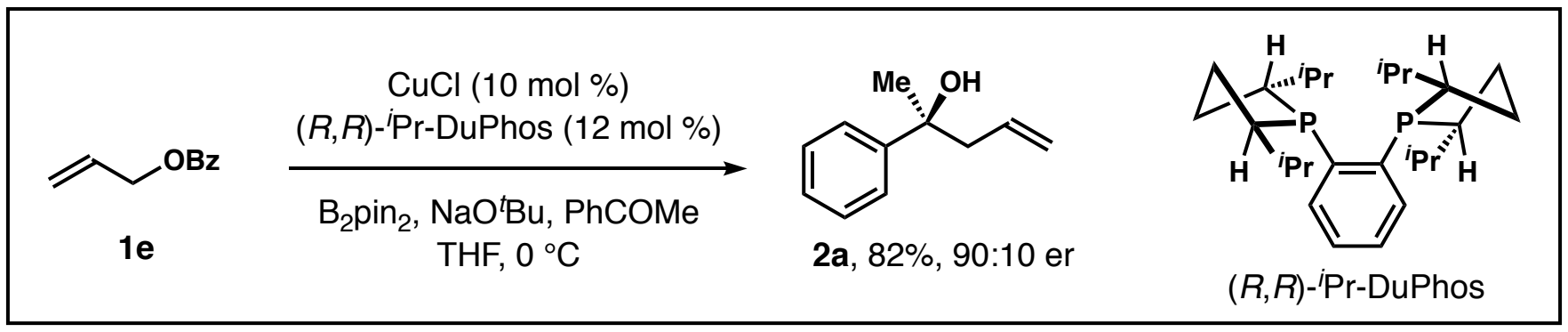

In a glovebox, an oven-dried 1-dram vial equipped with a magnetic stir bar was charged with CuCl (1 mg, 0.01 mmol, 10 mol %), (*R*,*R*)-^{i}Pr-DuPhos (5 mg, 0.012 mmol, 12 mol %), NaO^{t}Bu (19 mg, 0.2 mmol, 2.0 equiv), and THF (0.4 mL) under an argon atmosphere. After stirring for 10 minutes at ambient temperature, B_2pin_2 (56 mg, 0.22 mmol, 2.2 equiv) was added, and the reaction mixture was stirred for 10 minutes. Allylic benzoate **1e** (65 mg, 0.2 mmol, 2.0 equiv) was then added to the reaction mixture, followed by the addition of acetophenone (0.1 mmol, 1.0 equiv) at 0 °C. The resulting mixture was stirred for 24 h at 0 °C. Upon completion, ethyl acetate (2.0 mL) and water (2.0 mL) were added. The organic layer was separated, and the aqueous phase was extracted with ethyl acetate (2 × 2.0 mL). The combined organic extracts were filtered through a short pad of silica gel and then concentrated under reduced pressure. The crude product was purified by flash column chromatography (gradient elution with hexanes and ethyl acetate) to give product **2a** in 82% yield. Enantiomeric excess of **2a** was determined to be 80% ee (254 nm, 25 °C); t_1 = 5.16 min, t_2 = 5.45 min [hexane/i-PrOH, 95:5, 1.0 mL/min, (Chiralpak ID)]; $[\alpha]_D^{20}$ = −42.8 (c 1.99, $CHCl_3$). The absolute configuration of **2a** was determined by comparing the optical rotation to the literature data.[15-17]

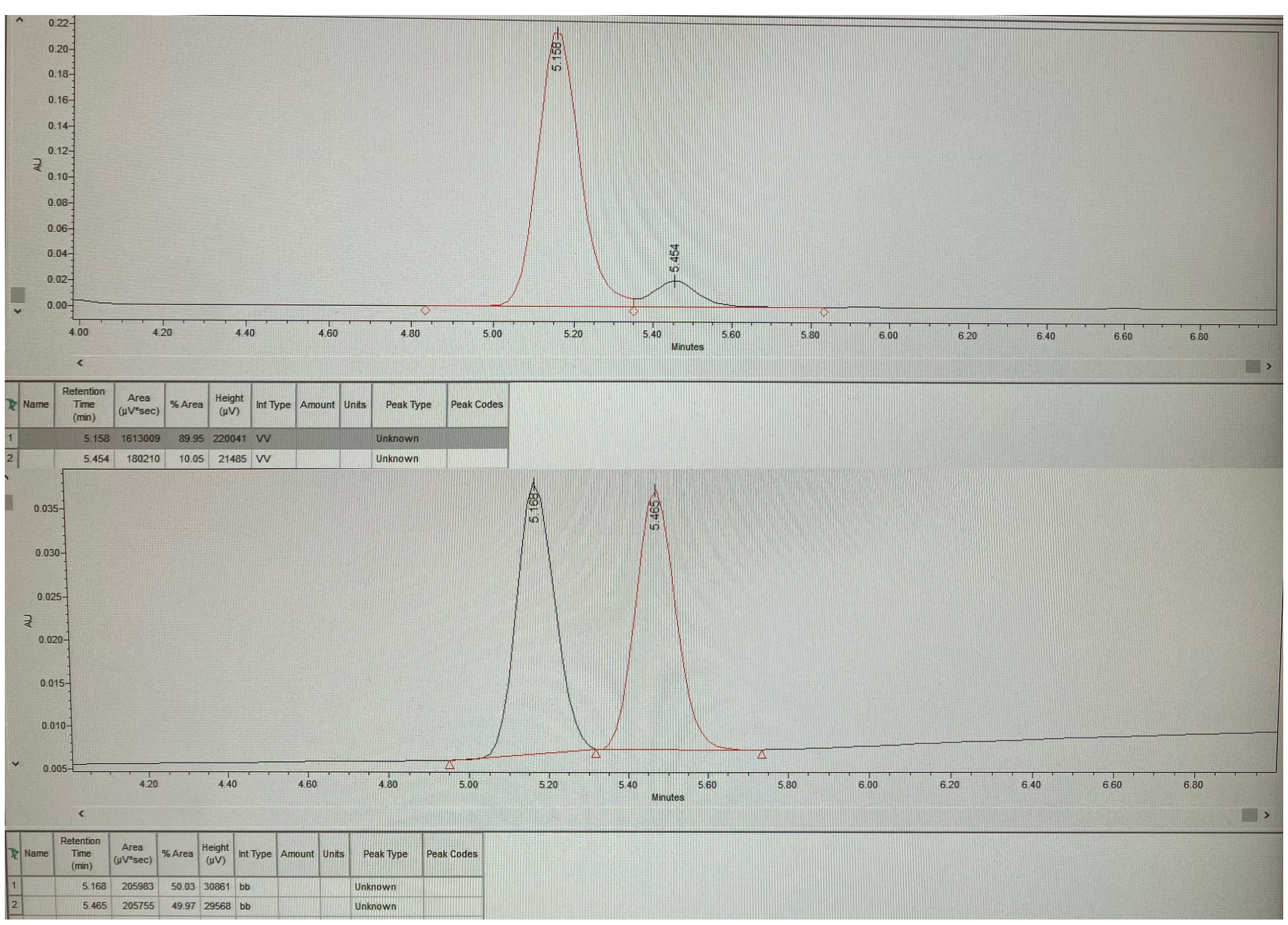

References:

1. Sawadjoon, S.; Sjöberg, P. J. R.; Orthaber, A.; Matsson, O.; Samec, J. S. M. *Chem. Eur. J.* **2014**, *20*, 1520.
2. Datilus, V.; AbdulKarim, M.; Patrizio, A.; Kaur, P. *Tetrahedron Lett.* **2016**, *57*, 2778.
3. Robbins, D. W.; Lee, K.; Silverio, D. L.; Volkov, A.; Torker, S.; Hoveyda, A. H. *Angew. Chem. Int. Ed.* **2016**, *55*, 9610.
4. Cunningham, A.; Mokal-Parekh, V.; Wilson, C.; Woodward, S. *Org. Biomol. Chem.* **2004**, *2*, 741.
5. Chatterjee, R.; Samanta, S.; Mukherjee, A.; Santra, S.; Zyryanov, G. V.; Majee, A. *Tetrahedron Lett*. **2019**, *60*, 276.
6. Schneider, U.; Ueno, M.; Kobayashi, S. *J. Am. Chem. Soc.* **2008**, *130*, 13824.
7. Schneider, U.; Kobayashi, S. *Angew. Chem., Int. Ed.* **2007**, *46*, 5909.
8. Jones, P.; Knochel, P. *J. Org. Chem.* **1999**, *64*, 186.
9. Denmark, S. E.; Nguyen, S. T. *Org. Lett.* **2009**, *11*, 781.
10. Li, S.; Wang, J.-X.; Wen, X.; Ma, X. *Tetrahedron* **2011**, *67*, 849.
11. Fernandes, R. A.; Yadav, S. S.; Kumar, P. *Org. Biomol. Chem.* **2022**, *20*, 427.
12. Aggarwal, V. K.; Vennall, G. P. *Synthesis* **1988**, 1822.
13. Matsuya, Y.; Sasaki, K.; Nagaoka, M.; Kakuda, H.; Toyooka, N.; Imanishi, N.; Ochiai, H.; Nemoto, H. *J. Org. Chem.* **2004**, *69*, 7989.
14. Volla, C. M. R.; Marković, D.; Laclef, S.; Vogel, P. *Chem. Eur. J.* **2010**, *16*, 8984.
15. Wada, R.; Oisaki, K,; Kanai, M.; Shibasaki, M. *J. Am. Chem. Soc.* **2004**, 126, 8910.
16. Yamasaki, S.; Fujii, K.; Wada, R.; Kanai, M.; Shibasaki, M. *J. Am. Chem. Soc.* **2002**, *124*, 6536.
17. Shi, S.-L.; Xu, L.-X.; Oisaki, K.; Kanai, M.; Shibasaki, M. *J. Am. Chem. Soc.* **2010**, *132*, 6638.

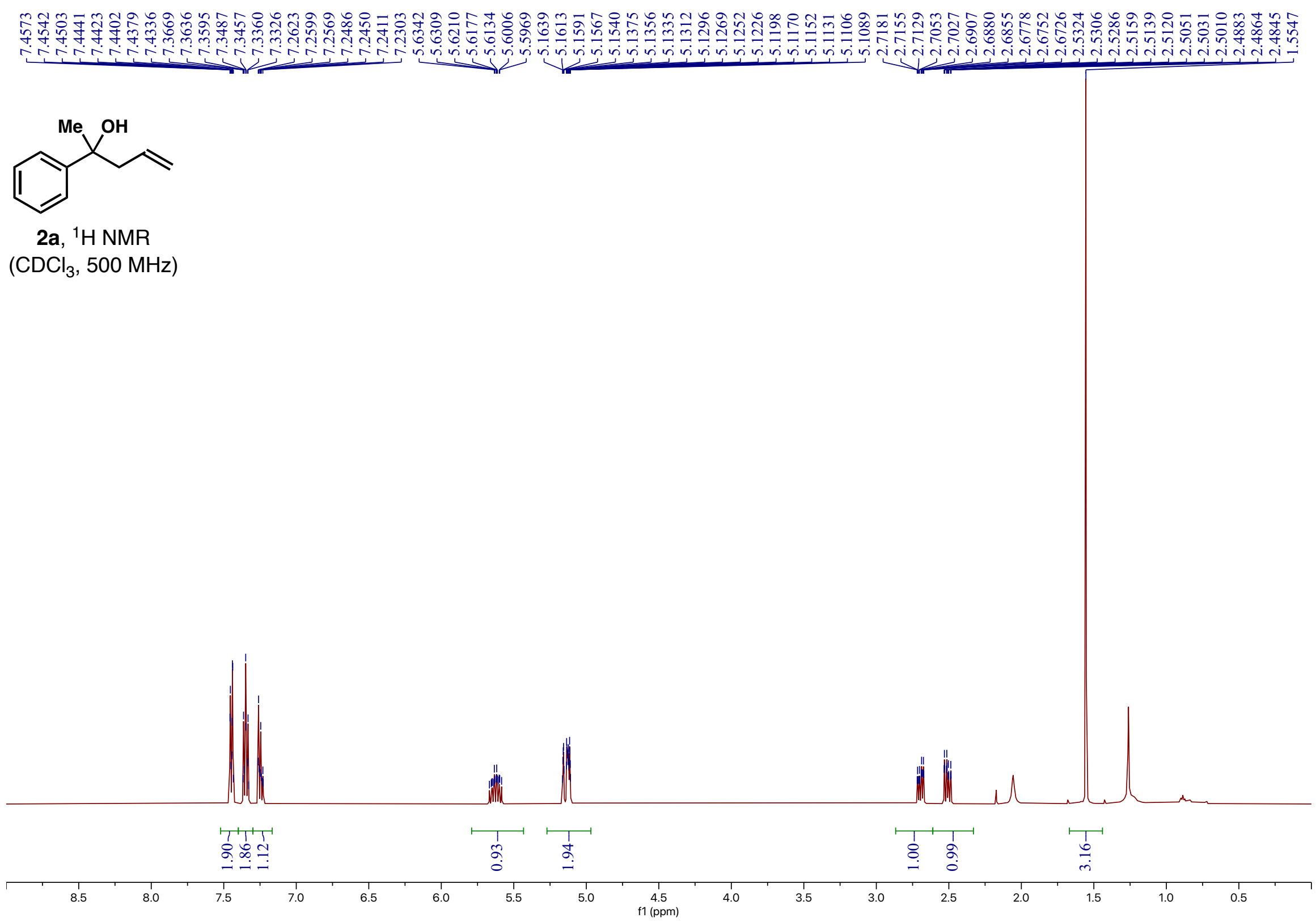

2a, ^{1}H NMR
($CDCl_3$, 500 MHz)

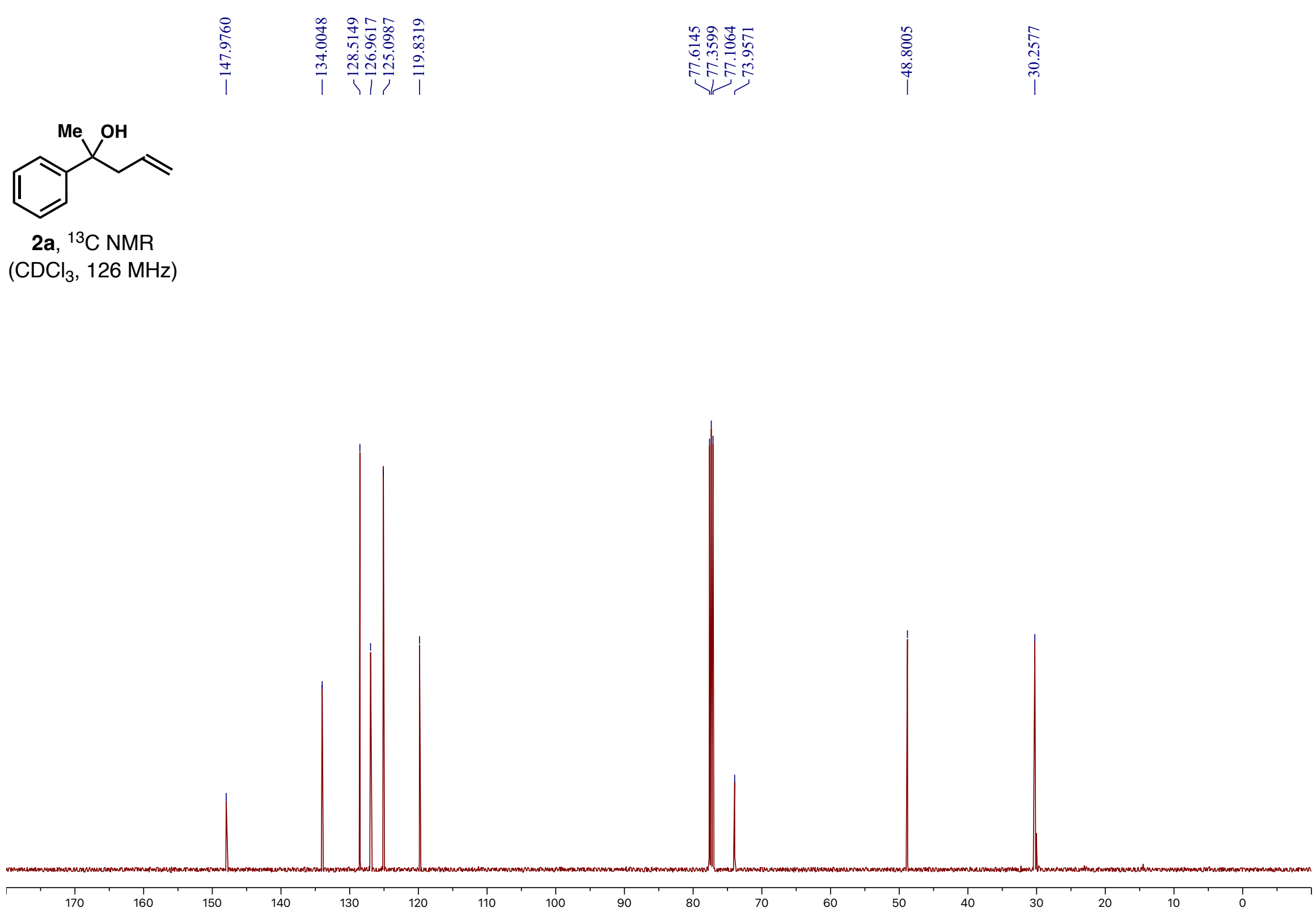

2a, ^{13}C NMR
($CDCl_3$, 126 MHz)

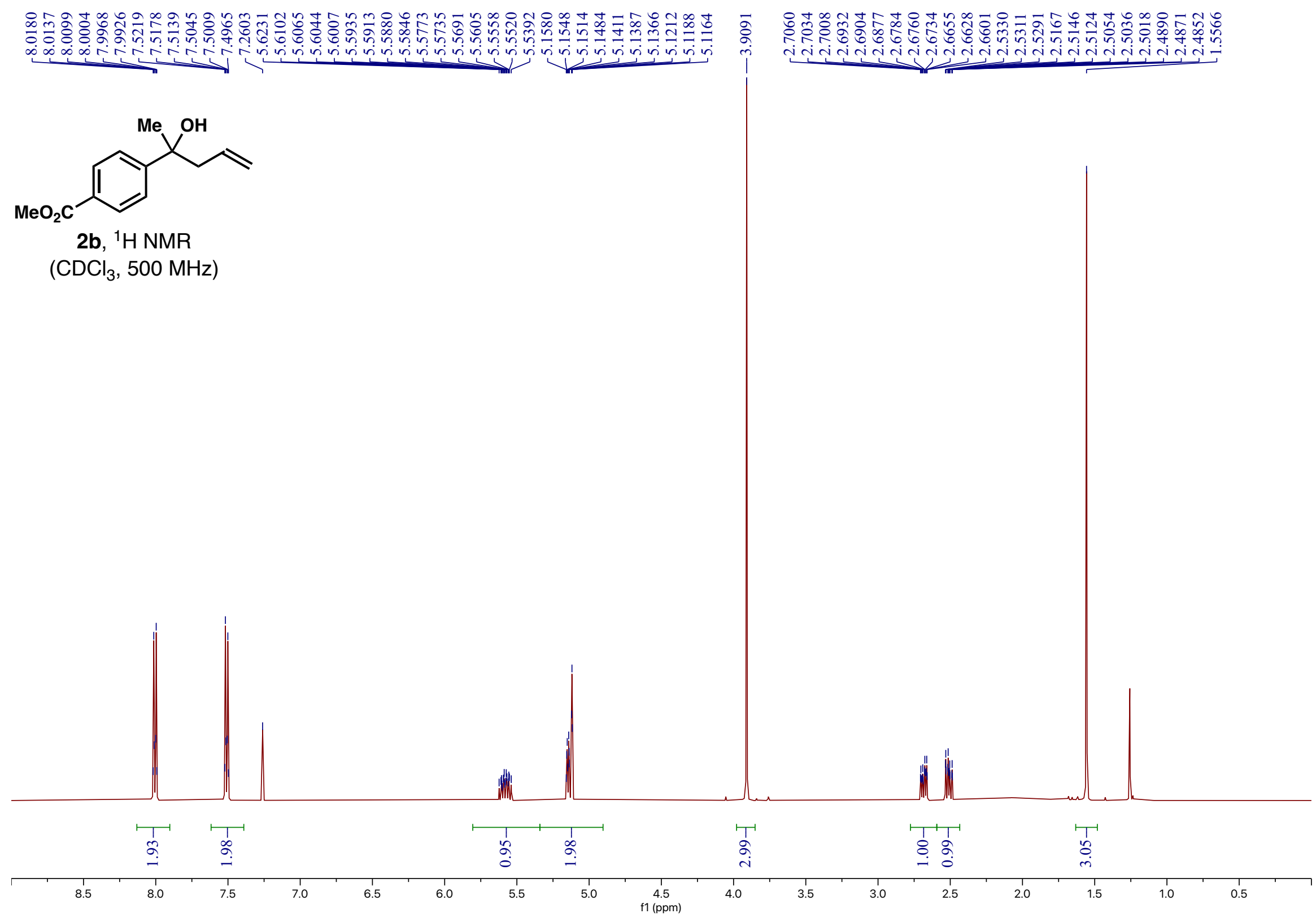
8.0180
8.0137
8.0099
8.0004
7.9968
7.9926
7.5219
7.5178
7.5139
7.5045
7.5009
7.4965
7.2603
5.6231
5.6102
5.6065
5.6044
5.6007
5.5935
5.5913
5.5880
5.5846
5.5773
5.5735
5.5691
5.5605
5.5558
5.5520
5.5392
5.1580
5.1548
5.1514
5.1484
5.1411
5.1387
5.1366
5.1212
5.1188
5.1164
3.9091
2.7060
2.7034
2.7008
2.6932
2.6904
2.6877
2.6784
2.6760
2.6734
2.6655
2.6628
2.6601
2.5330
2.5311
2.5291
2.5167
2.5146
2.5124
2.5054
2.5036
2.5018
2.4890
2.4871
2.4852
1.5566
Me
OH
MeO2C
2b, 1H NMR
(CDCl3, 500 MHz)
1.93
1.98
0.95
1.98
2.99
1.00
0.99
3.05
8.5
8.0
7.5
7.0
6.5
6.0
5.5
5.0
4.5
4.0
3.5
3.0
2.5
2.0
1.5
1.0
0.5
f1 (ppm)

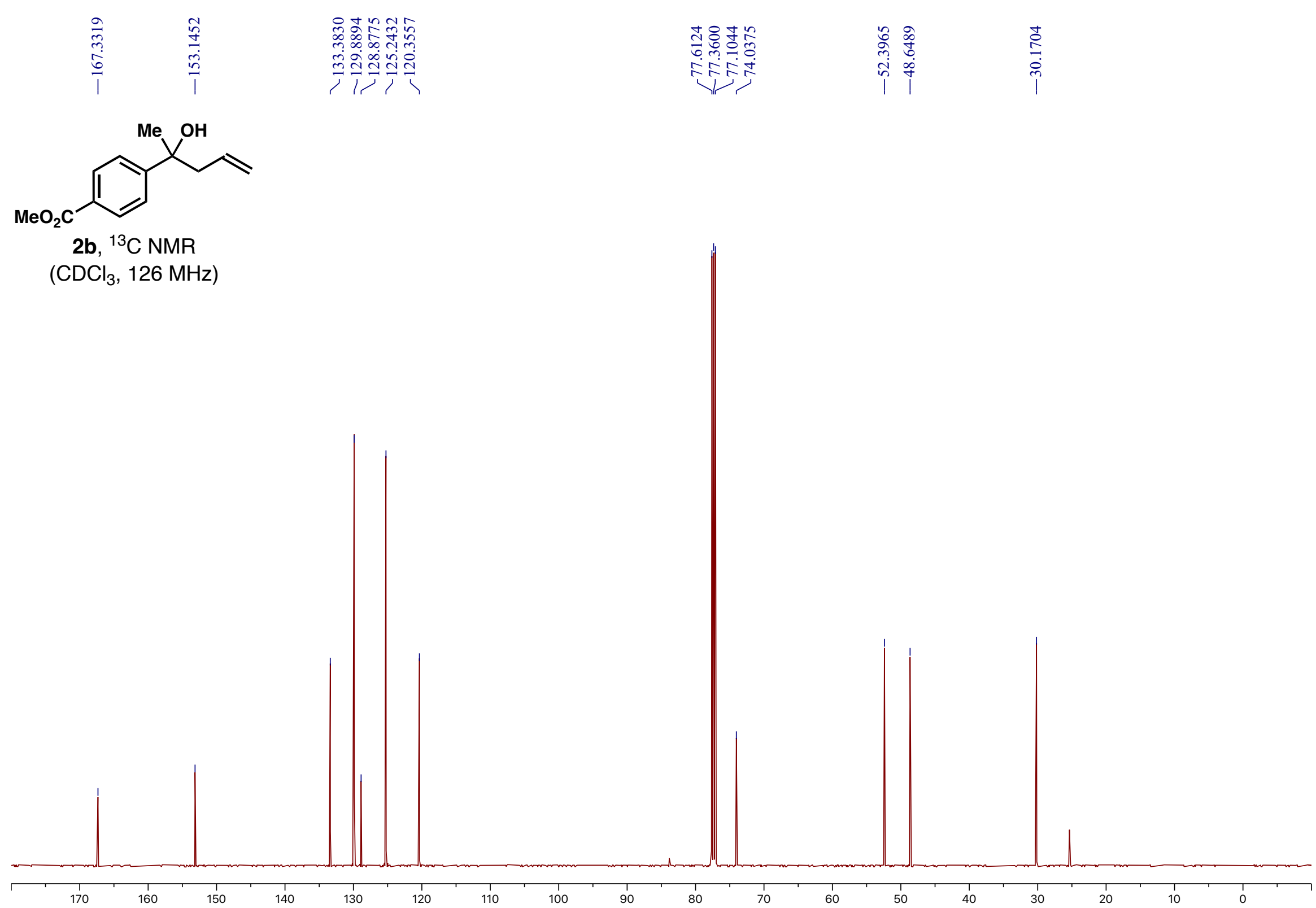
167.3319
153.1452
133.3830
129.8894
128.8775
125.2432
120.3557
77.6124
77.3600
77.1044
74.0375
52.3965
48.6489
30.1704
Me
OH
MeO2C
2b, 13C NMR
(CDCl3, 126 MHz)
170
160
150
140
130
120
110
100
90
80
70
60
50
40
30
20
10
0
f1 (ppm)

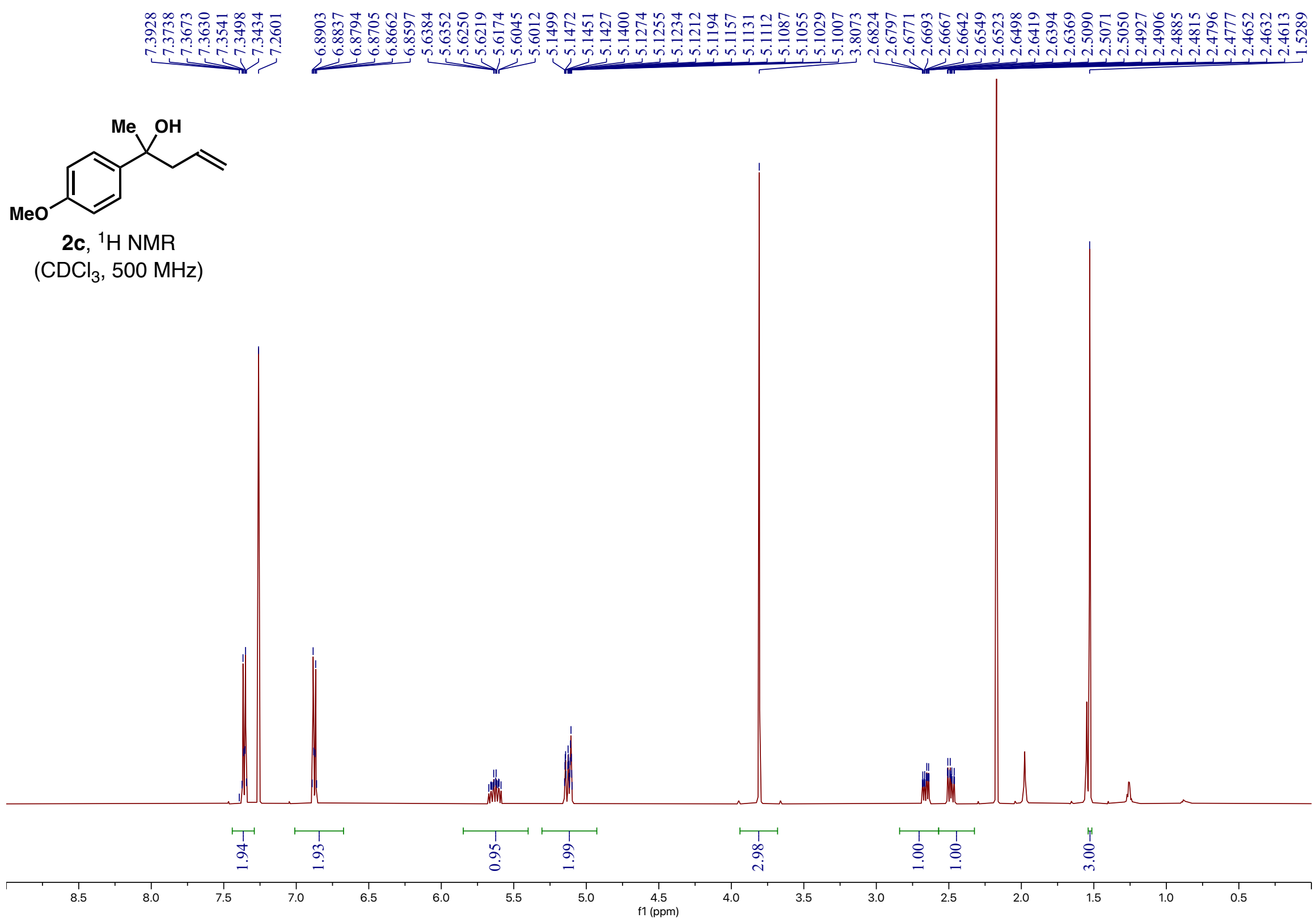
Me
OH
MeO
2c, 1H NMR
(CDCl3, 500 MHz)
f1 (ppm)

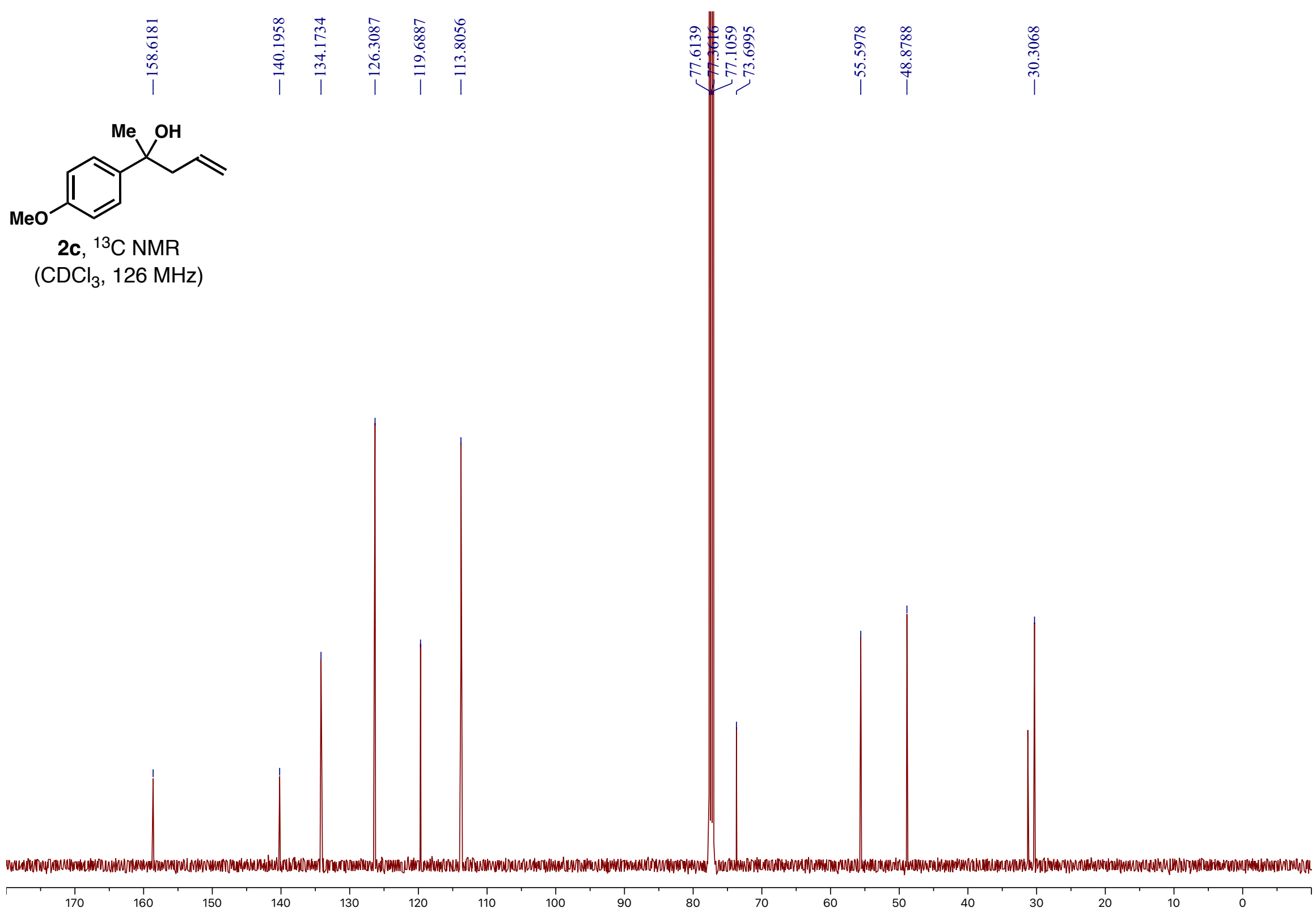
Me
OH
MeO
2c, 13C NMR
(CDCl3, 126 MHz)
f1 (ppm)

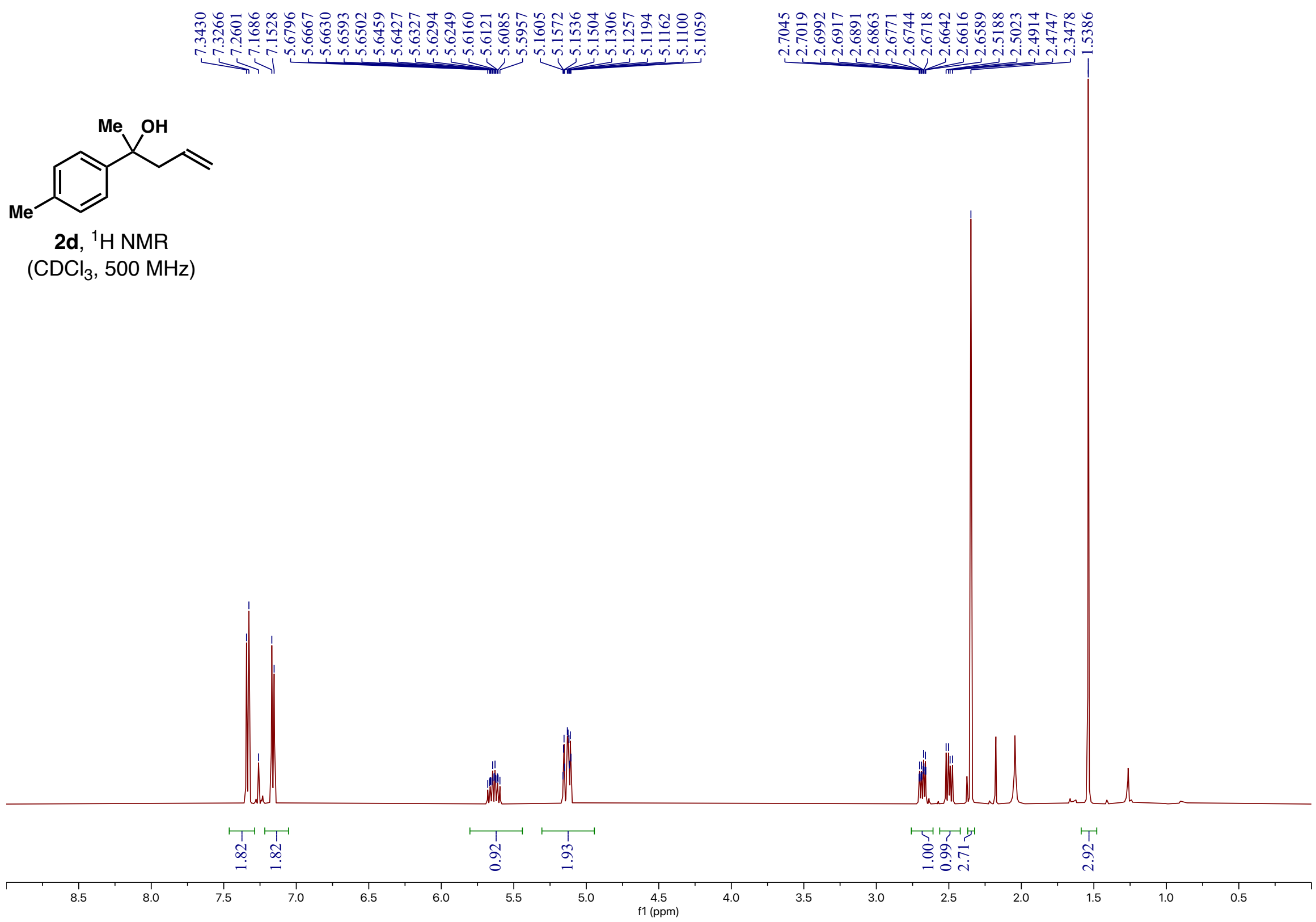
7.3430
7.3266
7.2601
7.1686
7.1528
5.6796
5.6667
5.6630
5.6593
5.6502
5.6459
5.6427
5.6327
5.6294
5.6249
5.6160
5.6121
5.6085
5.5957
5.1605
5.1572
5.1536
5.1504
5.1306
5.1257
5.1194
5.1162
5.1100
5.1059
2.7045
2.7019
2.6992
2.6917
2.6891
2.6863
2.6771
2.6744
2.6718
2.6642
2.6616
2.6589
2.5188
2.5023
2.4914
2.4747
2.3478
1.5386
Me
OH
Me
2d, ^{1}H NMR
($CDCl_3$, 500 MHz)
1.82
1.82
0.92
1.93
1.00
0.99
2.71
2.92
f1 (ppm)

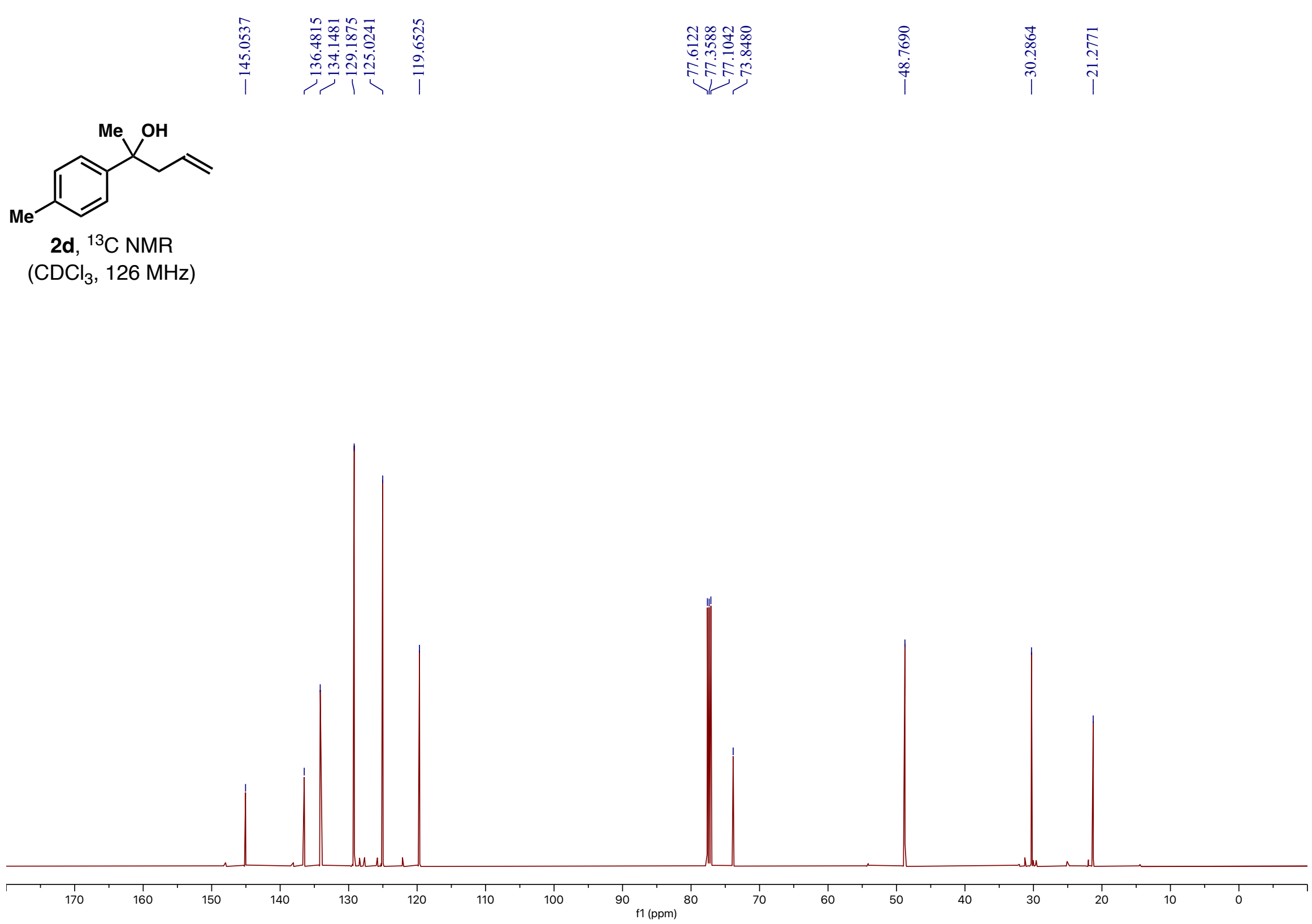
145.0537
136.4815
134.1481
129.1875
125.0241
119.6525
77.6122
77.3588
77.1042
73.8480
48.7690
30.2864
21.2771
Me
OH
Me
2d, ^{13}C NMR
($CDCl_3$, 126 MHz)
f1 (ppm)

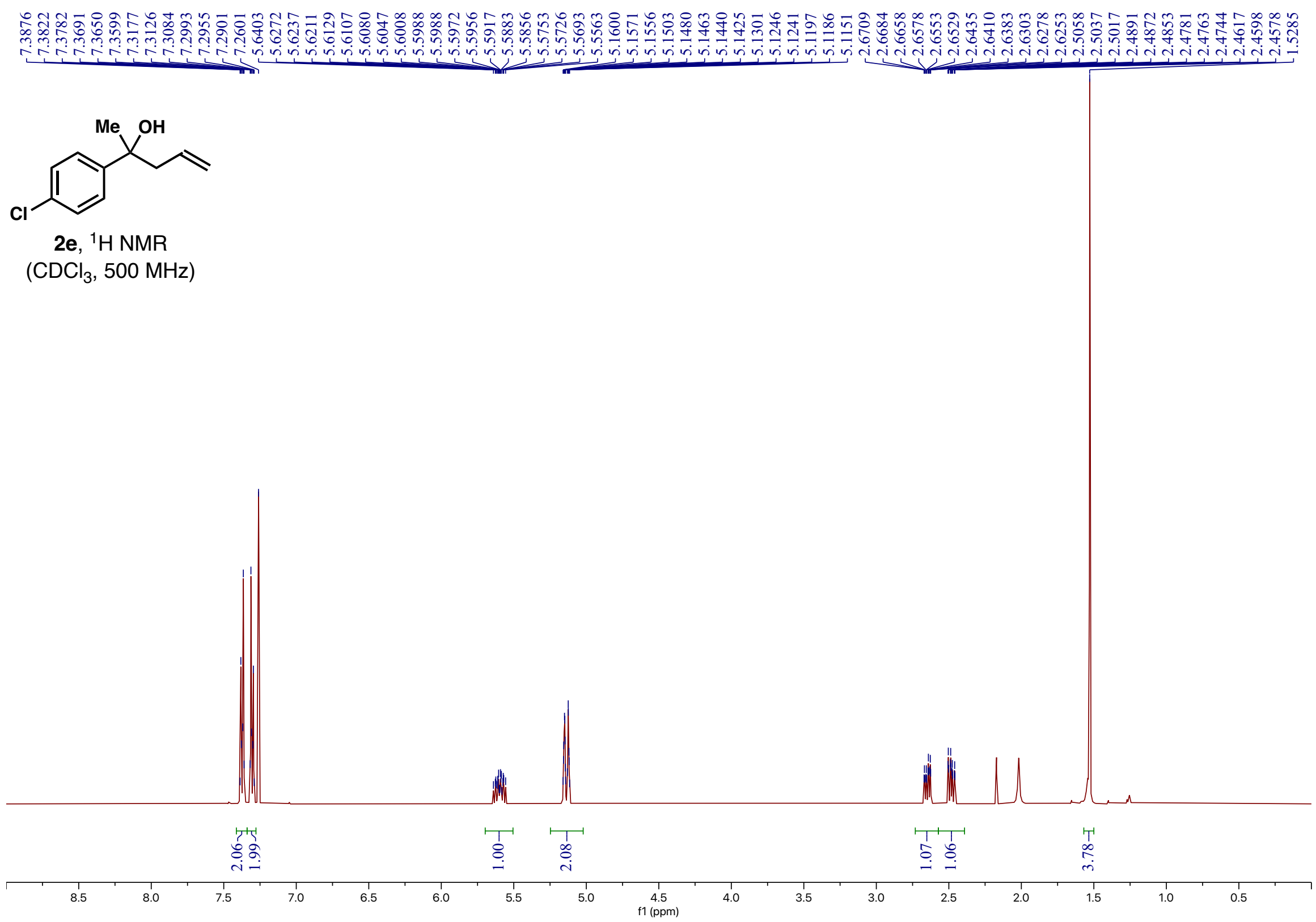
7.3876
7.3822
7.3782
7.3691
7.3650
7.3599
7.3177
7.3126
7.3084
7.2993
7.2955
7.2901
7.2601
5.6403
5.6272
5.6237
5.6211
5.6129
5.6107
5.6080
5.6047
5.6008
5.5988
5.5988
5.5972
5.5956
5.5917
5.5883
5.5856
5.5753
5.5726
5.5693
5.5563
5.1600
5.1571
5.1556
5.1503
5.1480
5.1463
5.1440
5.1425
5.1301
5.1246
5.1241
5.1197
5.1186
5.1151
2.6709
2.6684
2.6658
2.6578
2.6553
2.6529
2.6435
2.6410
2.6383
2.6303
2.6278
2.6253
2.5058
2.5037
2.5017
2.4891
2.4872
2.4853
2.4781
2.4763
2.4744
2.4617
2.4598
2.4578
1.5285
Me
OH
Cl
2e, 1H NMR
(CDCl3, 500 MHz)
2.06
1.99
1.00
2.08
1.07
1.06
3.78
f1 (ppm)

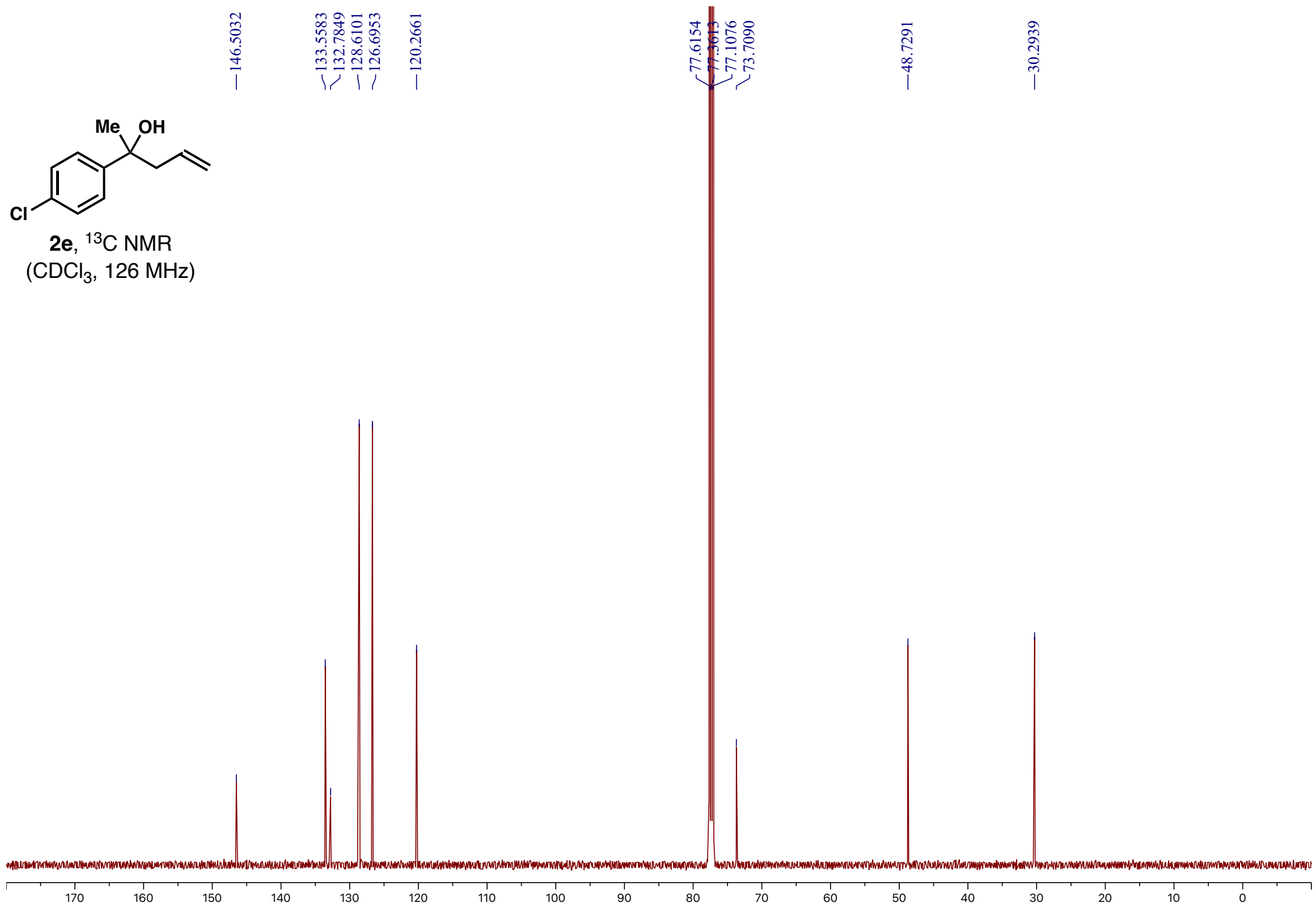
146.5032
133.5583
132.7849
128.6101
126.6953
120.2661
77.6154
77.3613
77.1076
73.7090
48.7291
30.2939
Me
OH
Cl
2e, 13C NMR
(CDCl3, 126 MHz)
f1 (ppm)

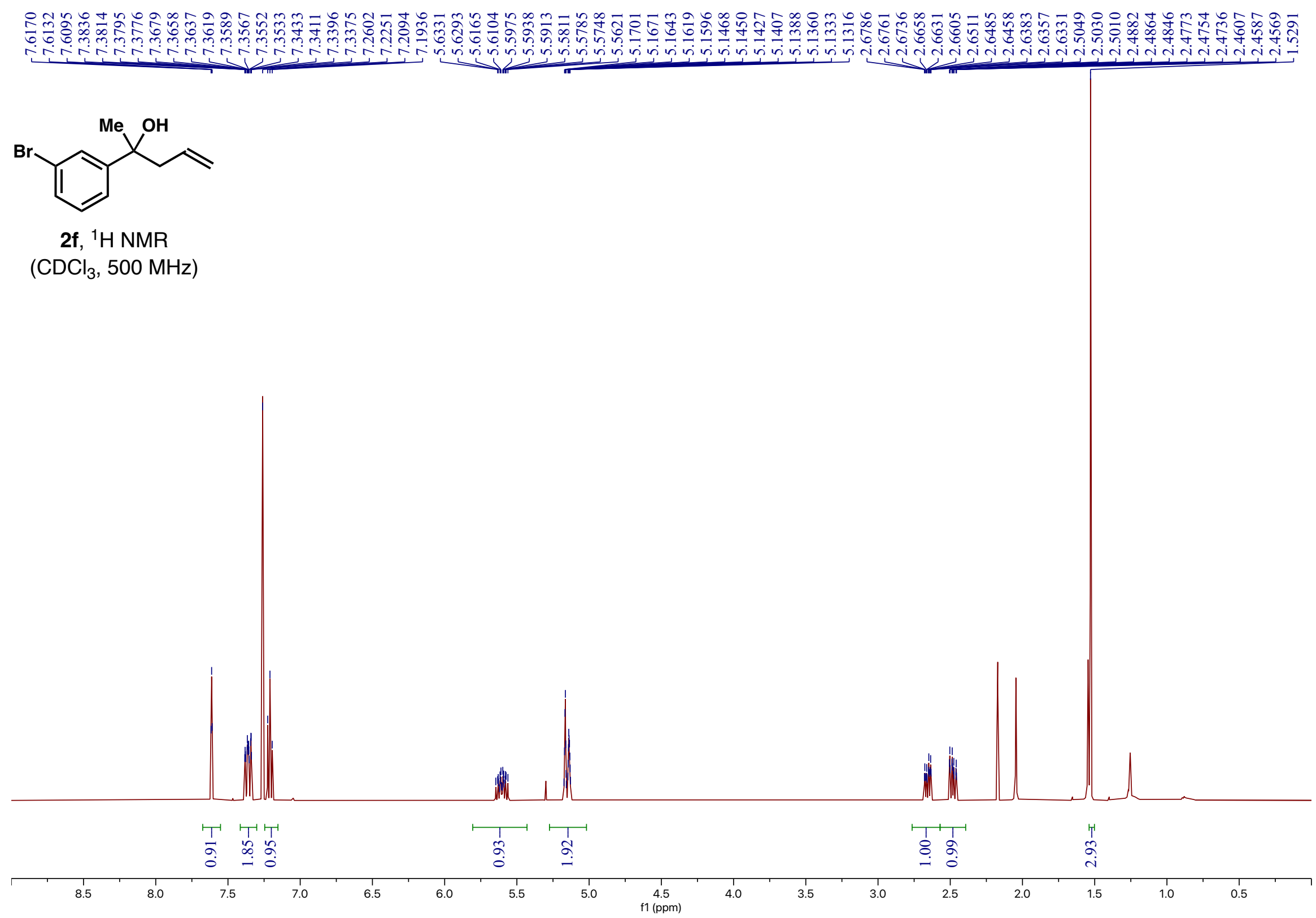
Me
OH
Br
2f, 1H NMR
(CDCl3, 500 MHz)
f1 (ppm)

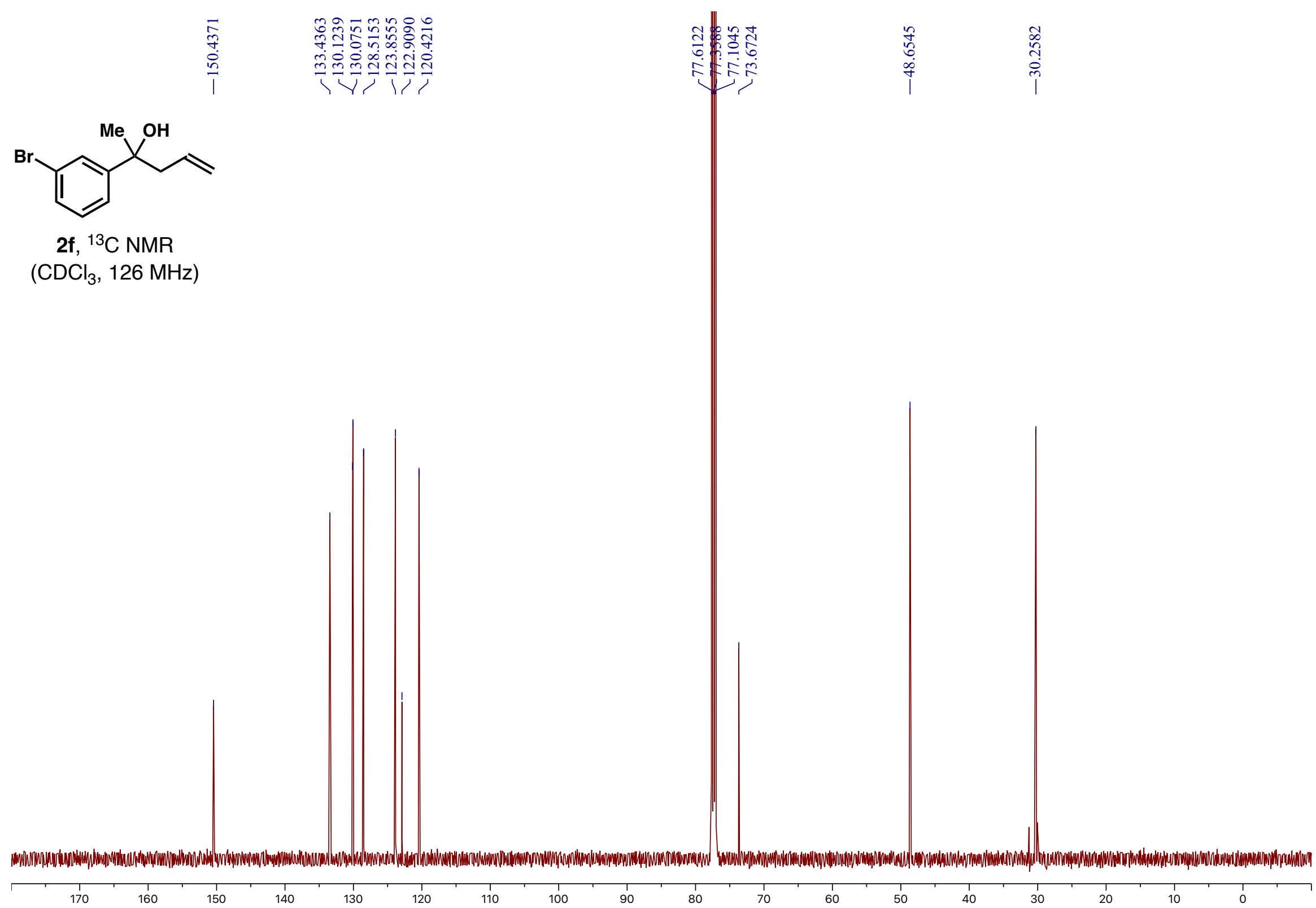
150.4371
133.4363
130.1239
130.0751
128.5153
123.8555
122.9090
120.4216
77.6122
77.3588
77.1045
73.6724
48.6545
30.2582
Me
OH
Br
2f, 13C NMR
(CDCl3, 126 MHz)
f1 (ppm)

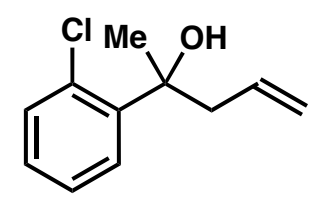

2g, ^{1}H NMR
($CDCl_3$, 500 MHz)

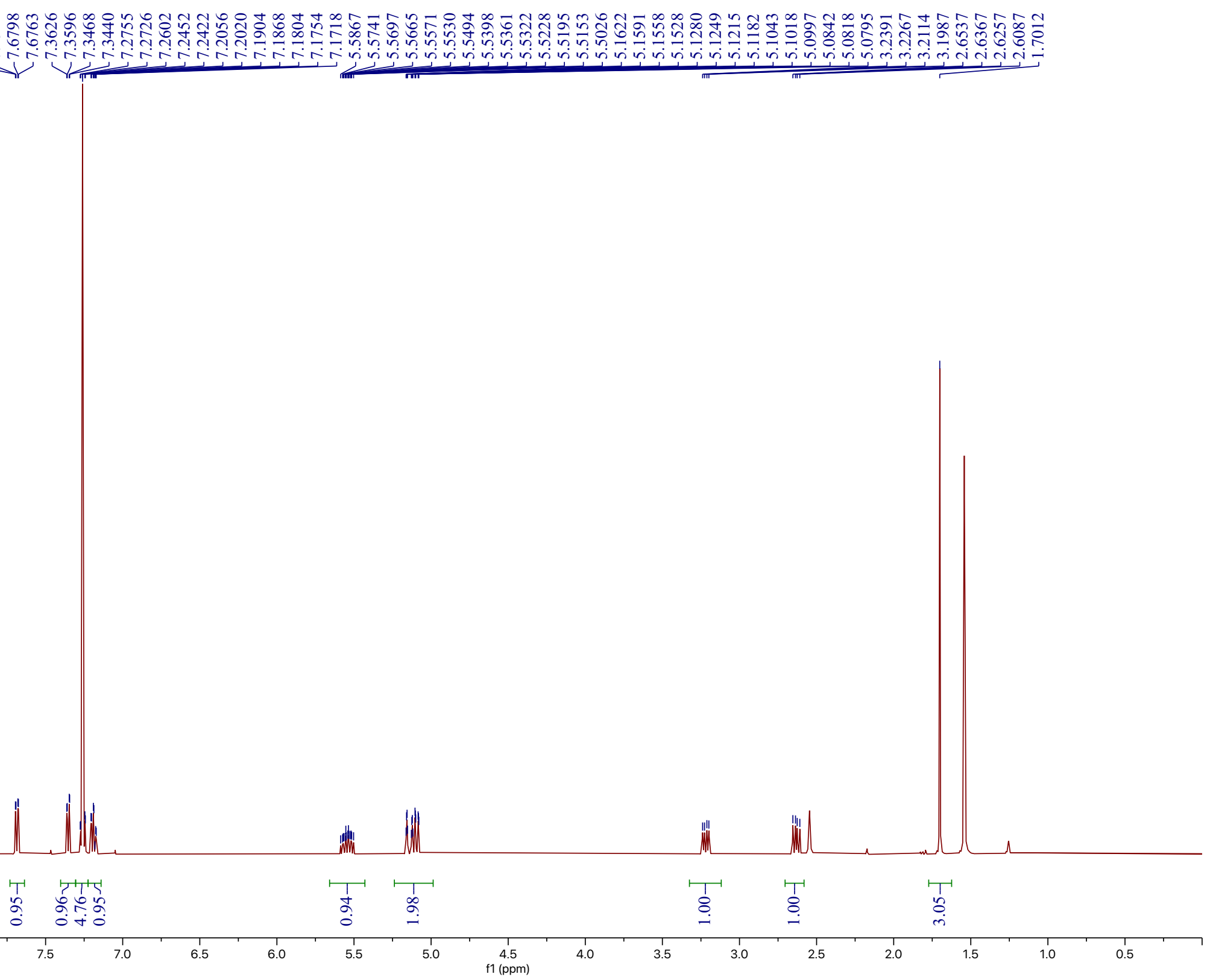

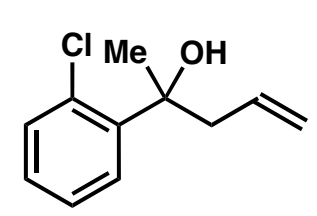

2g, ^{13}C NMR
($CDCl_3$, 126 MHz)

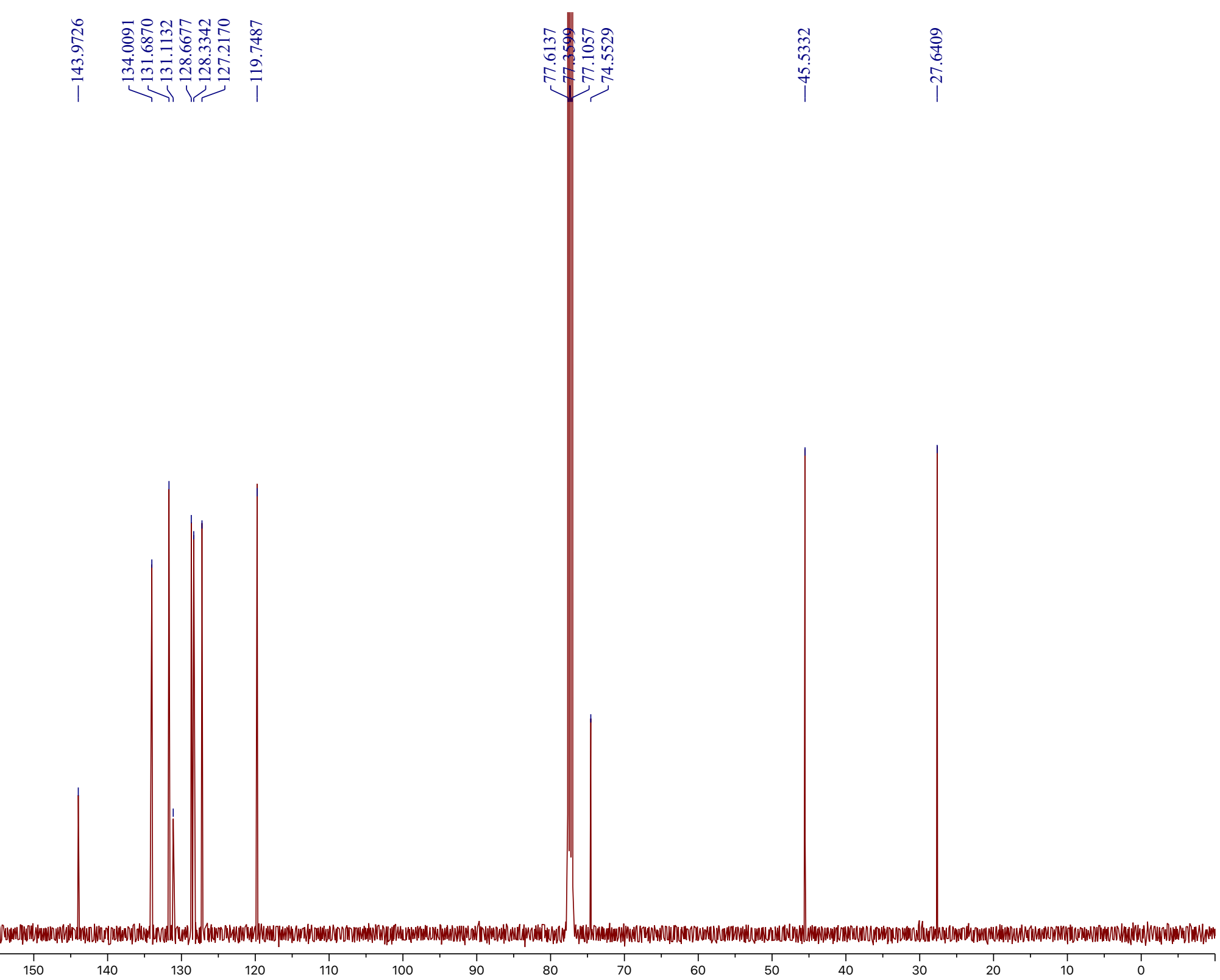

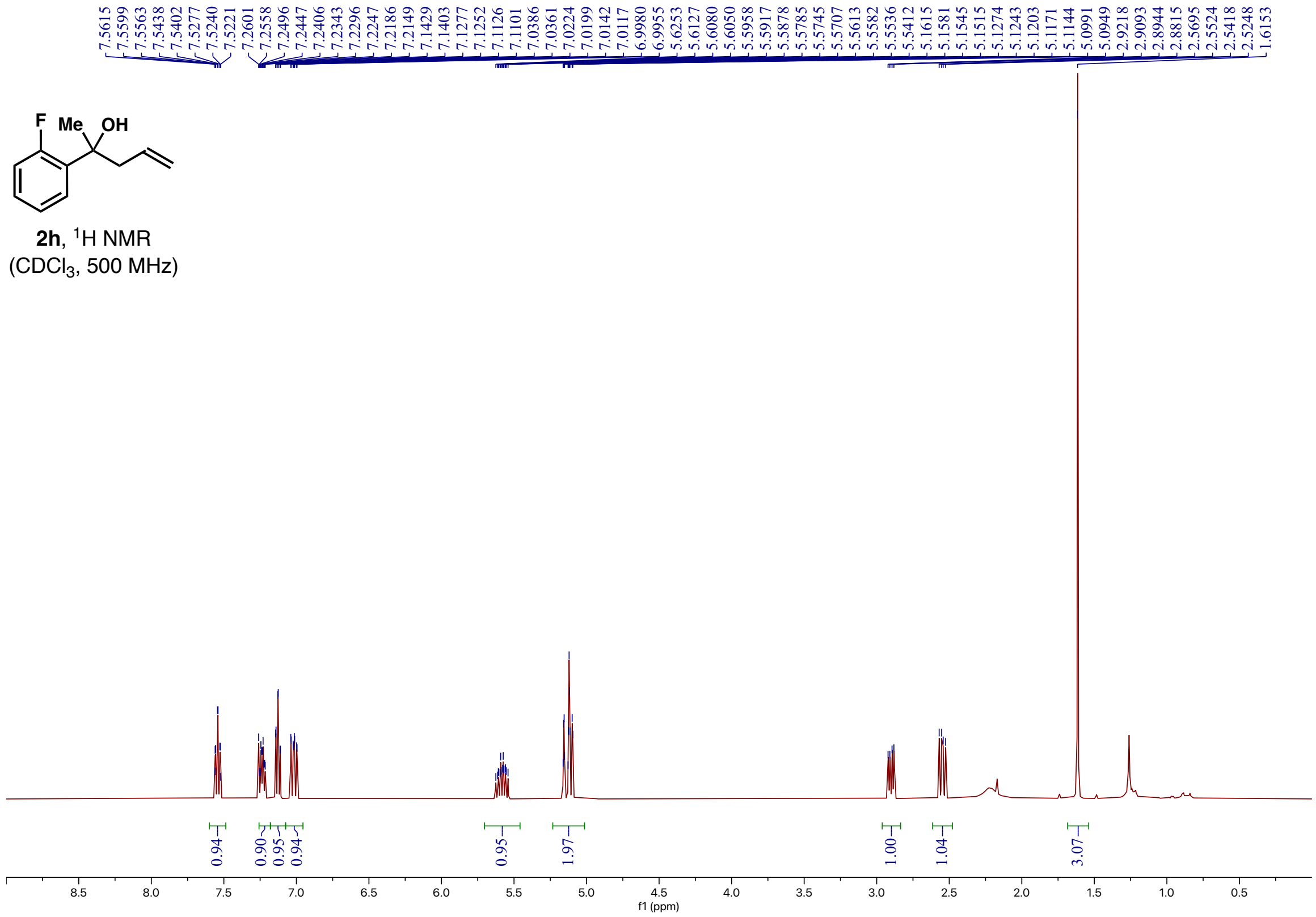

2h, ^{1}H NMR ($CDCl_3$, 500 MHz)

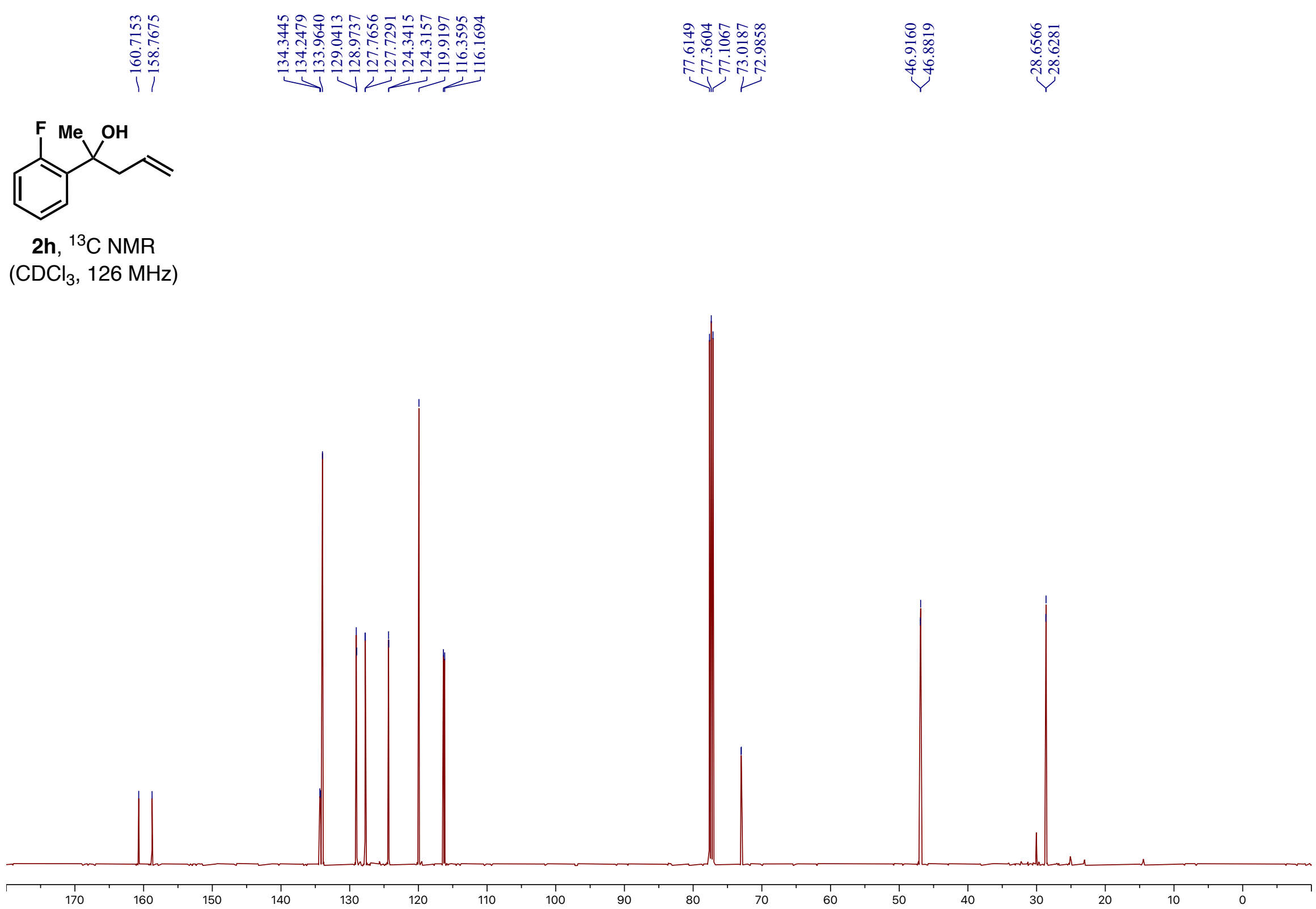

2h, ^{13}C NMR ($CDCl_3$, 126 MHz)

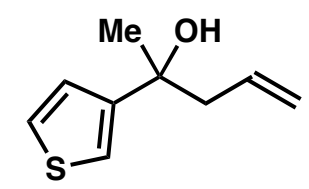

2i, ^{1}H NMR
($CDCl_3$, 500 MHz)

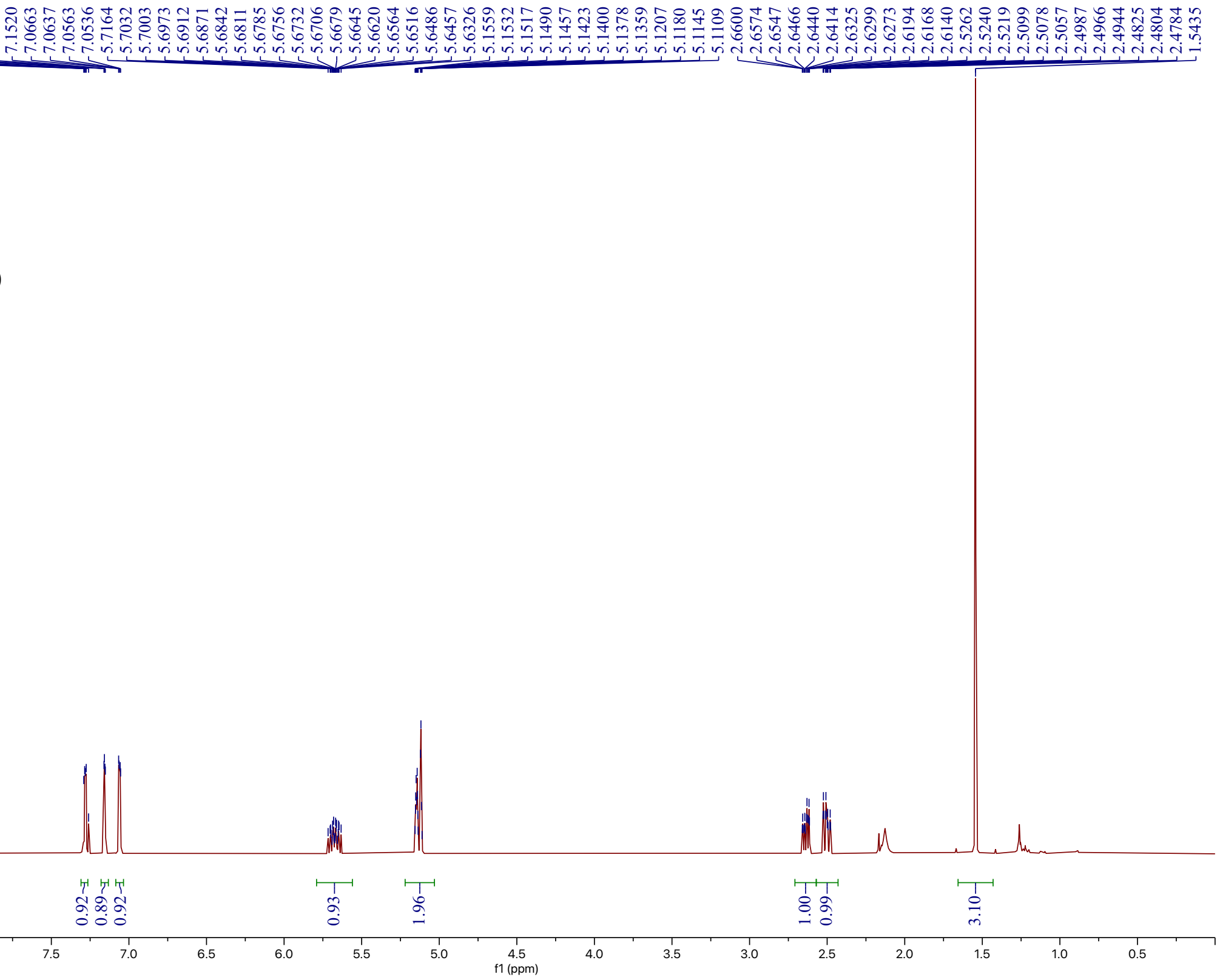

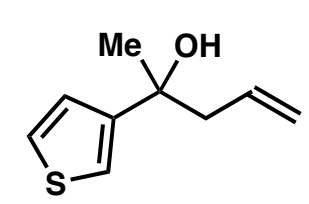

2i, ^{13}C NMR
($CDCl_3$, 126 MHz)

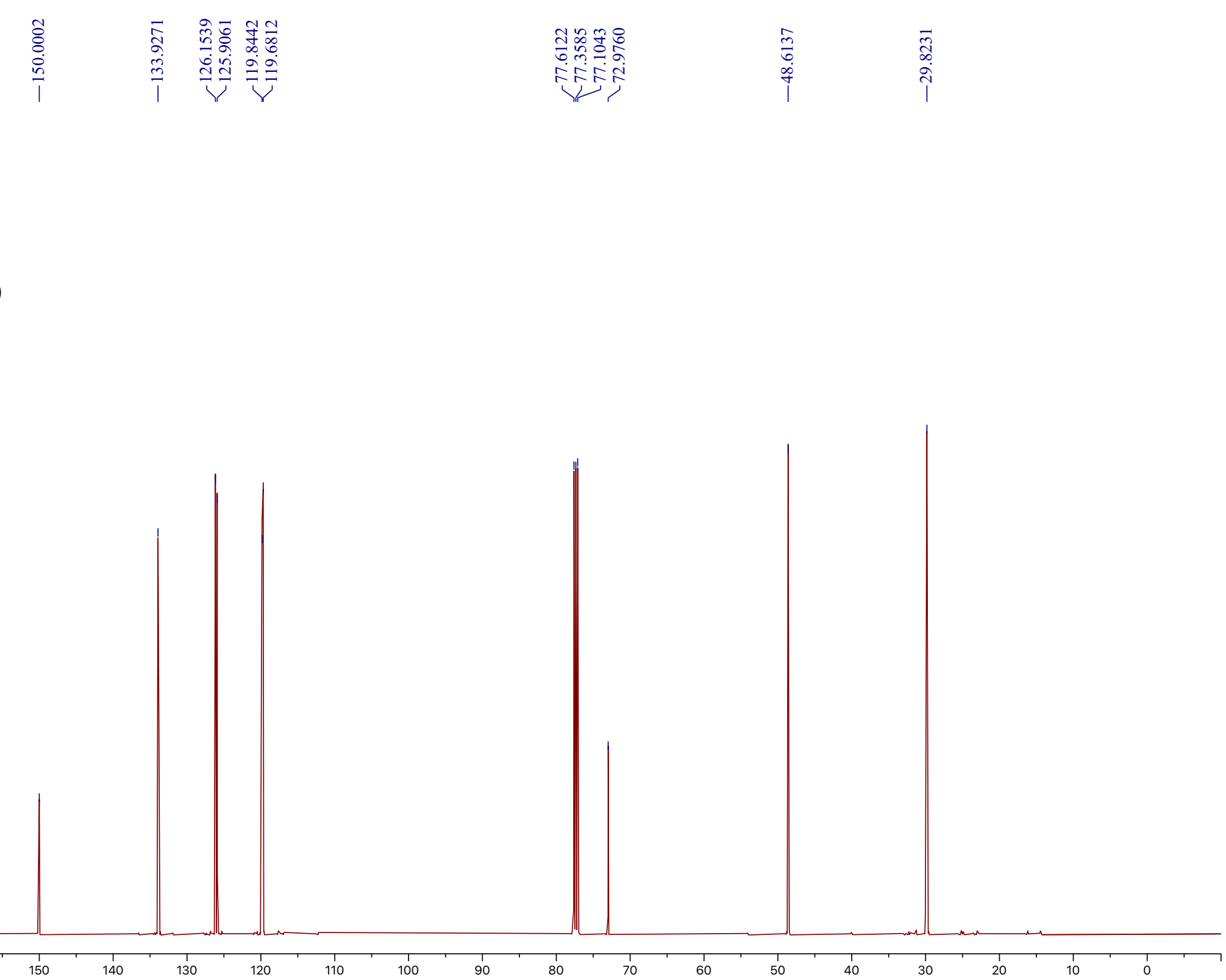

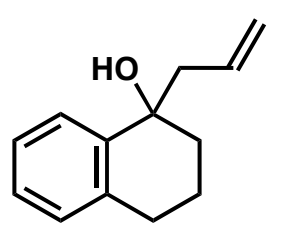

2j, ^{1}H NMR
($CDCl_3$, 500 MHz)

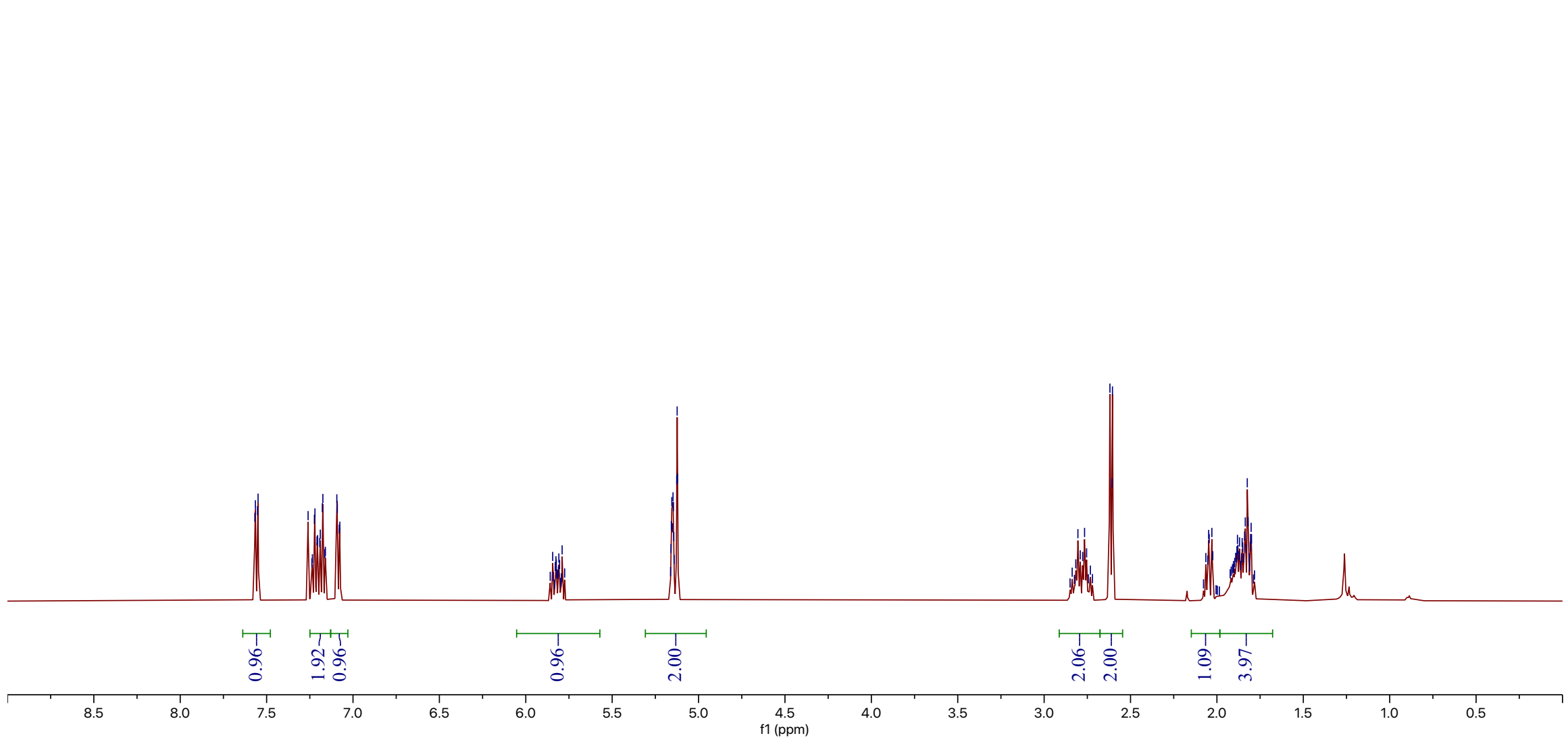

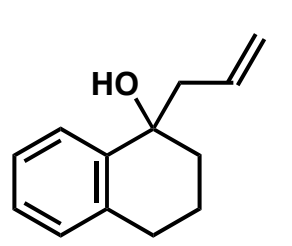

2j, ^{13}C NMR
($CDCl_3$, 126 MHz)

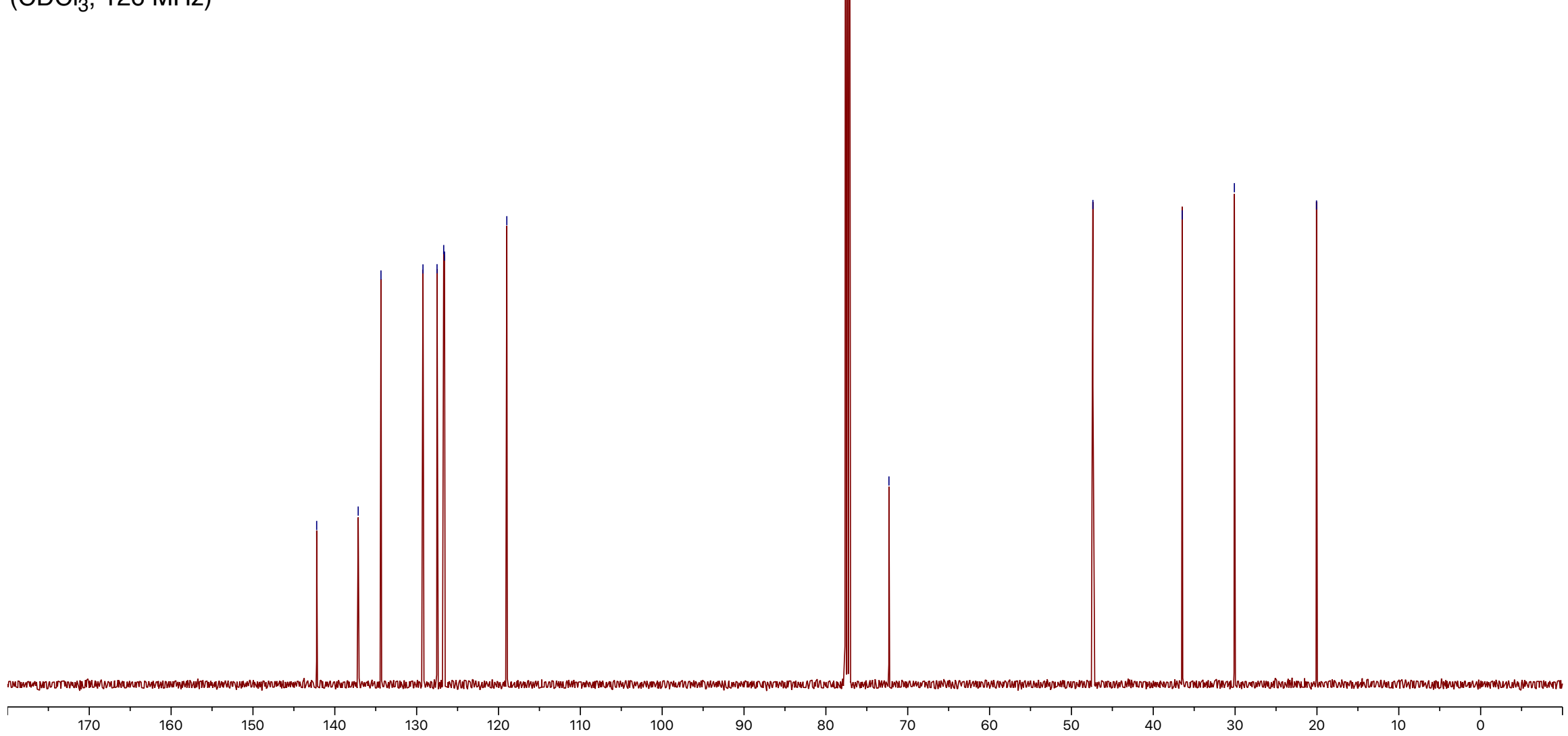

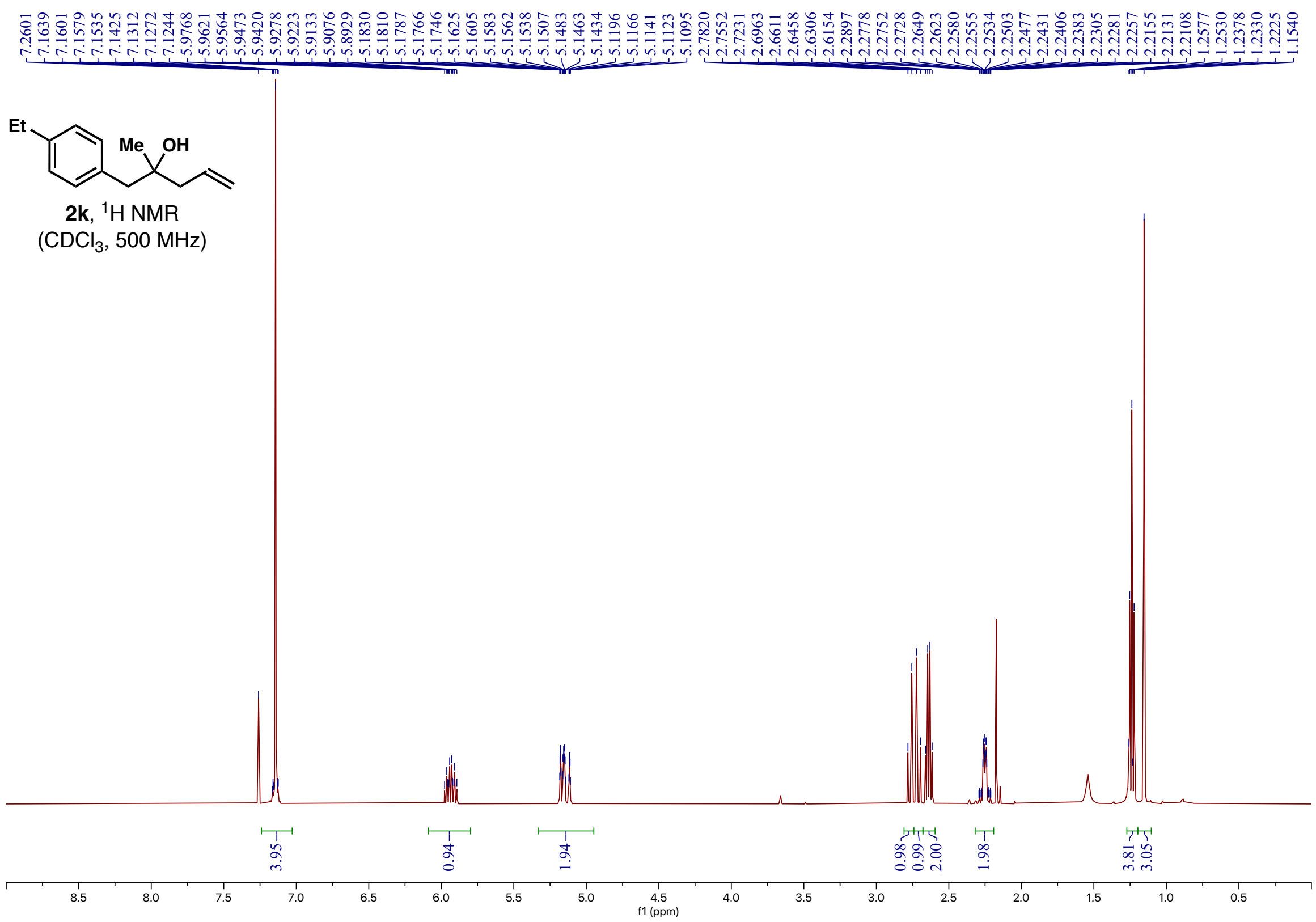
Et
Me
OH
2k, 1H NMR
(CDCl3, 500 MHz)
7.2601
7.1639
7.1601
7.1579
7.1535
7.1425
7.1312
7.1272
7.1244
5.9768
5.9621
5.9564
5.9473
5.9420
5.9278
5.9223
5.9133
5.9076
5.8929
5.1830
5.1810
5.1787
5.1766
5.1746
5.1625
5.1605
5.1583
5.1562
5.1538
5.1507
5.1483
5.1463
5.1434
5.1196
5.1166
5.1141
5.1123
5.1095
2.7820
2.7552
2.7231
2.6963
2.6611
2.6458
2.6306
2.6154
2.2897
2.2778
2.2752
2.2728
2.2649
2.2623
2.2580
2.2555
2.2534
2.2503
2.2477
2.2431
2.2406
2.2383
2.2305
2.2281
2.2257
2.2155
2.2131
2.2108
1.2577
1.2530
1.2378
1.2330
1.2225
1.1540
3.95
0.94
1.94
0.98
0.99
2.00
1.98
3.81
3.05
f1 (ppm)

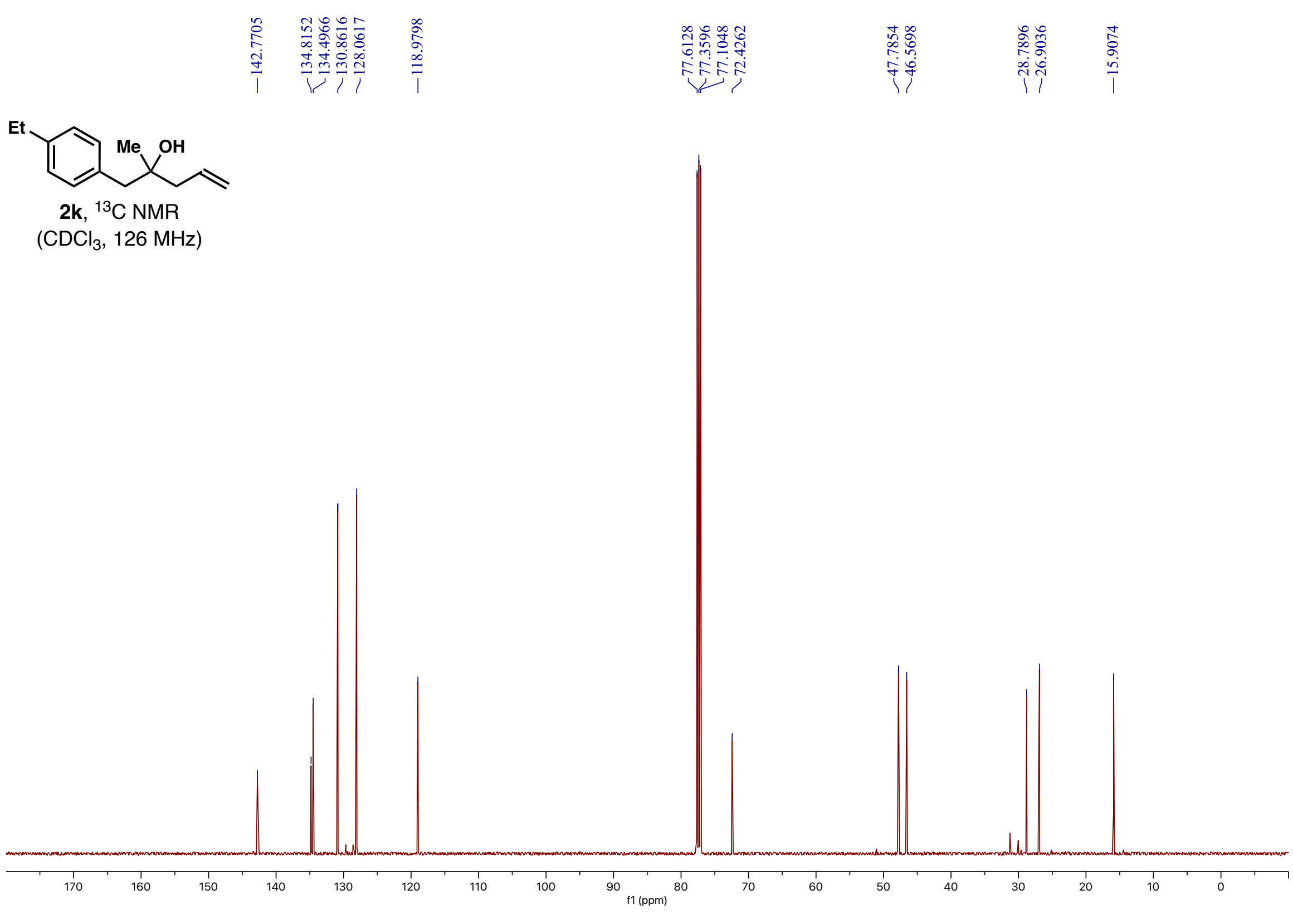
Et
Me
OH
2k, 13C NMR
(CDCl3, 126 MHz)
142.7705
134.8152
134.4966
130.8616
128.0617
118.9798
77.6128
77.3596
77.1048
72.4262
47.7854
46.5698
28.7896
26.9036
15.9074
f1 (ppm)

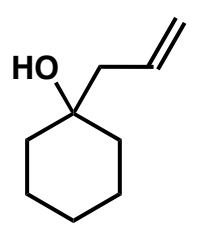

2l, ¹H NMR
($CDCl_3$, 500 MHz)

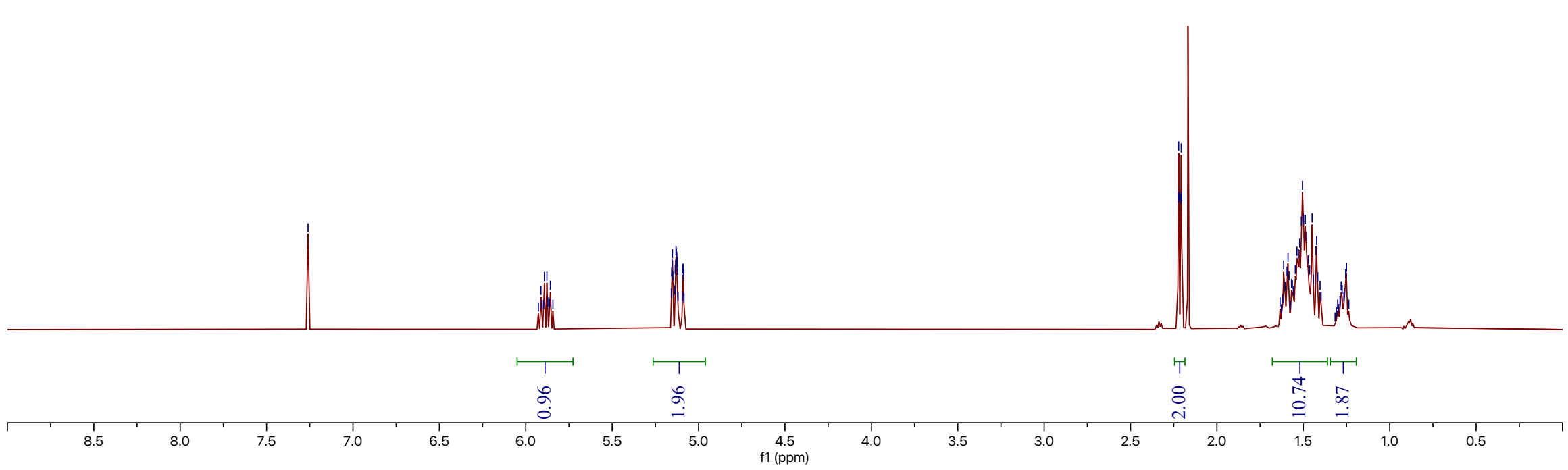

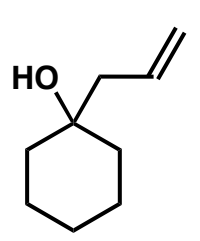

2l, ¹³C NMR
($CDCl_3$, 126 MHz)

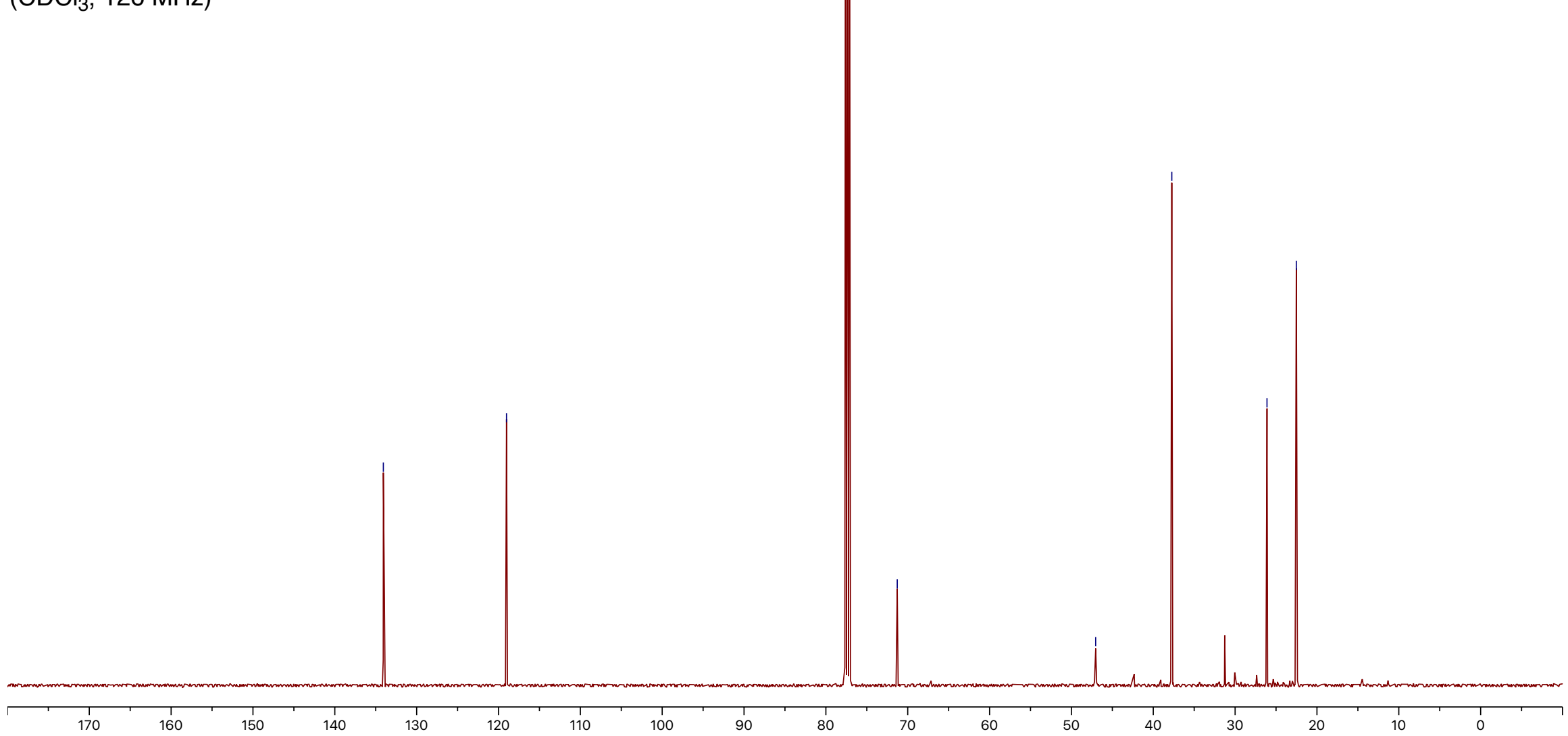

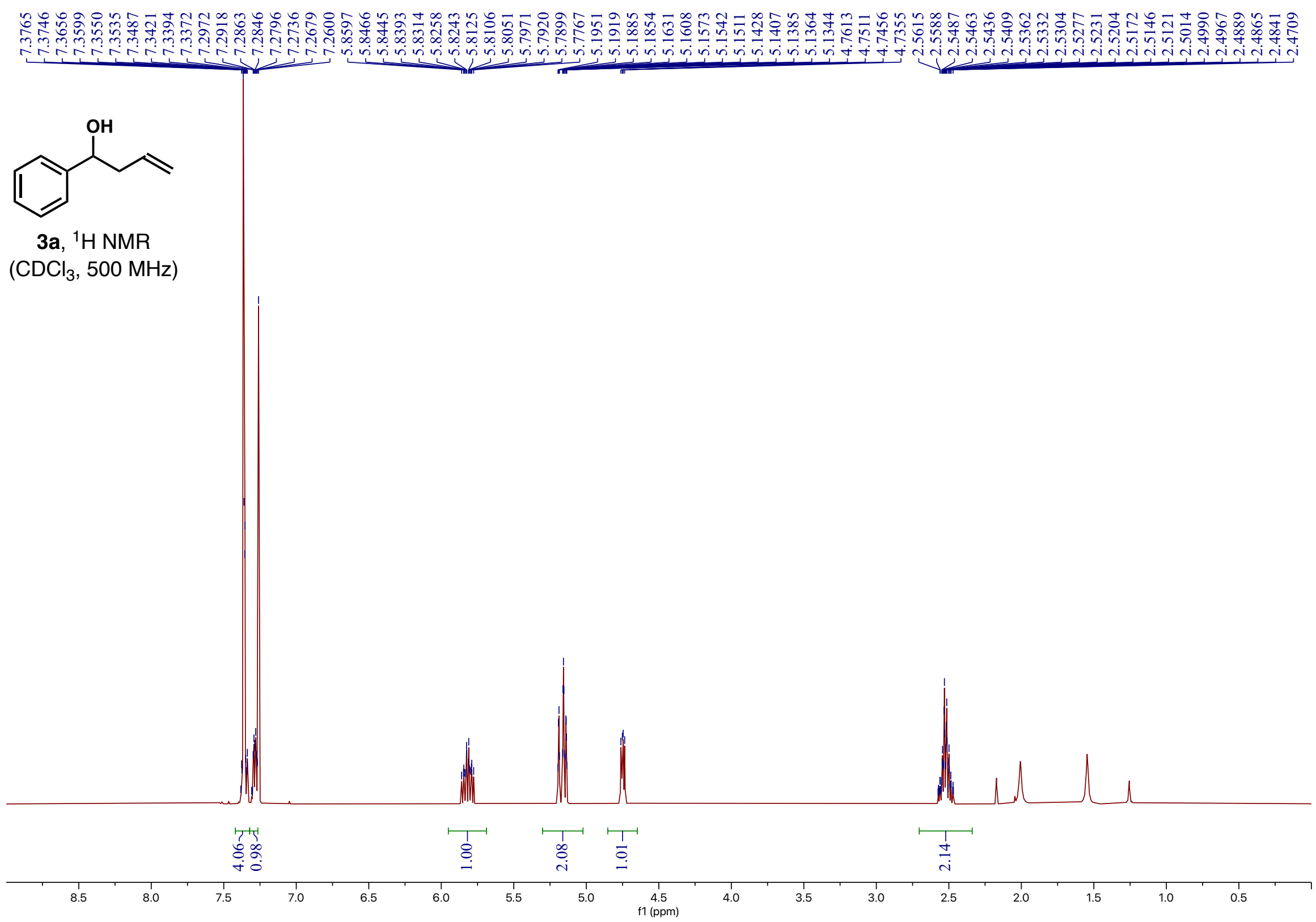
OH
3a, ¹H NMR
(CDCl₃, 500 MHz)
f1 (ppm)

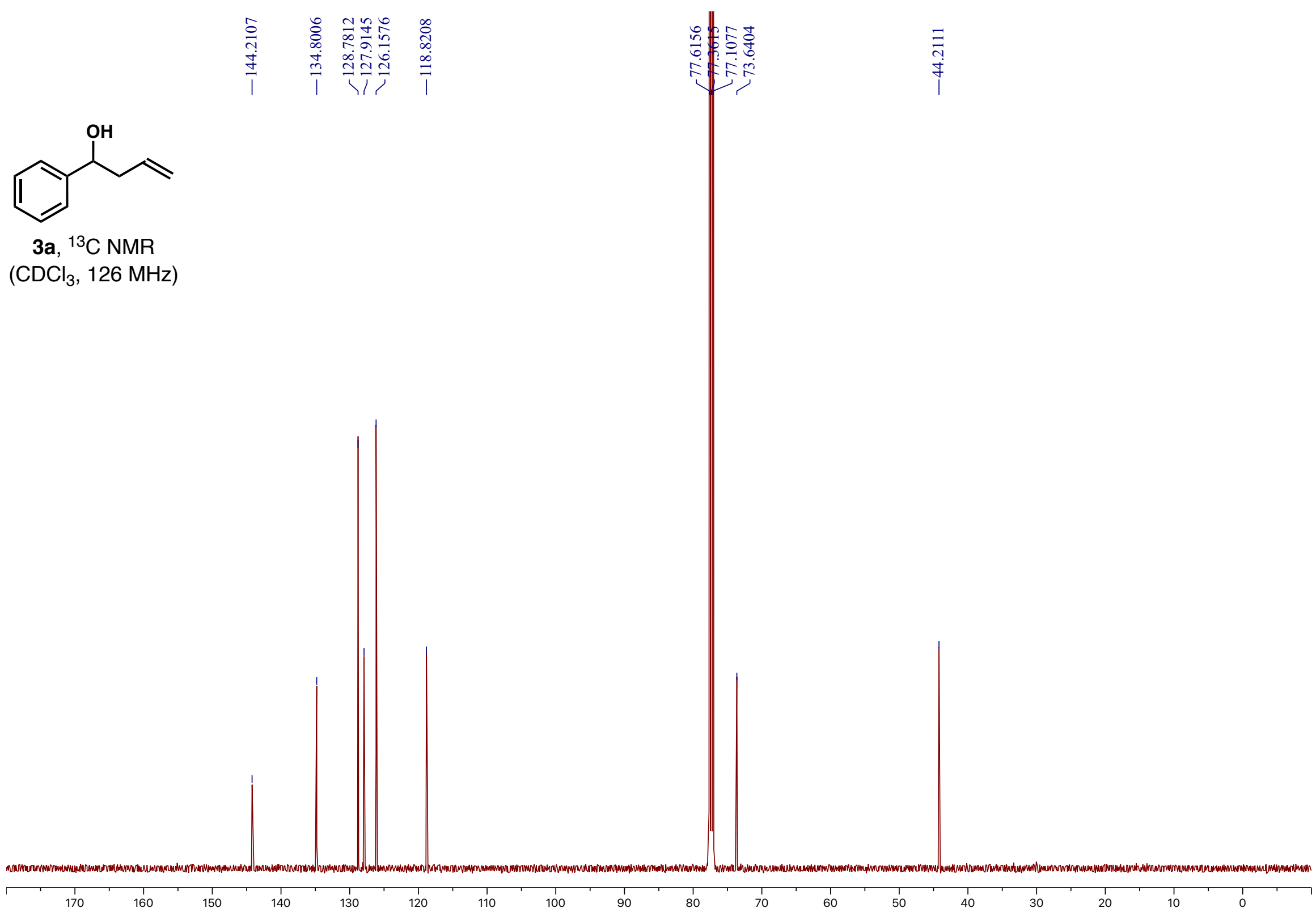
OH
3a, ¹³C NMR
(CDCl₃, 126 MHz)
f1 (ppm)

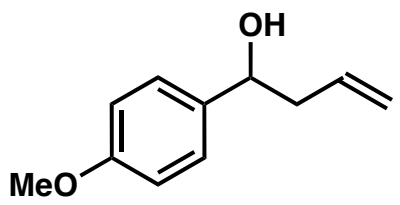

3b, ^{1}H NMR
($CDCl_3$, 500 MHz)

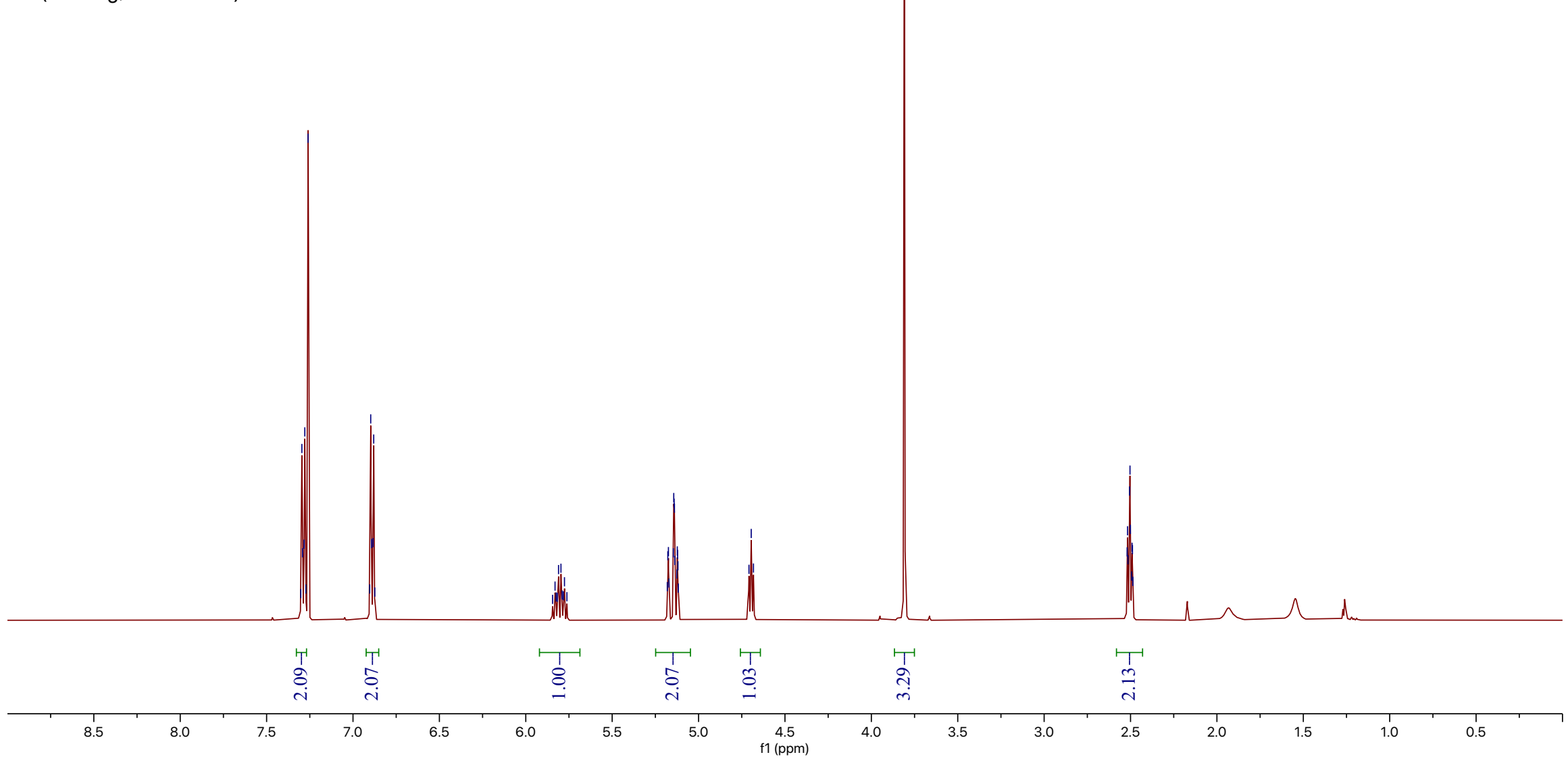

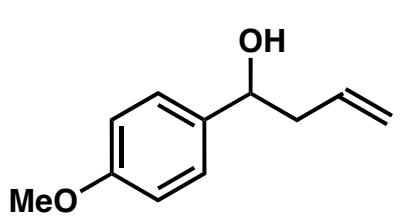

3b, ^{13}C NMR
($CDCl_3$, 126 MHz)

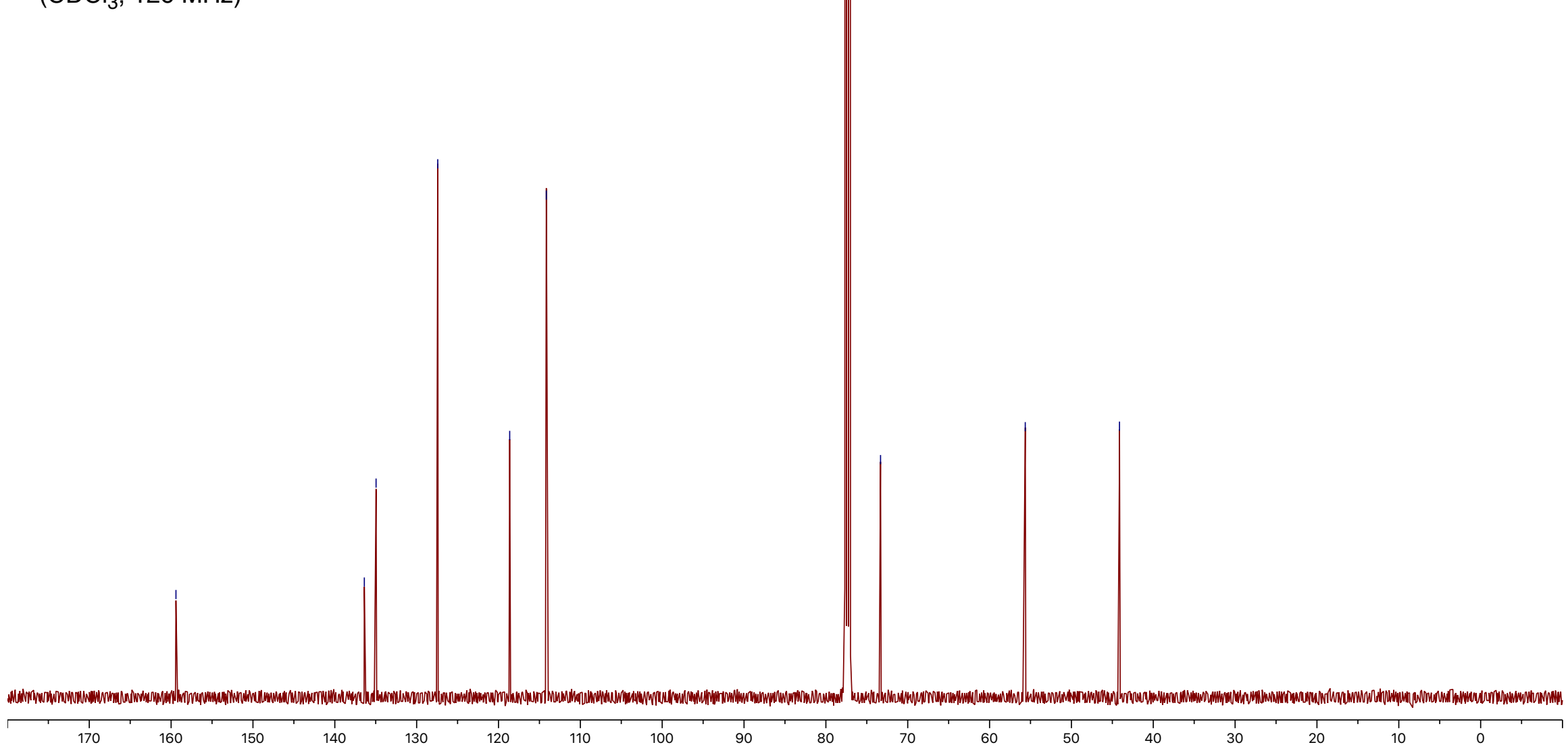

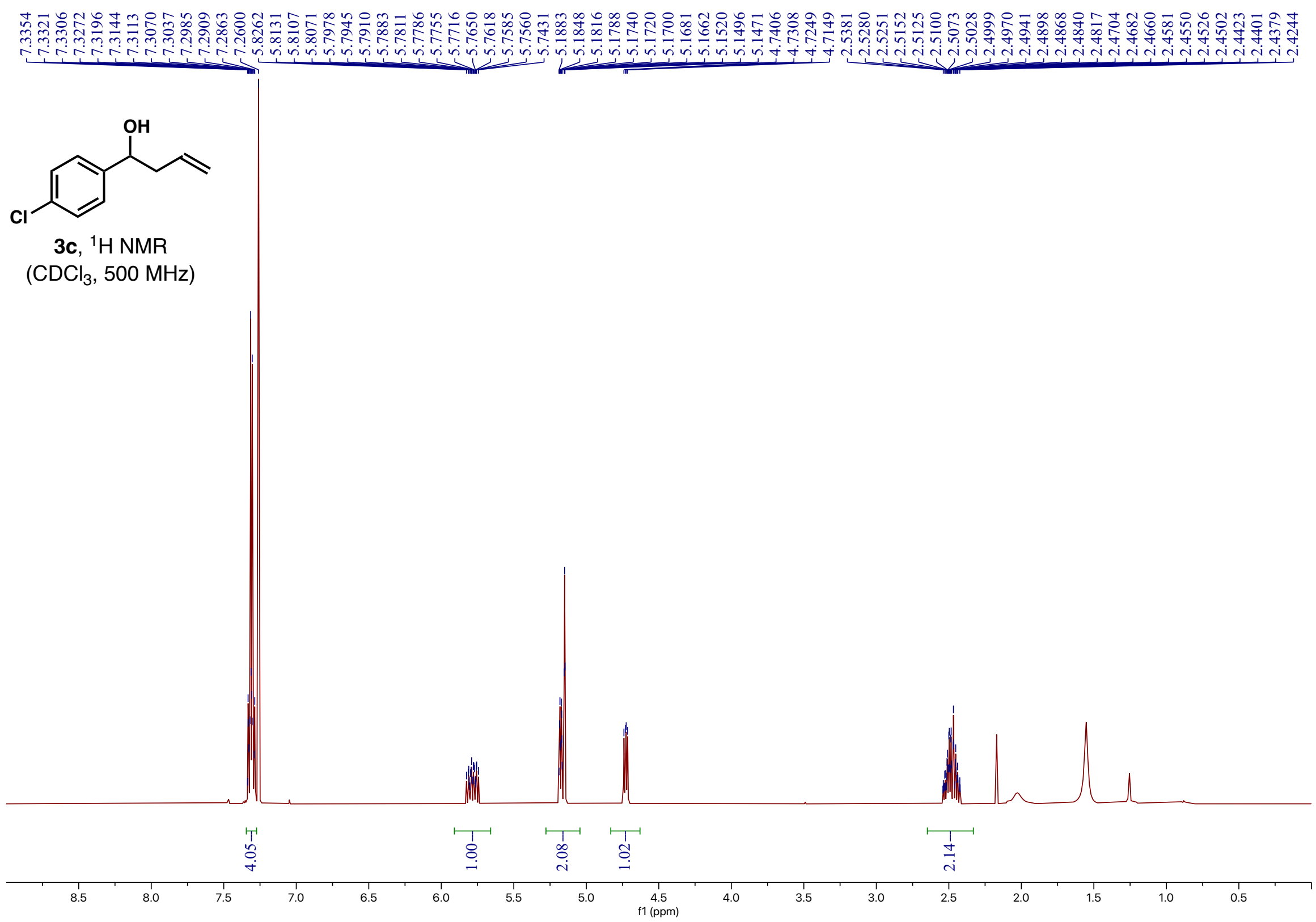

3c, ^{1}H NMR
($CDCl_3$, 500 MHz)

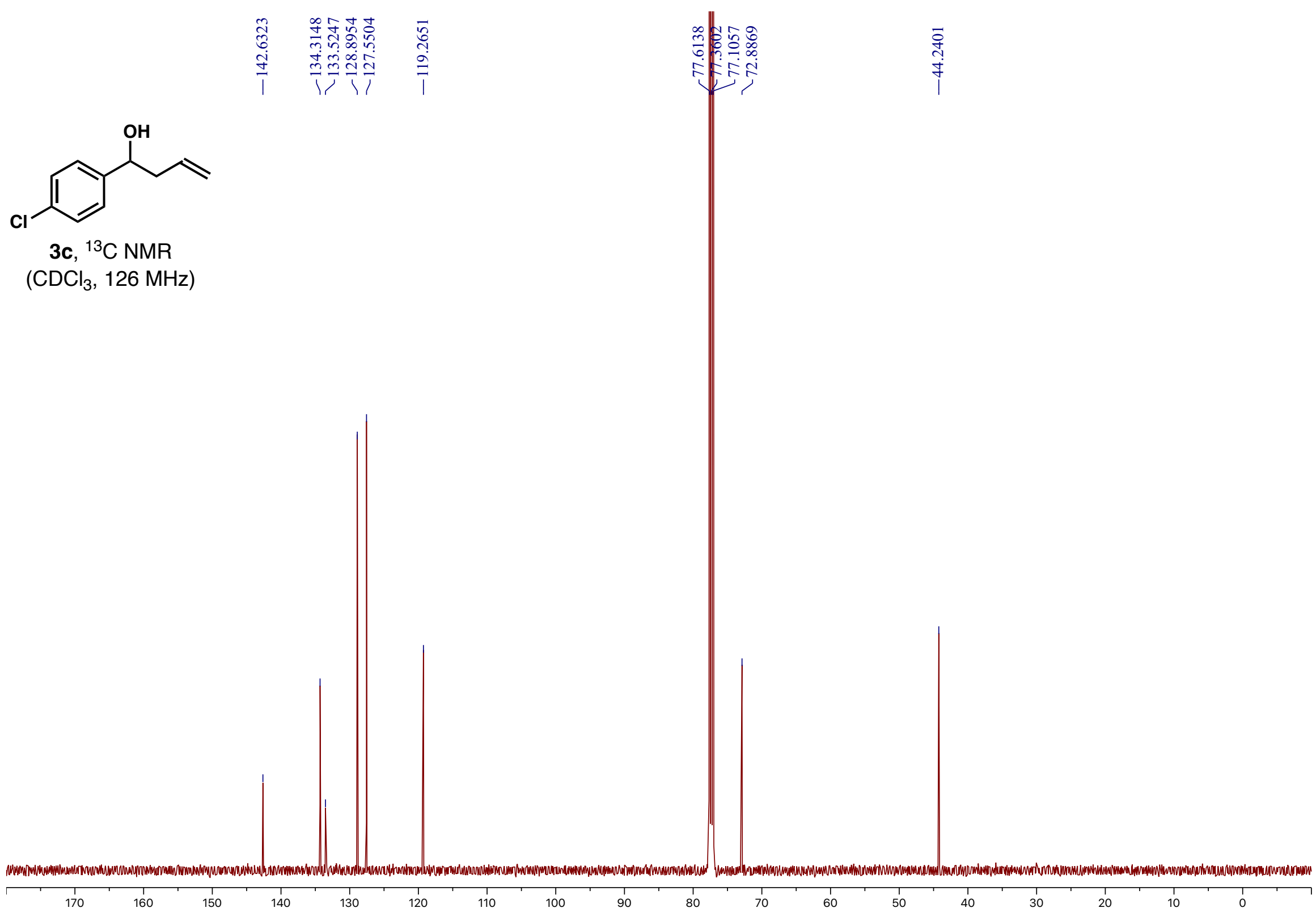

3c, ^{13}C NMR
($CDCl_3$, 126 MHz)

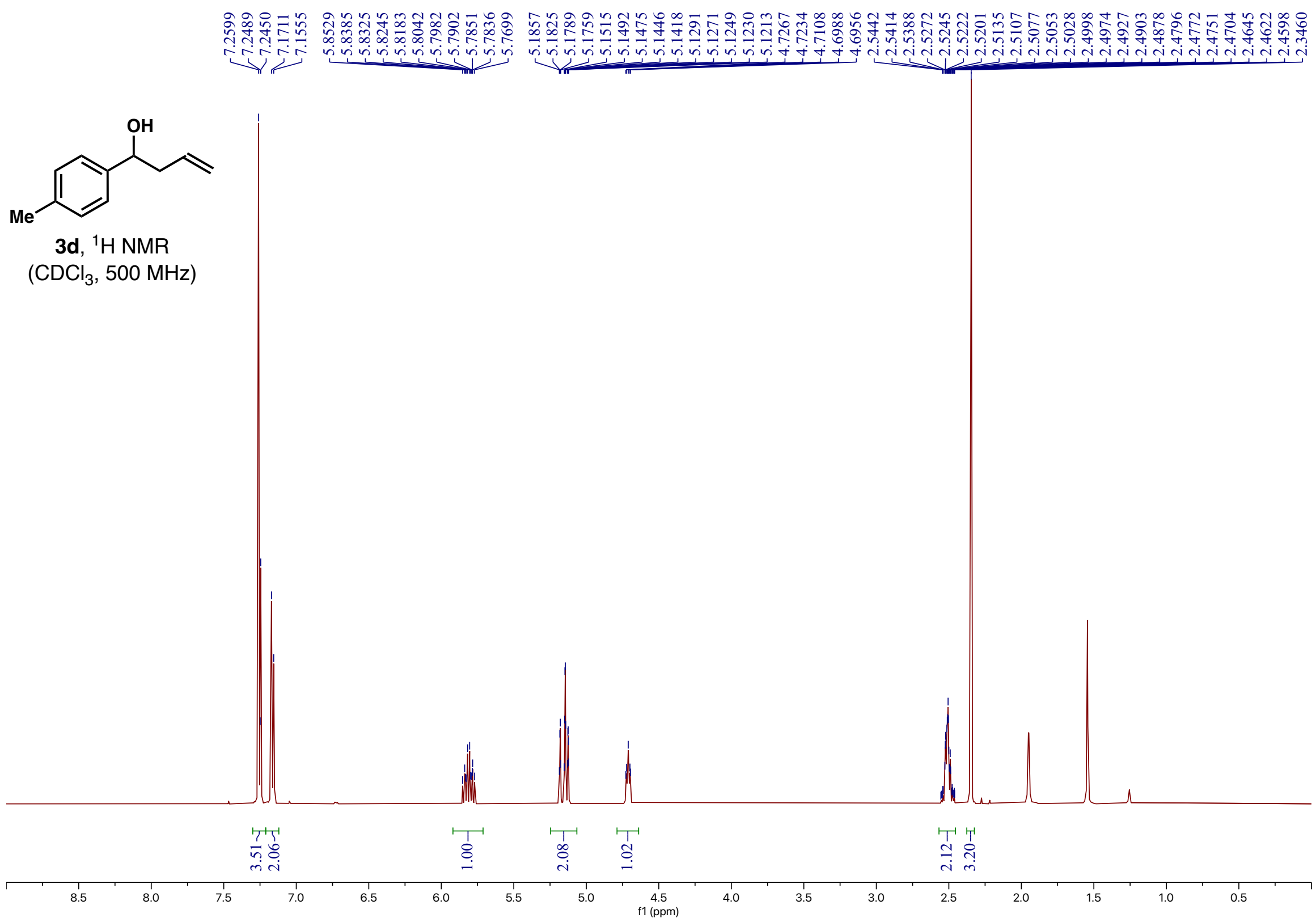

3d, ^{1}H NMR
($CDCl_3$, 500 MHz)

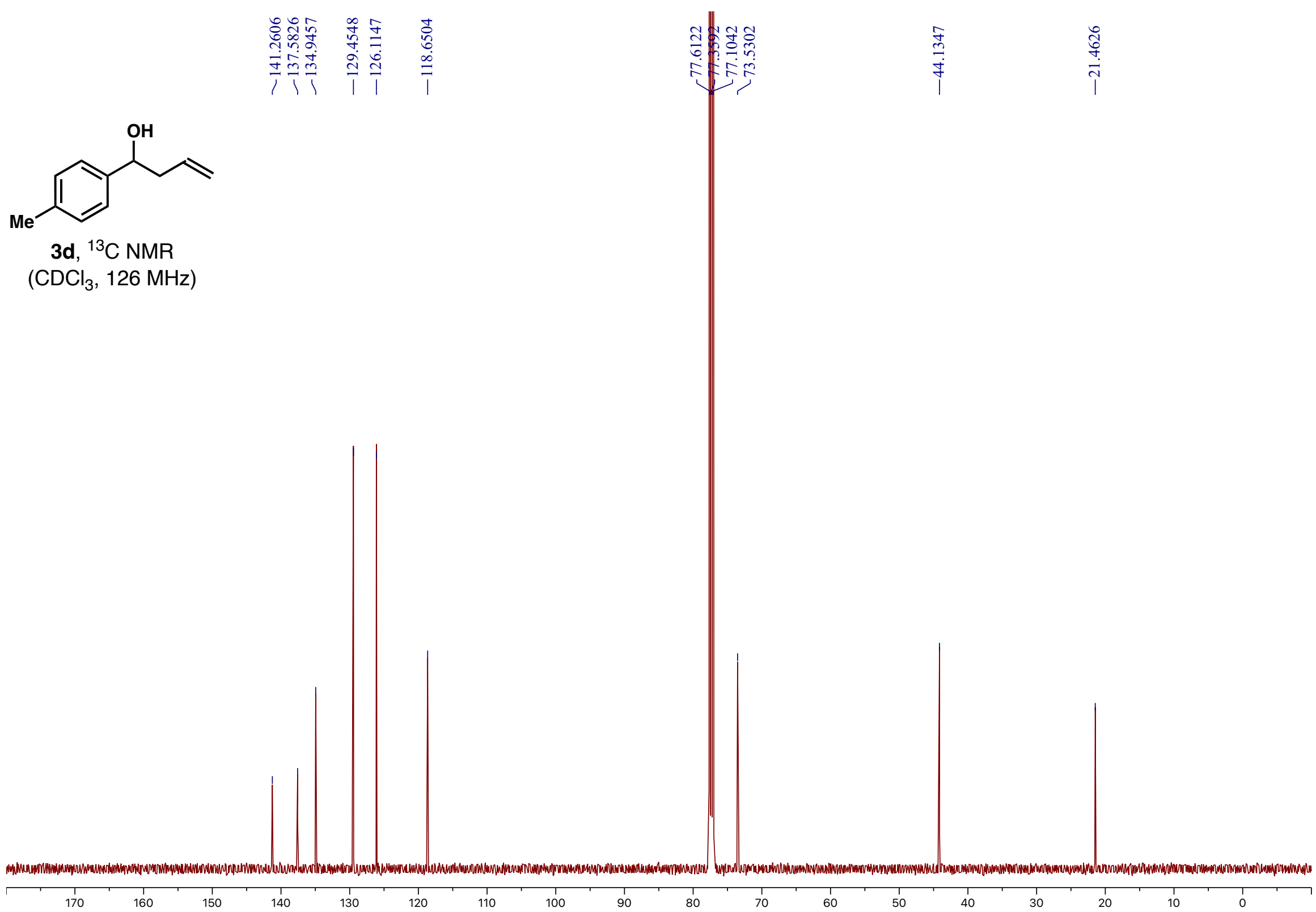

3d, ^{13}C NMR
($CDCl_3$, 126 MHz)

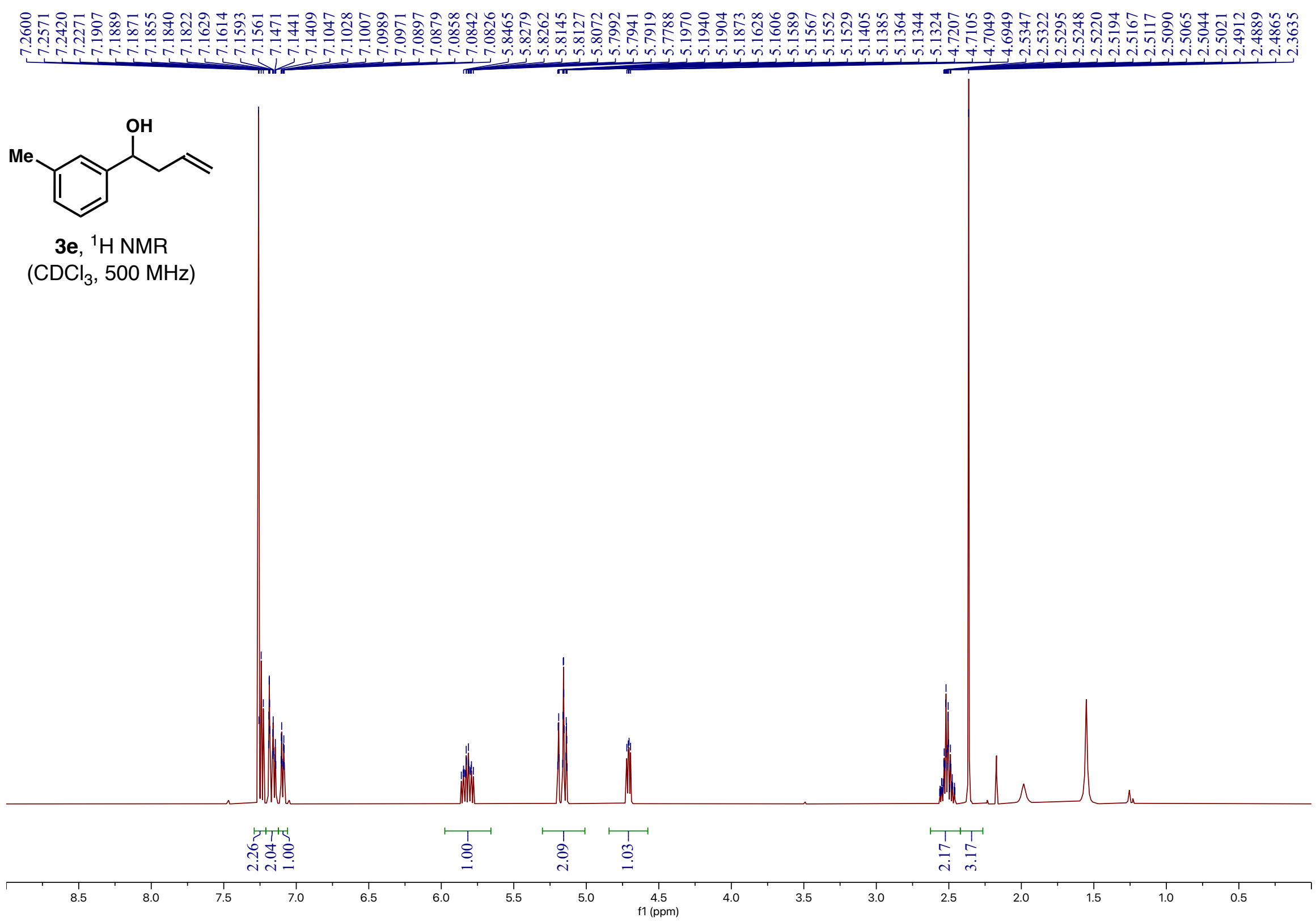
OH
Me
3e, 1H NMR
(CDCl3, 500 MHz)
7.2600 7.2571 7.2420 7.2271 7.1907 7.1889 7.1871 7.1855 7.1840 7.1822 7.1629 7.1614 7.1593 7.1561 7.1471 7.1441 7.1409 7.1047 7.1028 7.1007 7.0989 7.0971 7.0897 7.0879 7.0858 7.0842 7.0826 5.8465 5.8279 5.8262 5.8145 5.8127 5.8072 5.7992 5.7941 5.7919 5.7788 5.1970 5.1940 5.1904 5.1873 5.1628 5.1606 5.1589 5.1567 5.1552 5.1529 5.1405 5.1385 5.1364 5.1344 5.1324 4.7207 4.7105 4.7049 4.6949 2.5347 2.5322 2.5295 2.5248 2.5220 2.5194 2.5167 2.5117 2.5090 2.5065 2.5044 2.5021 2.4912 2.4889 2.4865 2.3635
2.26 2.04 1.00 1.00 2.09 1.03 2.17 3.17
8.5 8.0 7.5 7.0 6.5 6.0 5.5 5.0 4.5 4.0 3.5 3.0 2.5 2.0 1.5 1.0 0.5
f1 (ppm)

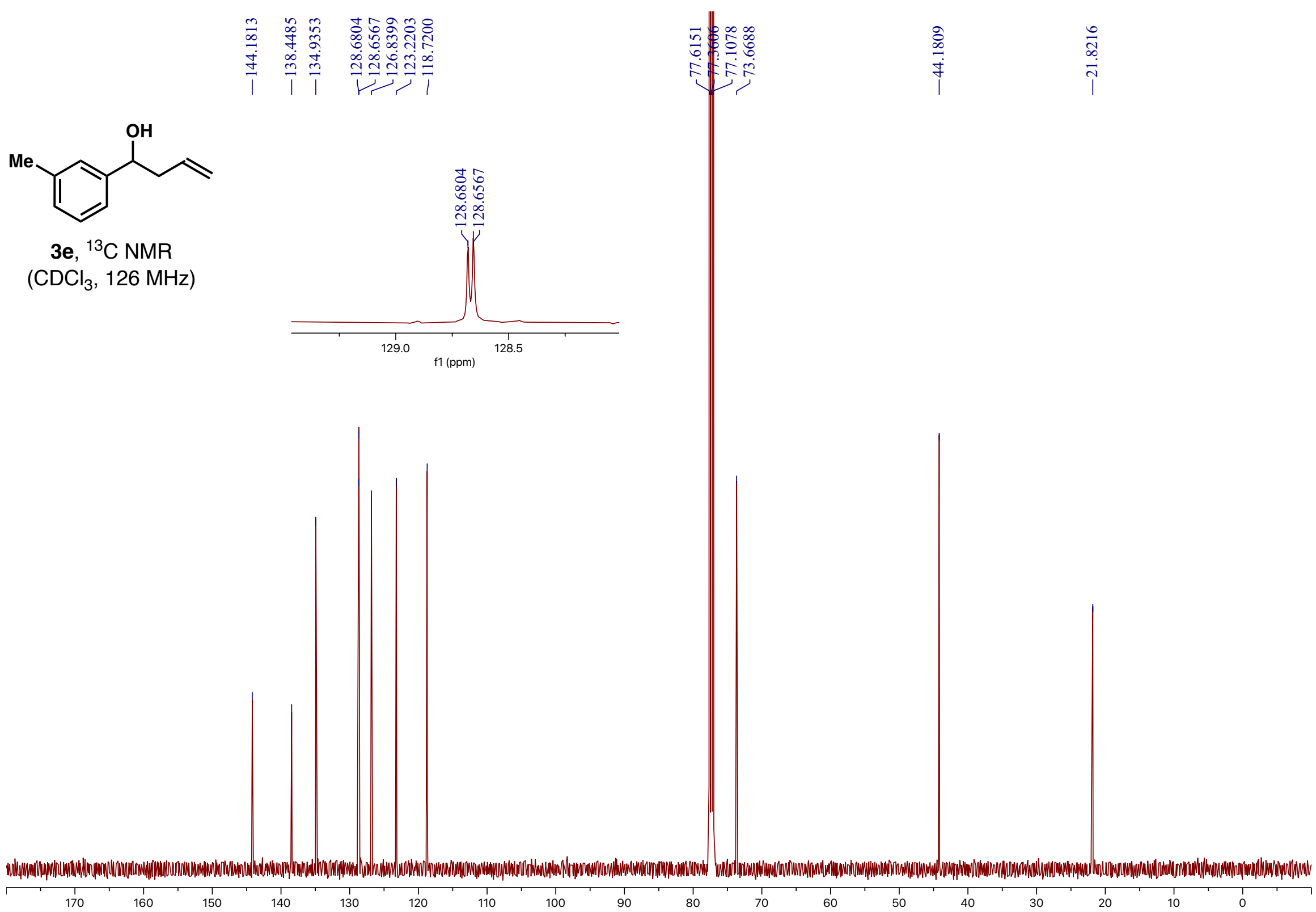
OH
Me
3e, 13C NMR
(CDCl3, 126 MHz)
144.1813 138.4485 134.9353 128.6804 128.6567 126.8399 123.2203 118.7200 77.6151 77.3606 77.1078 73.6688 44.1809 21.8216
128.6804 128.6567
129.0 128.5
f1 (ppm)
170 160 150 140 130 120 110 100 90 80 70 60 50 40 30 20 10 0
f1 (ppm)

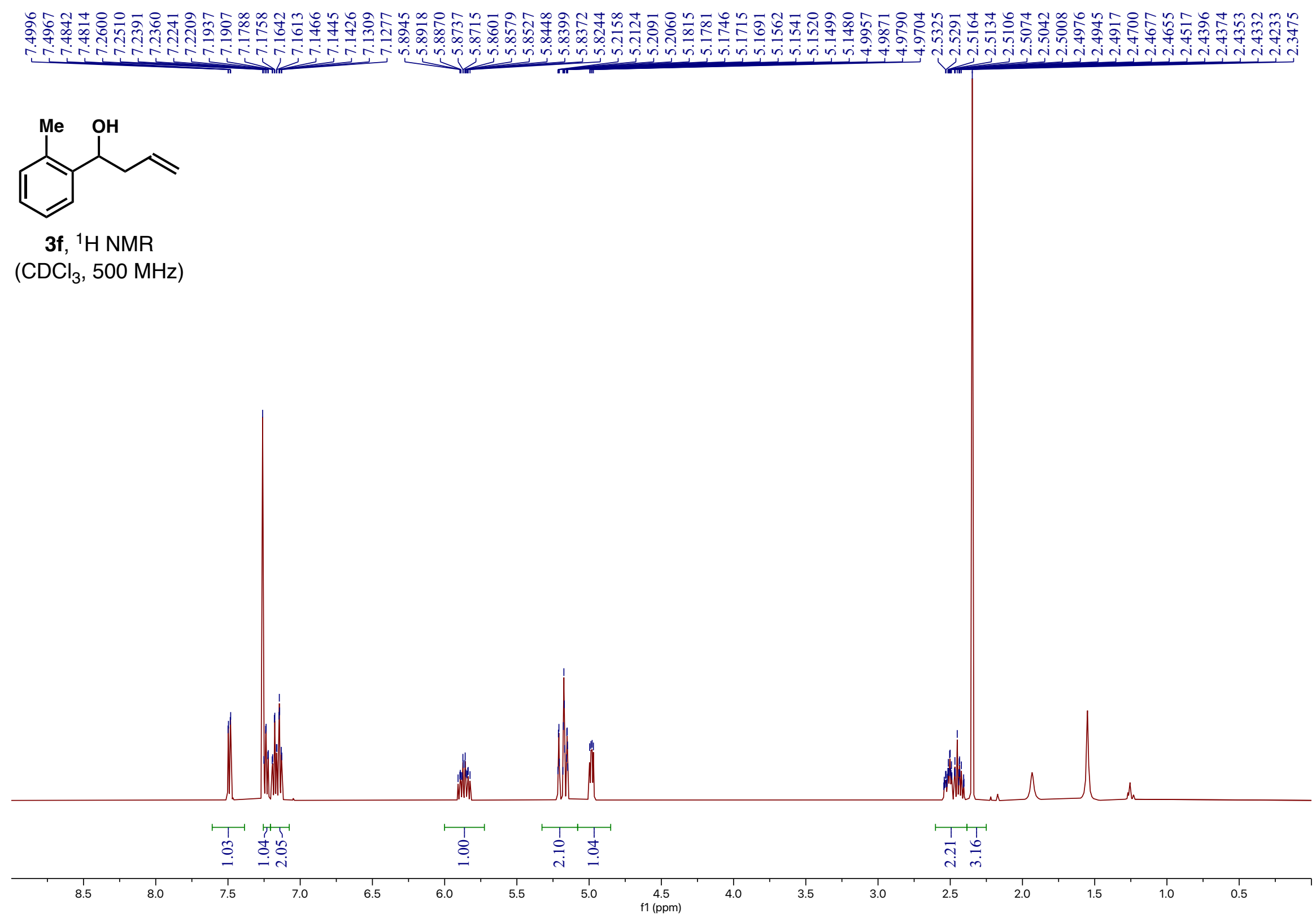
3f, ^{1}H NMR
(CDCl$_3$, 500 MHz)
f1 (ppm)

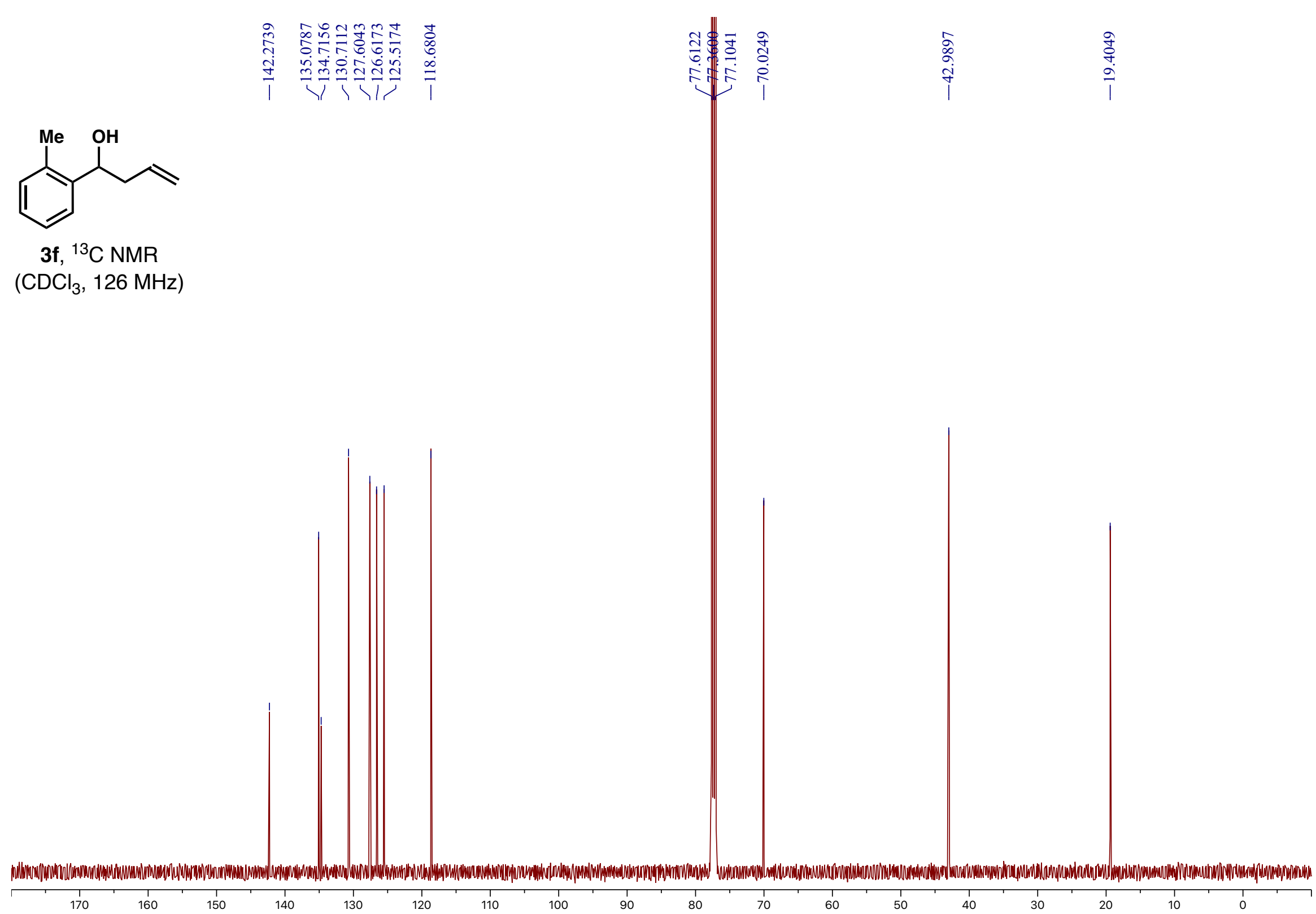
3f, ^{13}C NMR
(CDCl$_3$, 126 MHz)
142.2739
135.0787
134.7156
130.7112
127.6043
126.6173
125.5174
118.6804
77.6122
77.3600
77.1041
70.0249
42.9897
19.4049
f1 (ppm)

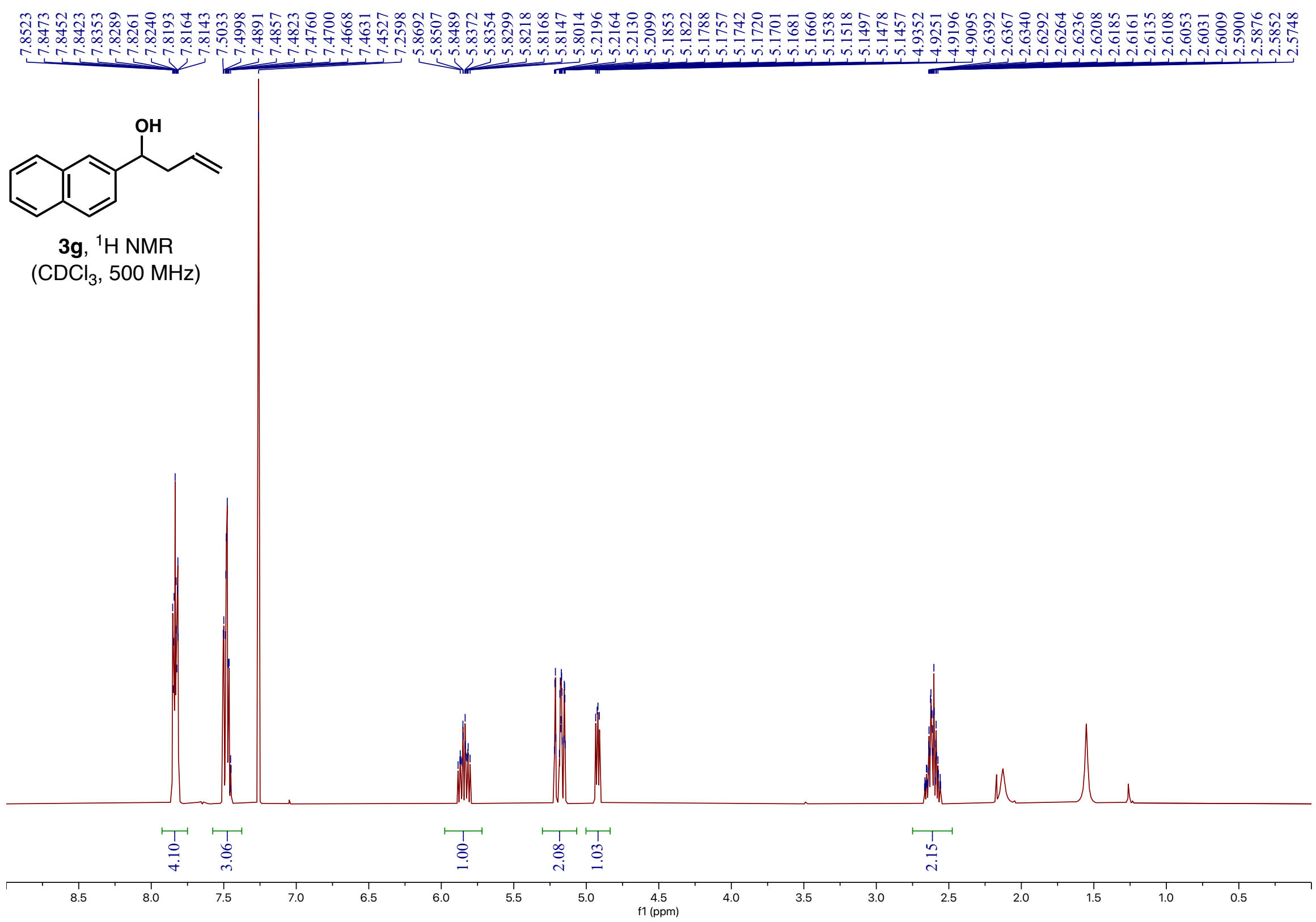

7.8523 7.8473 7.8452 7.8423 7.8353 7.8289 7.8261 7.8240 7.8193 7.8164 7.8143 7.5033 7.4998 7.4891 7.4857 7.4823 7.4760 7.4700 7.4668 7.4631 7.4527 7.2598 5.8692 5.8507 5.8489 5.8372 5.8354 5.8299 5.8218 5.8168 5.8147 5.8014 5.2196 5.2164 5.2130 5.2099 5.1853 5.1822 5.1788 5.1757 5.1742 5.1720 5.1701 5.1681 5.1660 5.1538 5.1518 5.1497 5.1478 5.1457 4.9352 4.9251 4.9196 4.9095 2.6392 2.6367 2.6340 2.6292 2.6264 2.6236 2.6208 2.6185 2.6161 2.6135 2.6108 2.6053 2.6031 2.6009 2.5900 2.5876 2.5852 2.5748
OH
3g, ^{1}H NMR
(CDCl$_3$, 500 MHz)
4.10
3.06
1.00
2.08
1.03
2.15
8.5 8.0 7.5 7.0 6.5 6.0 5.5 5.0 4.5 4.0 3.5 3.0 2.5 2.0 1.5 1.0 0.5
f1 (ppm)

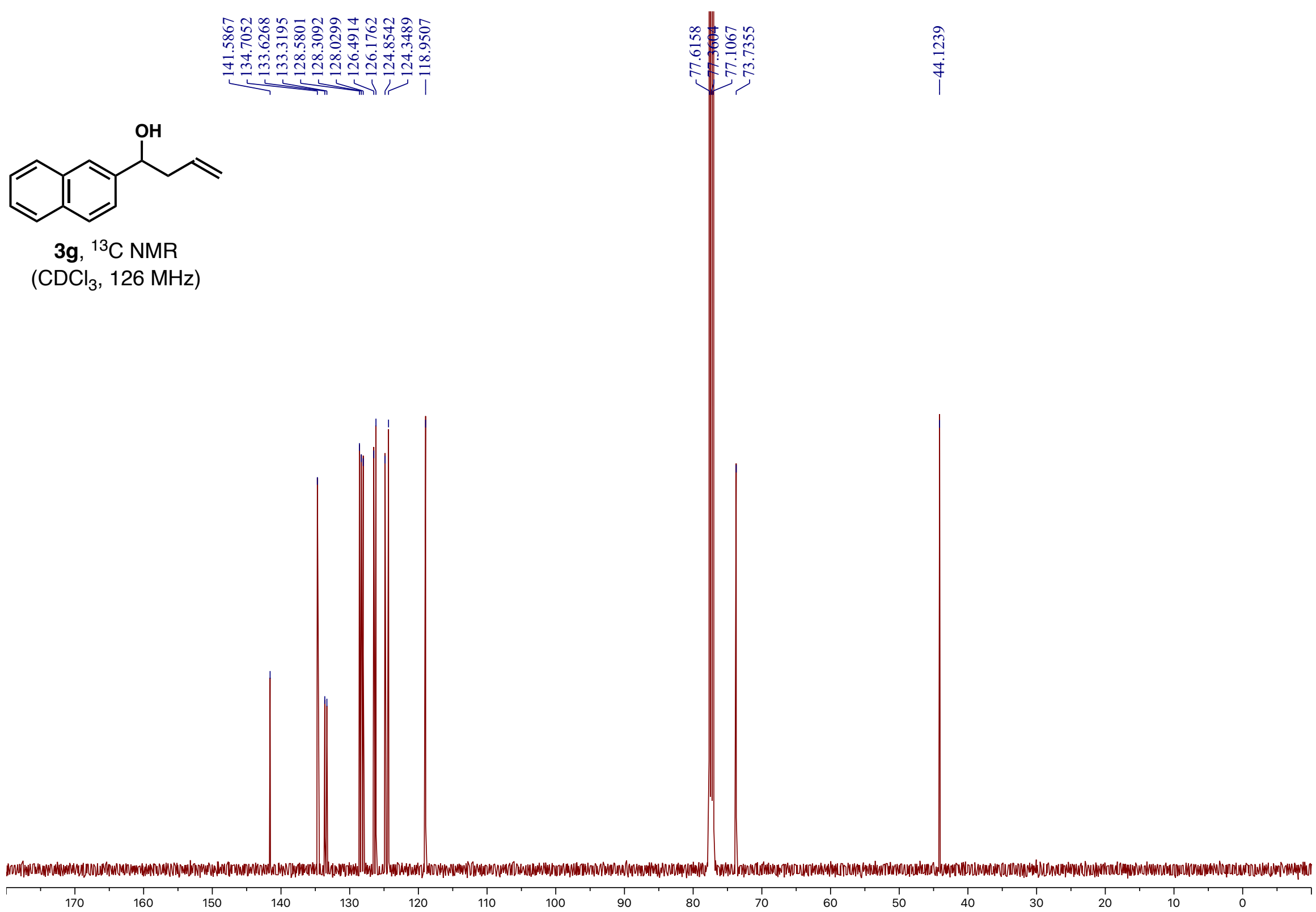

141.5867 134.7052 133.6268 133.3195 128.5801 128.3092 128.0299 126.4914 126.1762 124.8542 124.3489 118.9507
77.6158 77.3604 77.1067 73.7355
44.1239
OH
3g, ^{13}C NMR
(CDCl$_3$, 126 MHz)
170 160 150 140 130 120 110 100 90 80 70 60 50 40 30 20 10 0
f1 (ppm)

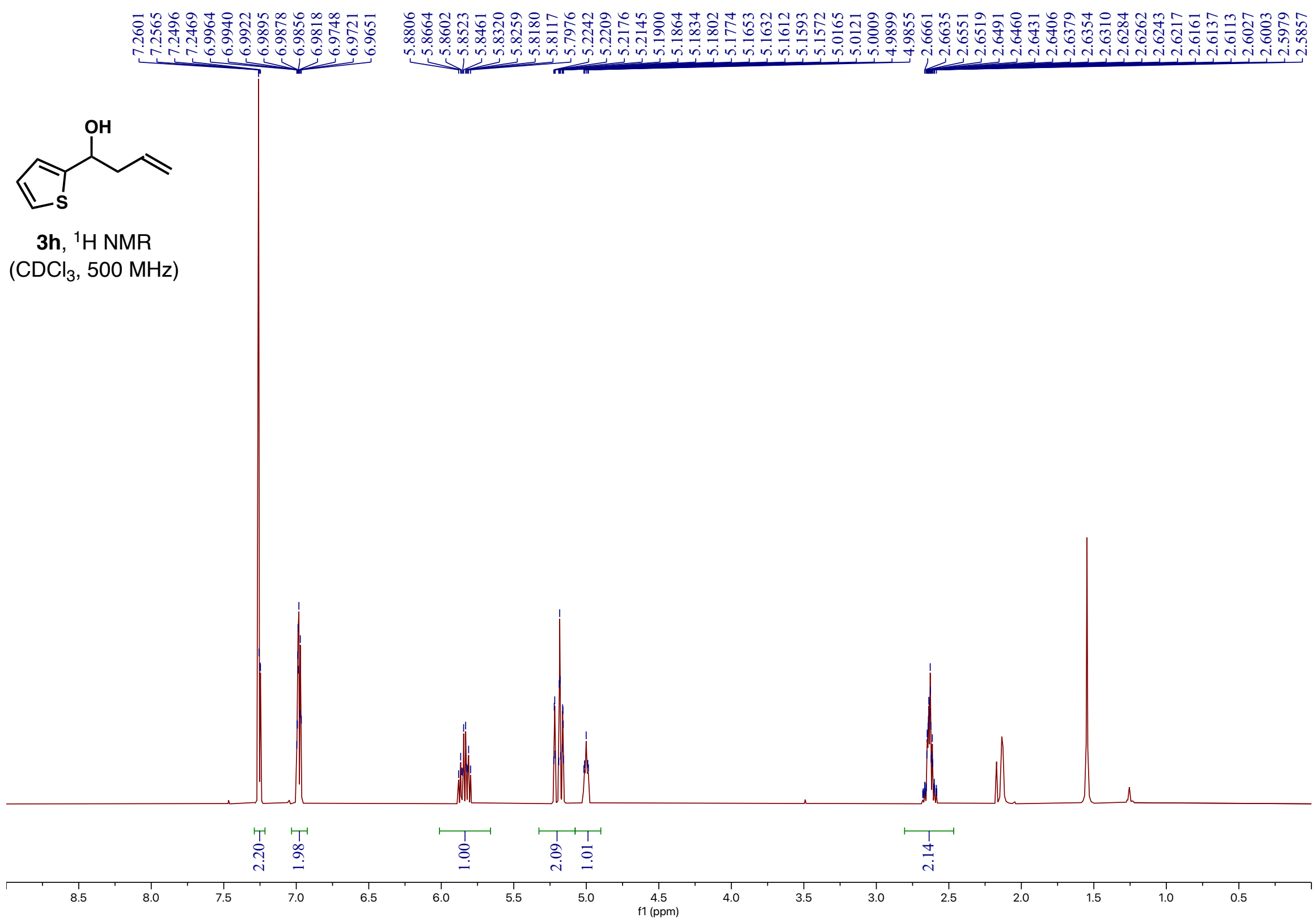

3h, ^{1}H NMR
($CDCl_3$, 500 MHz)

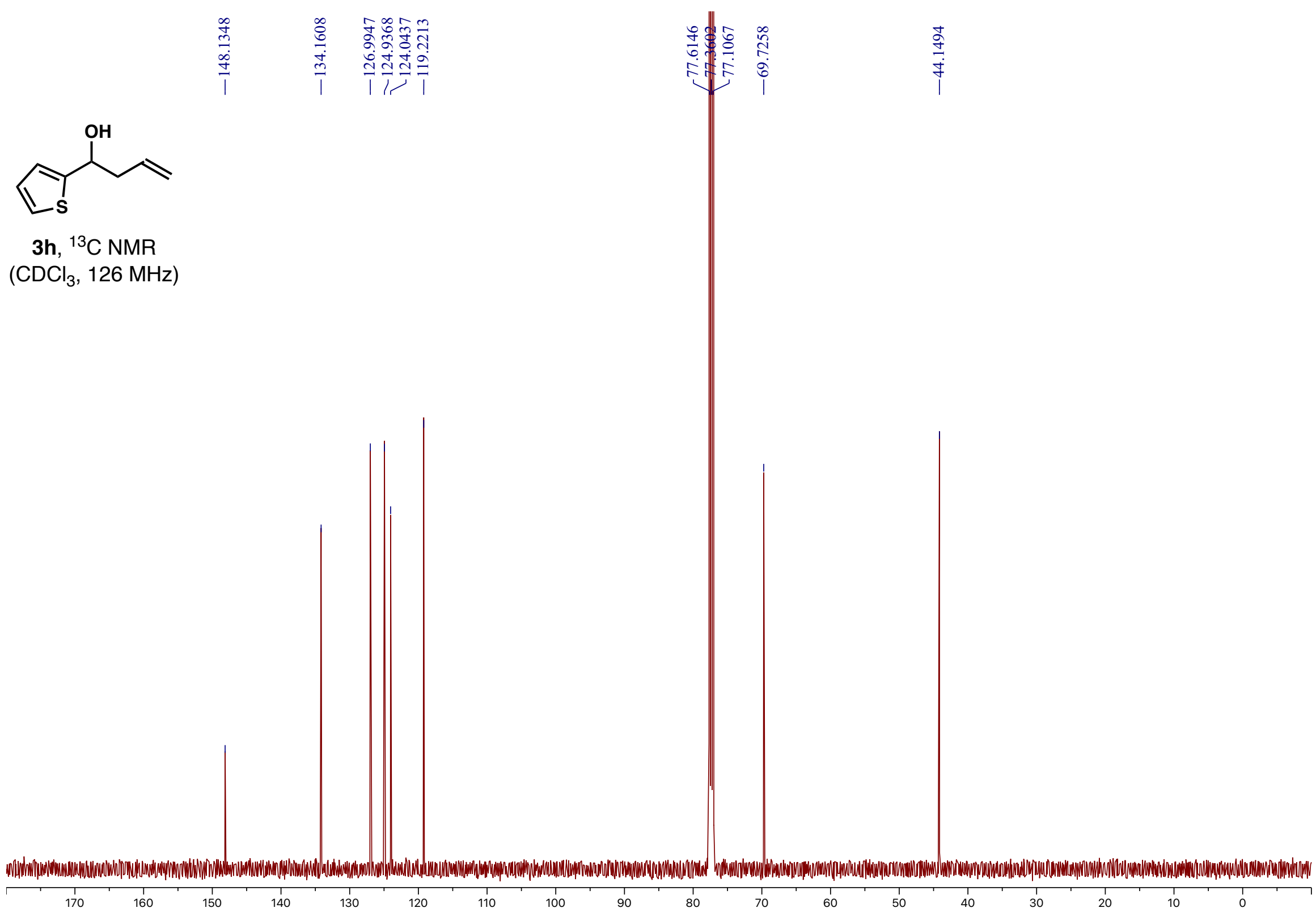

3h, ^{13}C NMR
($CDCl_3$, 126 MHz)

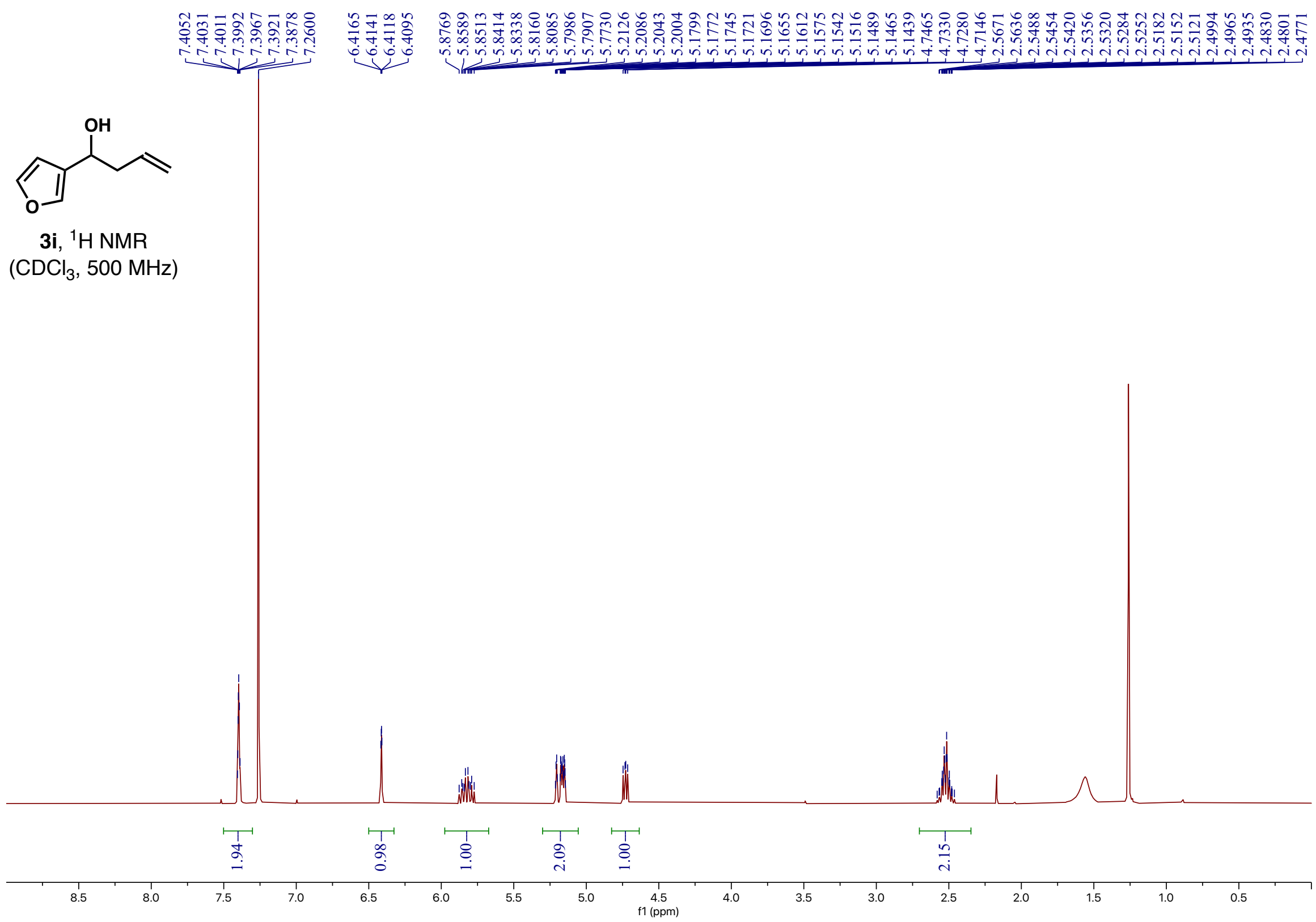

3i, ^1H NMR
($CDCl_3$, 500 MHz)

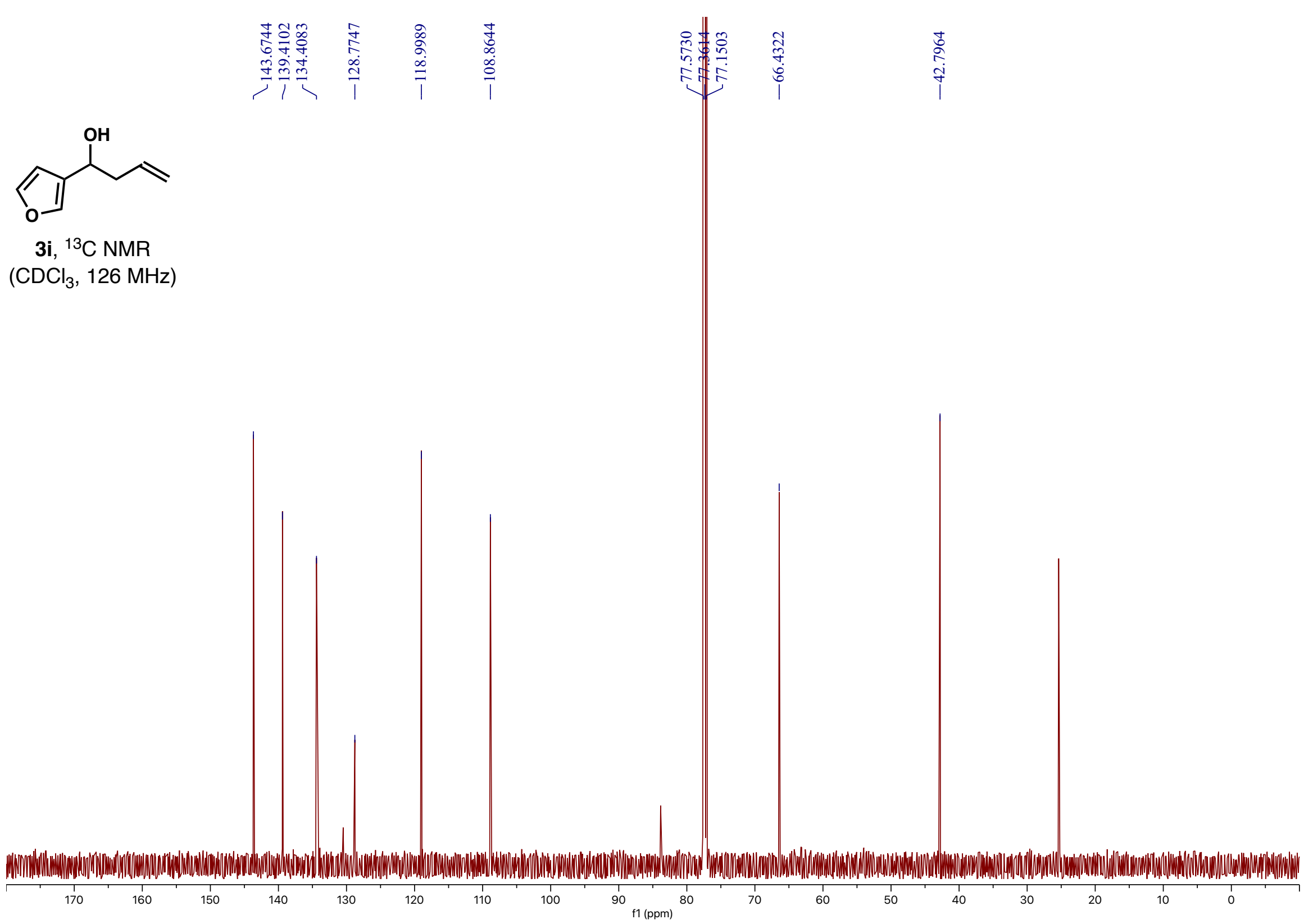

3i, ^{13}C NMR
($CDCl_3$, 126 MHz)

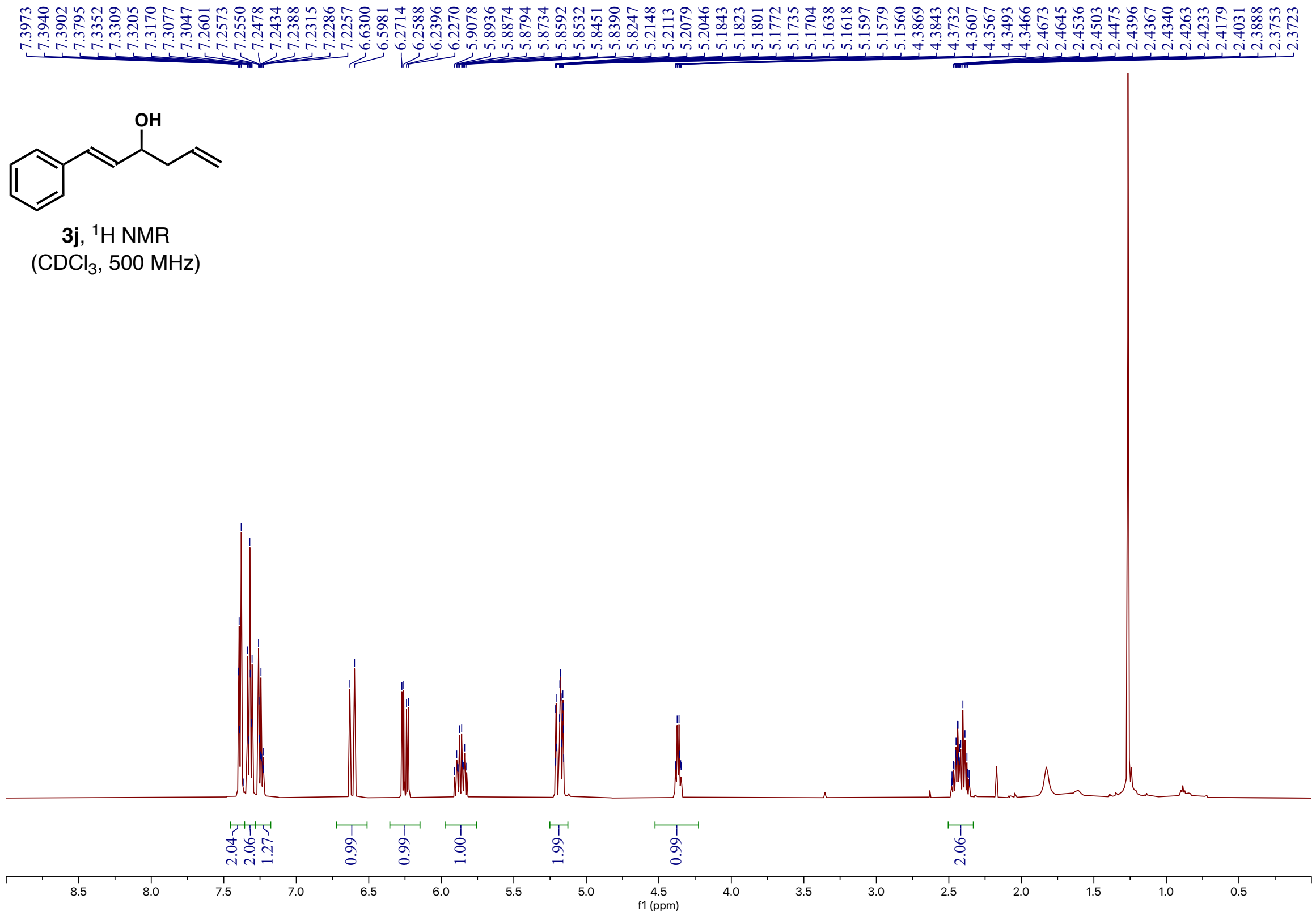
7.3973
7.3940
7.3902
7.3795
7.3352
7.3309
7.3205
7.3170
7.3077
7.3047
7.2601
7.2573
7.2550
7.2478
7.2434
7.2388
7.2315
7.2286
7.2257
6.6300
6.5981
6.2714
6.2588
6.2396
6.2270
5.9078
5.8936
5.8874
5.8794
5.8734
5.8592
5.8532
5.8451
5.8390
5.8247
5.2148
5.2113
5.2079
5.2046
5.1843
5.1823
5.1801
5.1772
5.1735
5.1704
5.1638
5.1618
5.1597
5.1579
5.1560
4.3869
4.3843
4.3732
4.3607
4.3567
4.3493
4.3466
2.4673
2.4645
2.4536
2.4503
2.4475
2.4396
2.4367
2.4340
2.4263
2.4233
2.4179
2.4031
2.3888
2.3753
2.3723
OH
3j, 1H NMR
(CDCl3, 500 MHz)
2.04
2.06
1.27
0.99
0.99
1.00
1.99
0.99
2.06
f1 (ppm)

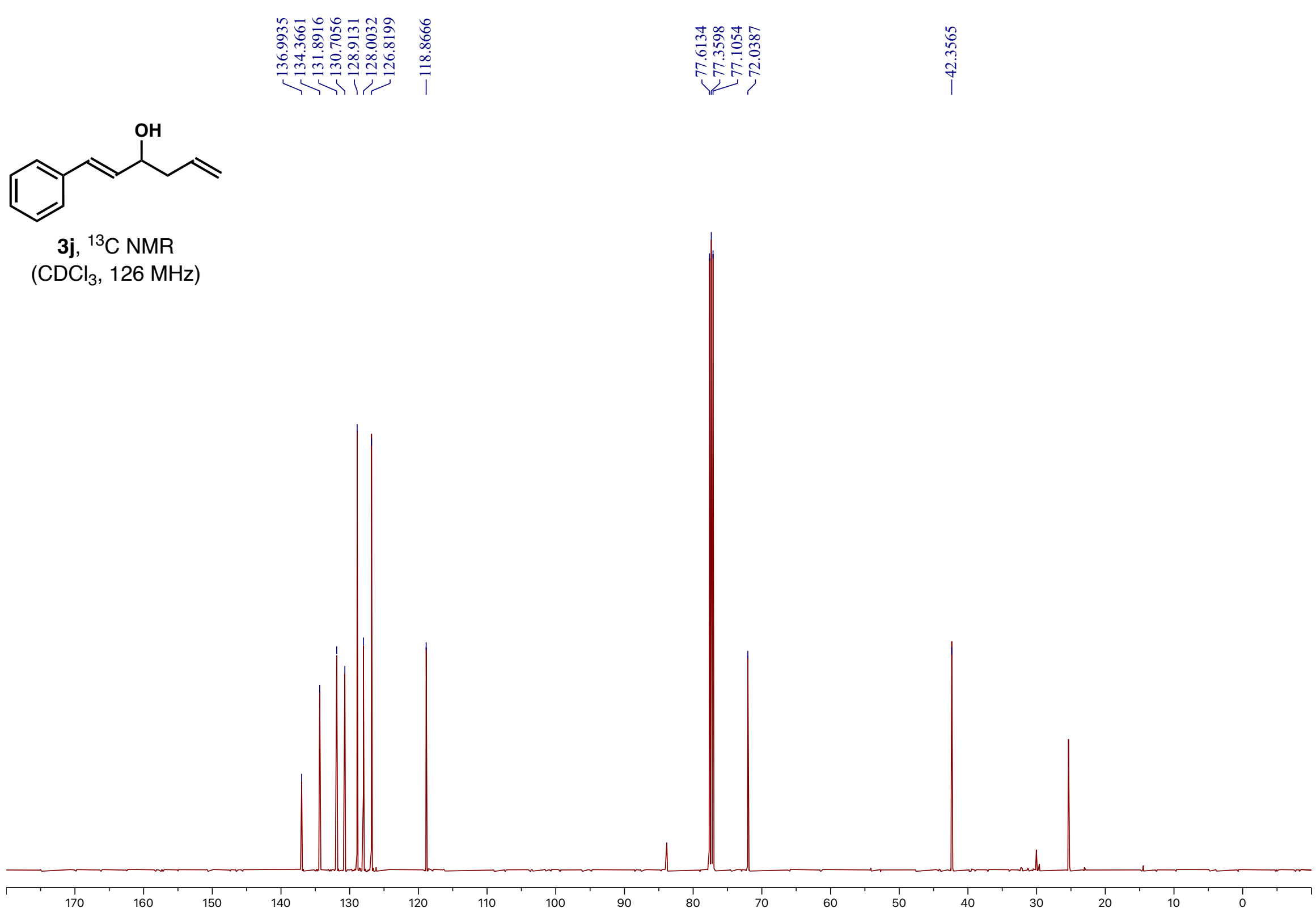
136.9935
134.3661
131.8916
130.7056
128.9131
128.0032
126.8199
118.8666
77.6134
77.3598
77.1054
72.0387
42.3565
OH
3j, 13C NMR
(CDCl3, 126 MHz)
f1 (ppm)

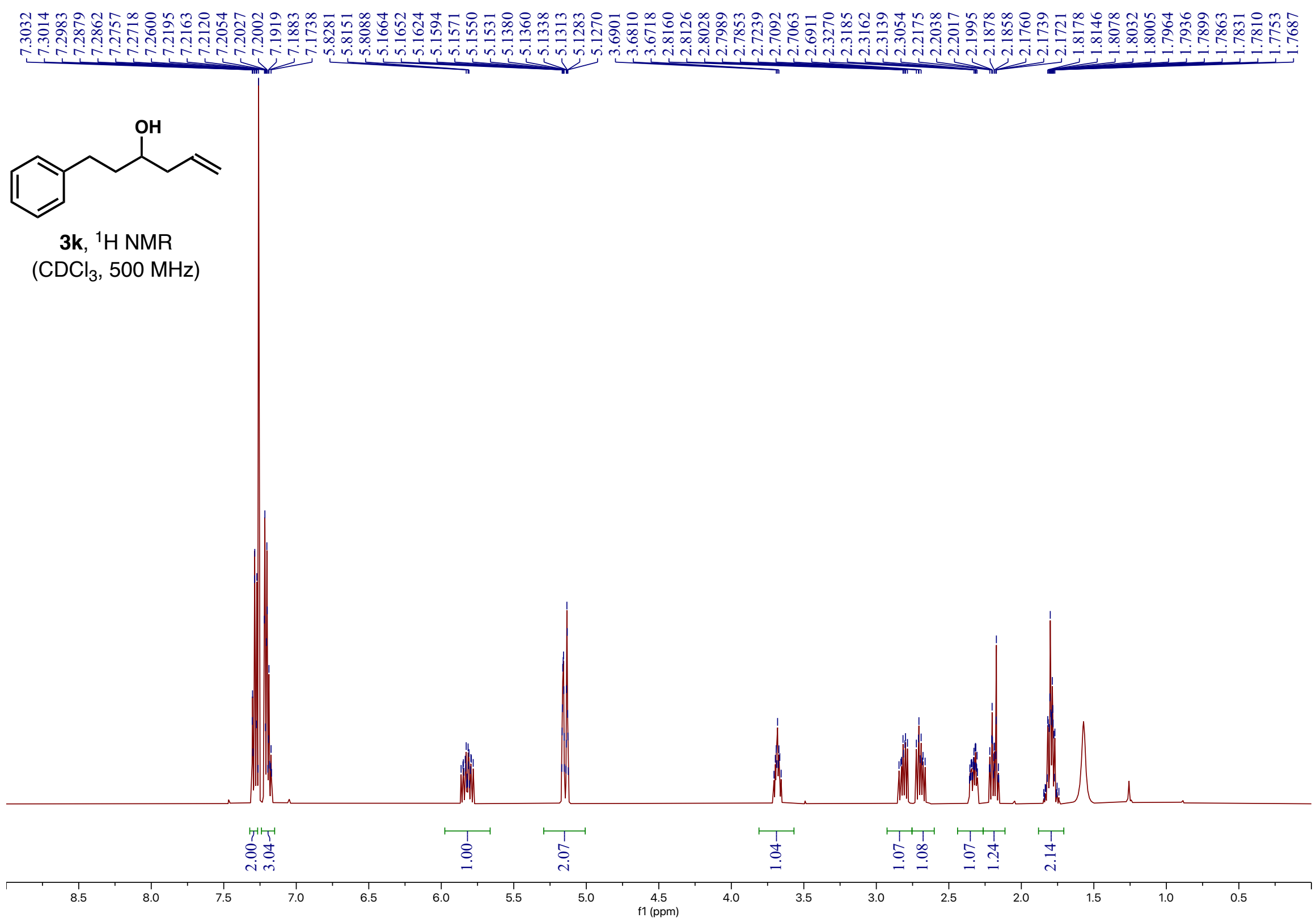
7.3032 7.3014 7.2983 7.2879 7.2862 7.2757 7.2718 7.2600 7.2195 7.2163 7.2120 7.2054 7.2027 7.2002 7.1919 7.1883 7.1738 5.8281 5.8151 5.8088 5.1664 5.1652 5.1624 5.1594 5.1571 5.1550 5.1531 5.1380 5.1360 5.1338 5.1313 5.1283 5.1270 3.6901 3.6810 3.6718 2.8160 2.8126 2.8028 2.7989 2.7853 2.7239 2.7092 2.7063 2.6911 2.3270 2.3185 2.3162 2.3139 2.3054 2.2175 2.2038 2.2017 2.1995 2.1878 2.1858 2.1760 2.1739 2.1721 1.8178 1.8146 1.8078 1.8032 1.8005 1.7964 1.7936 1.7899 1.7863 1.7831 1.7810 1.7753 1.7687
OH
3k, 1H NMR
(CDCl3, 500 MHz)
2.00 3.04 1.00 2.07 1.04 1.07 1.08 1.07 1.24 2.14
8.5 8.0 7.5 7.0 6.5 6.0 5.5 5.0 4.5 4.0 3.5 3.0 2.5 2.0 1.5 1.0 0.5
f1 (ppm)

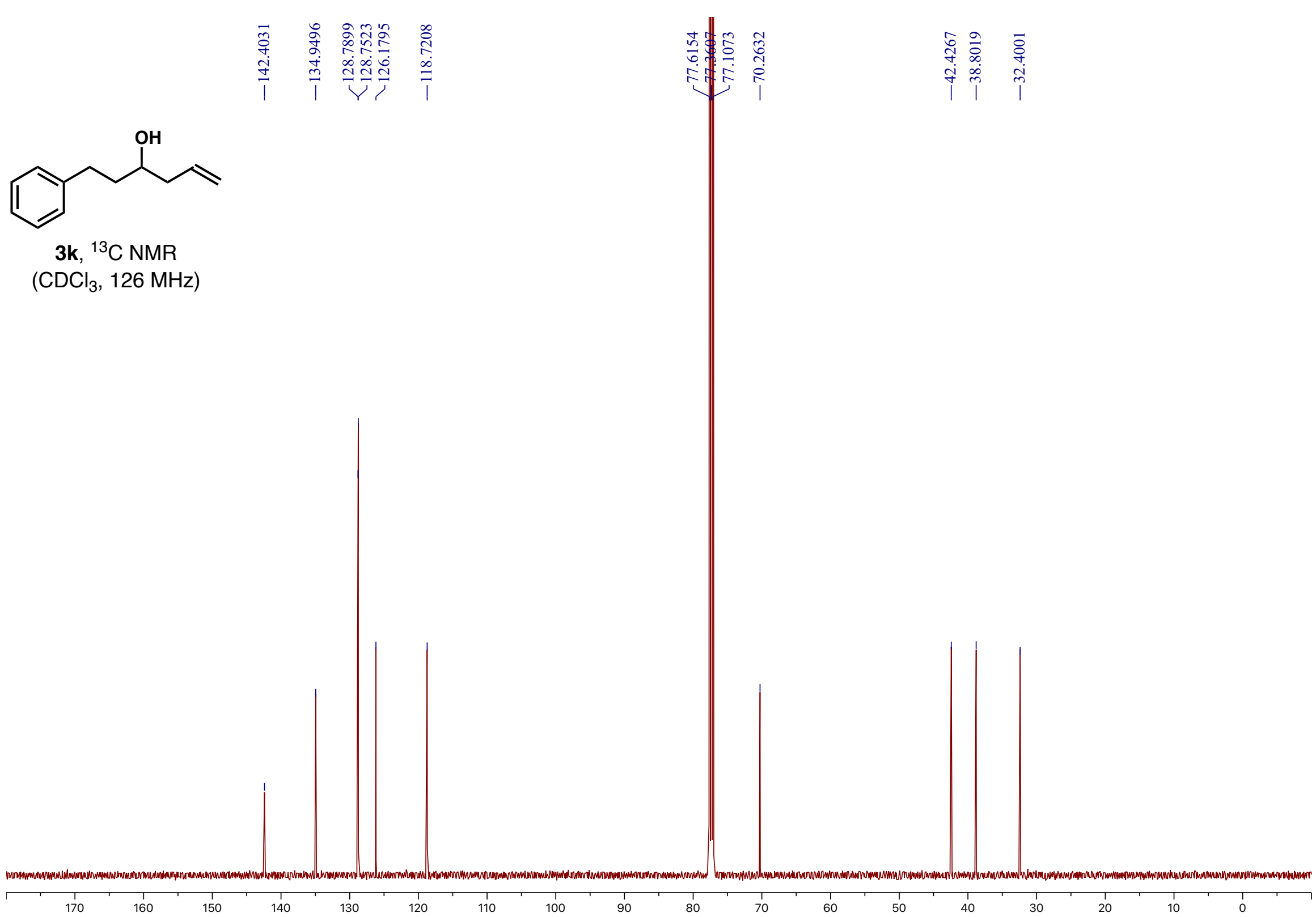
142.4031 134.9496 128.7899 128.7523 126.1795 118.7208 77.6154 77.3607 77.1073 70.2632 42.4267 38.8019 32.4001
OH
3k, 13C NMR
(CDCl3, 126 MHz)
170 160 150 140 130 120 110 100 90 80 70 60 50 40 30 20 10 0
f1 (ppm)

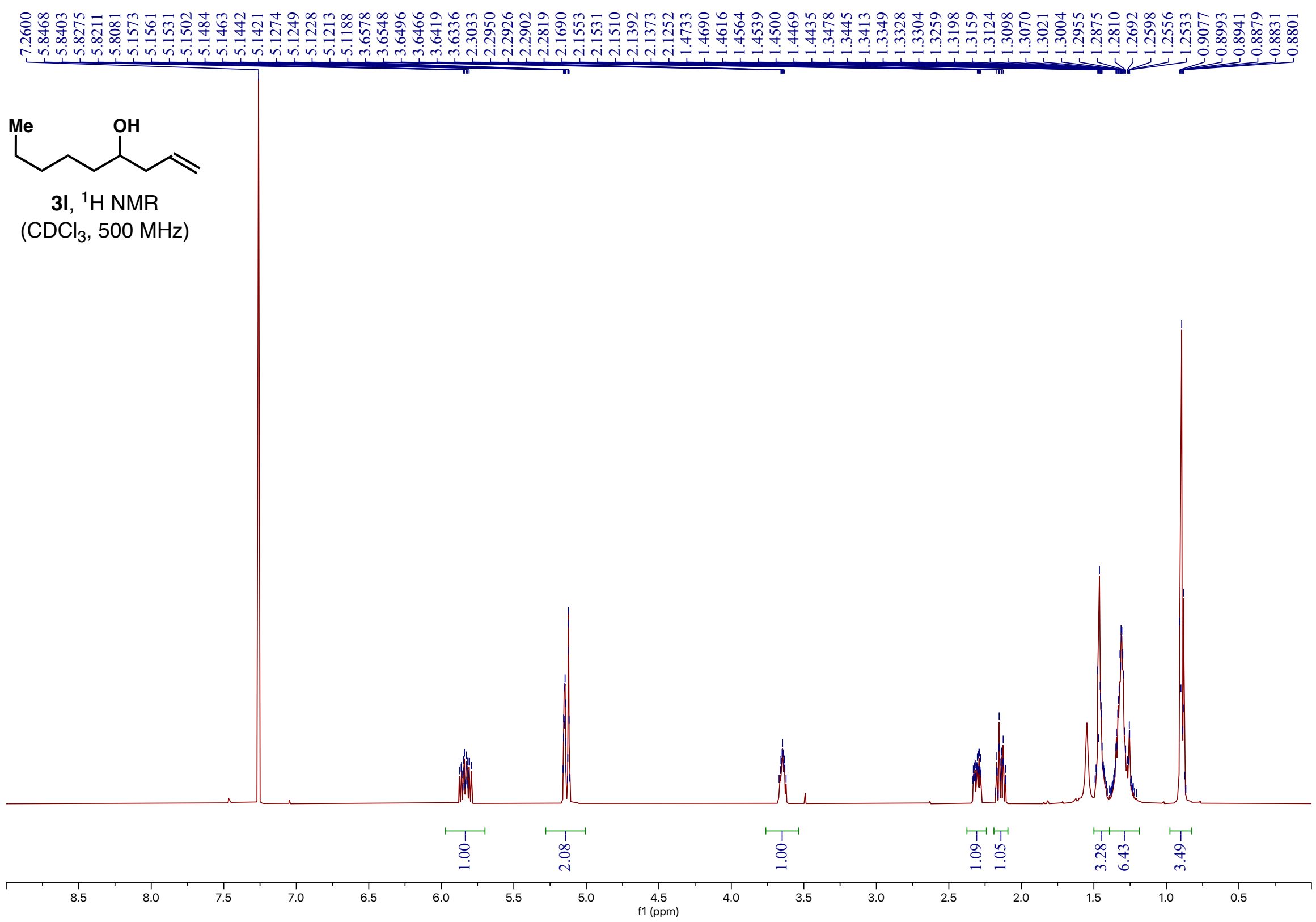
Me
OH
3l, ¹H NMR
(CDCl₃, 500 MHz)
f1 (ppm)

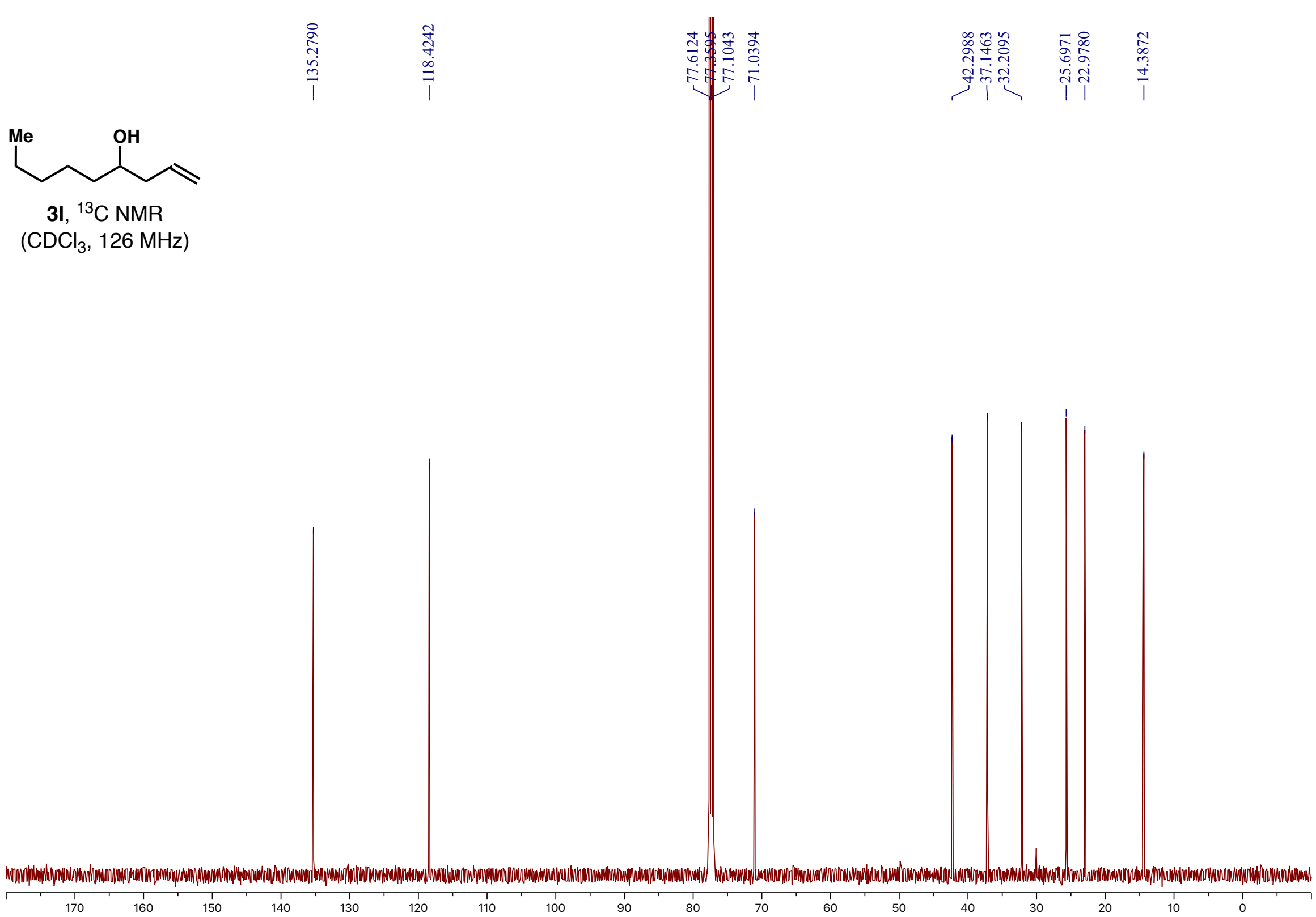
Me
OH
3l, ¹³C NMR
(CDCl₃, 126 MHz)
f1 (ppm)

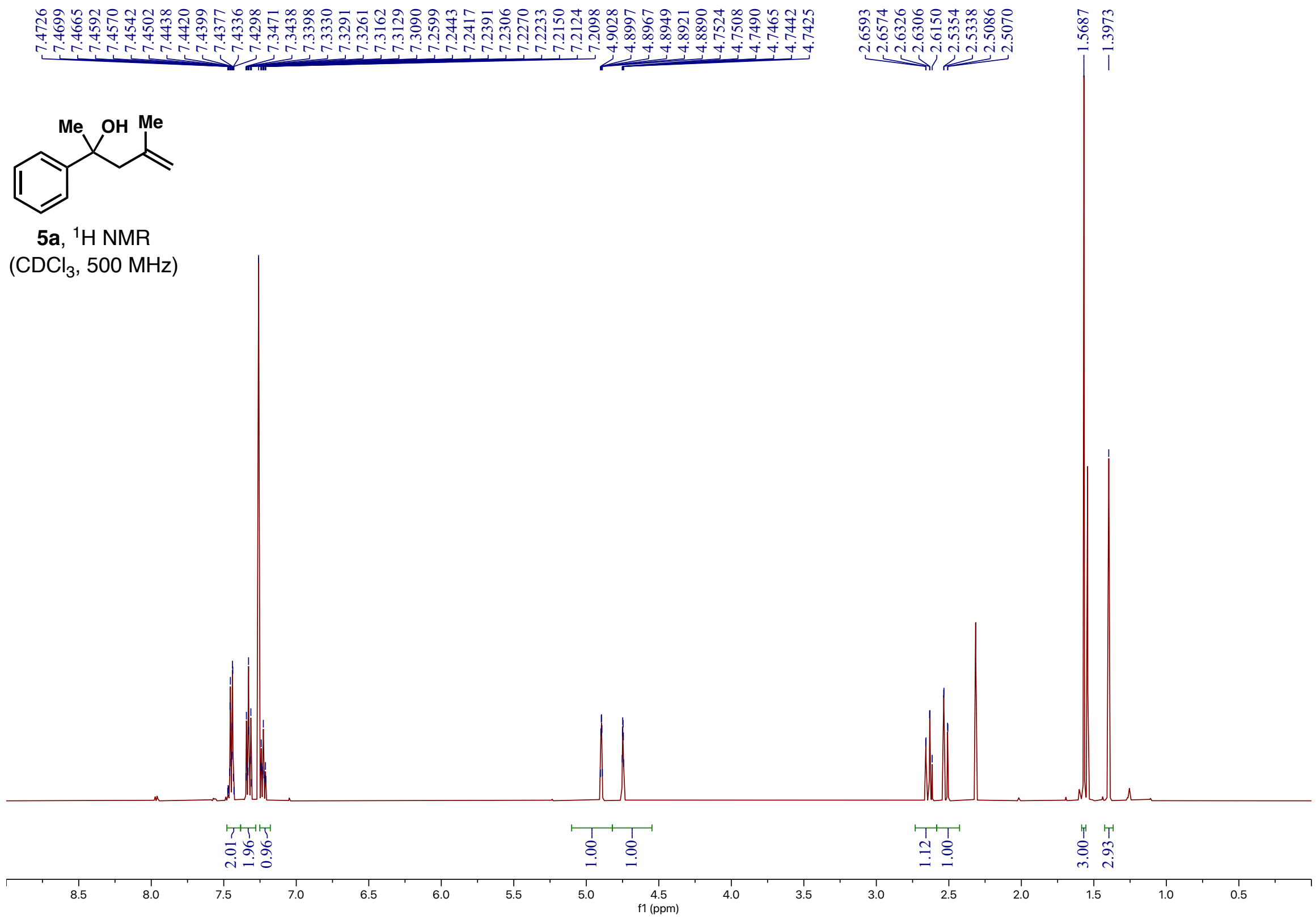

5a, ^{1}H NMR
($CDCl_3$, 500 MHz)

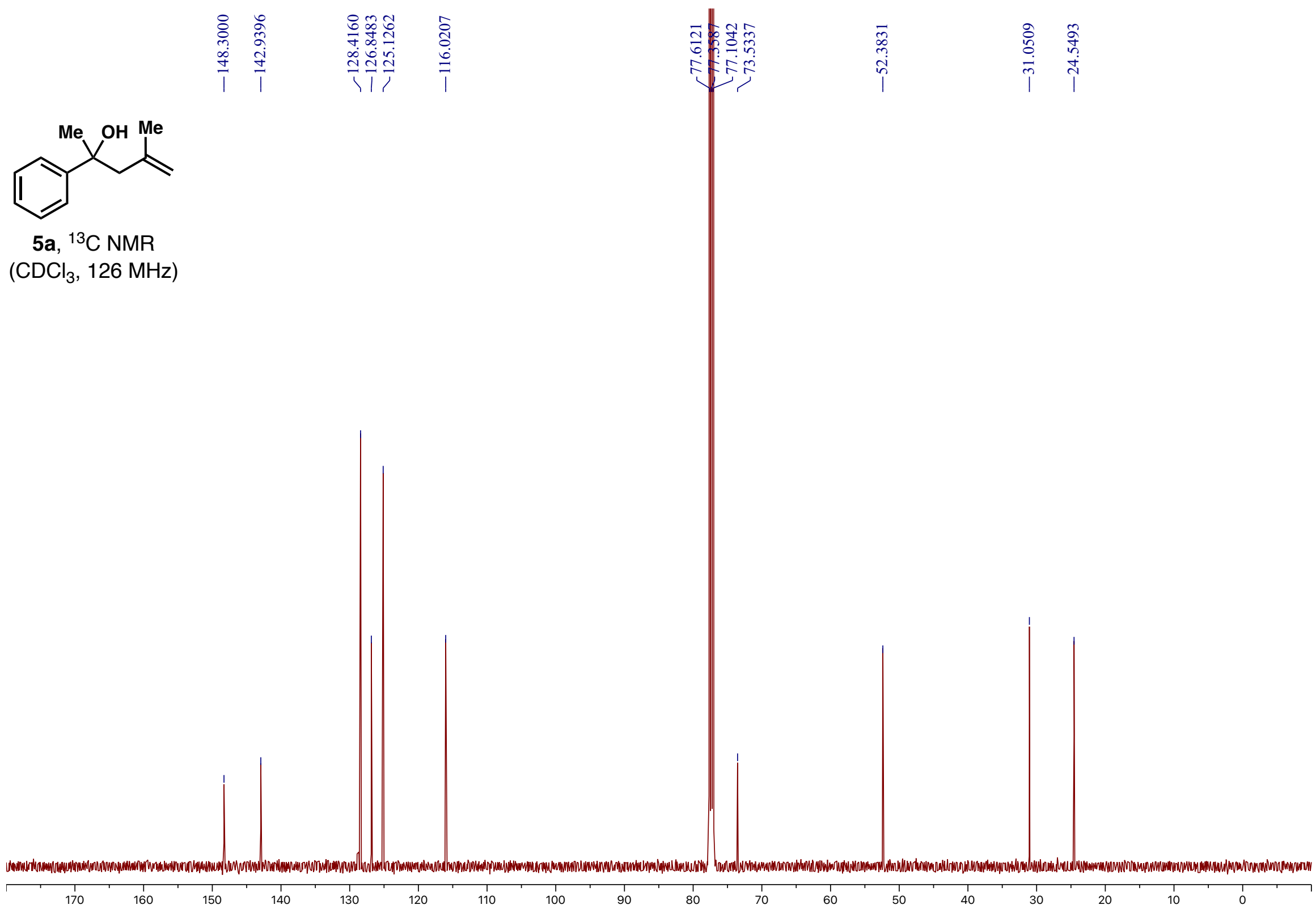

5a, ^{13}C NMR
($CDCl_3$, 126 MHz)

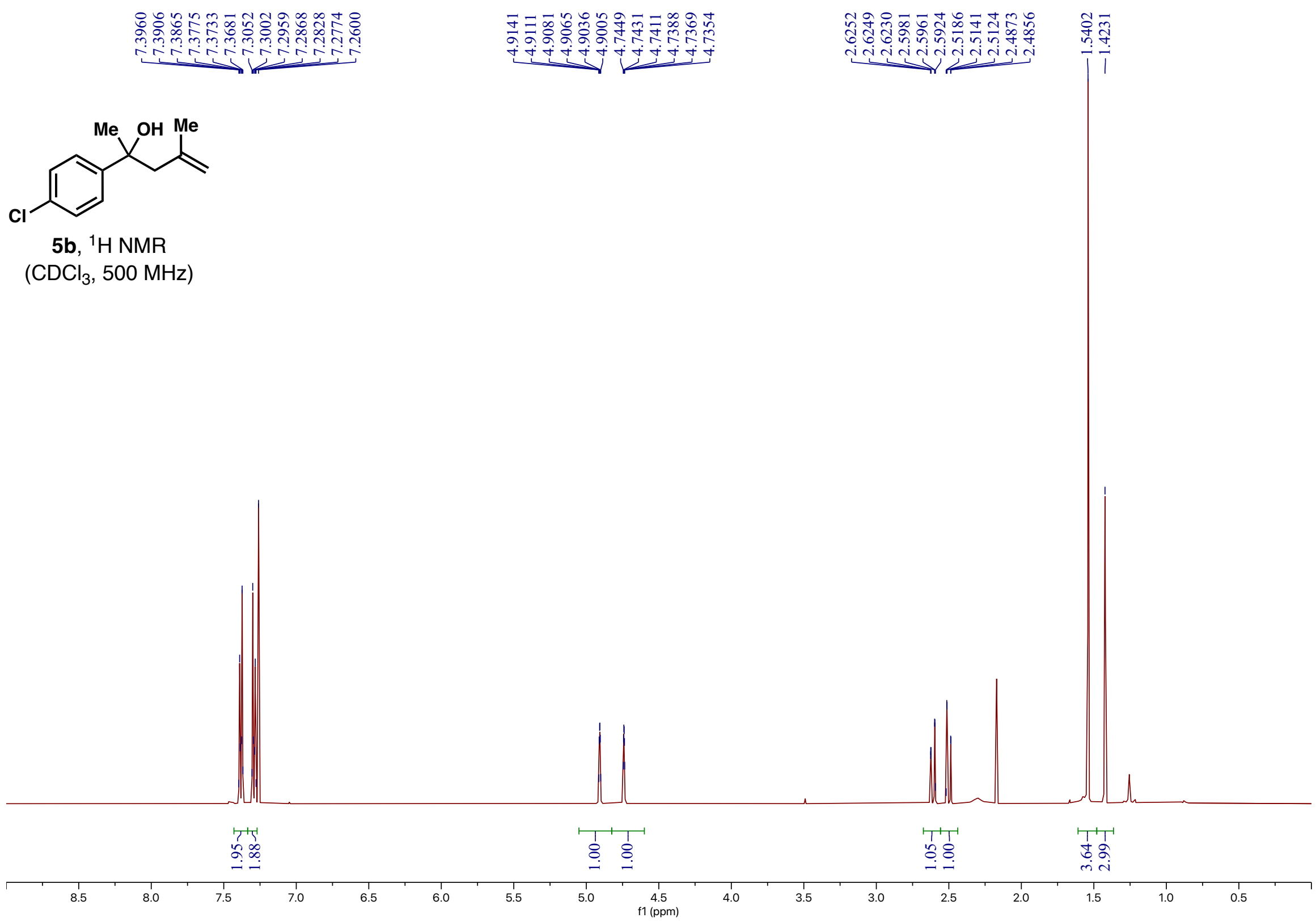

5b, ^{1}H NMR
($CDCl_3$, 500 MHz)

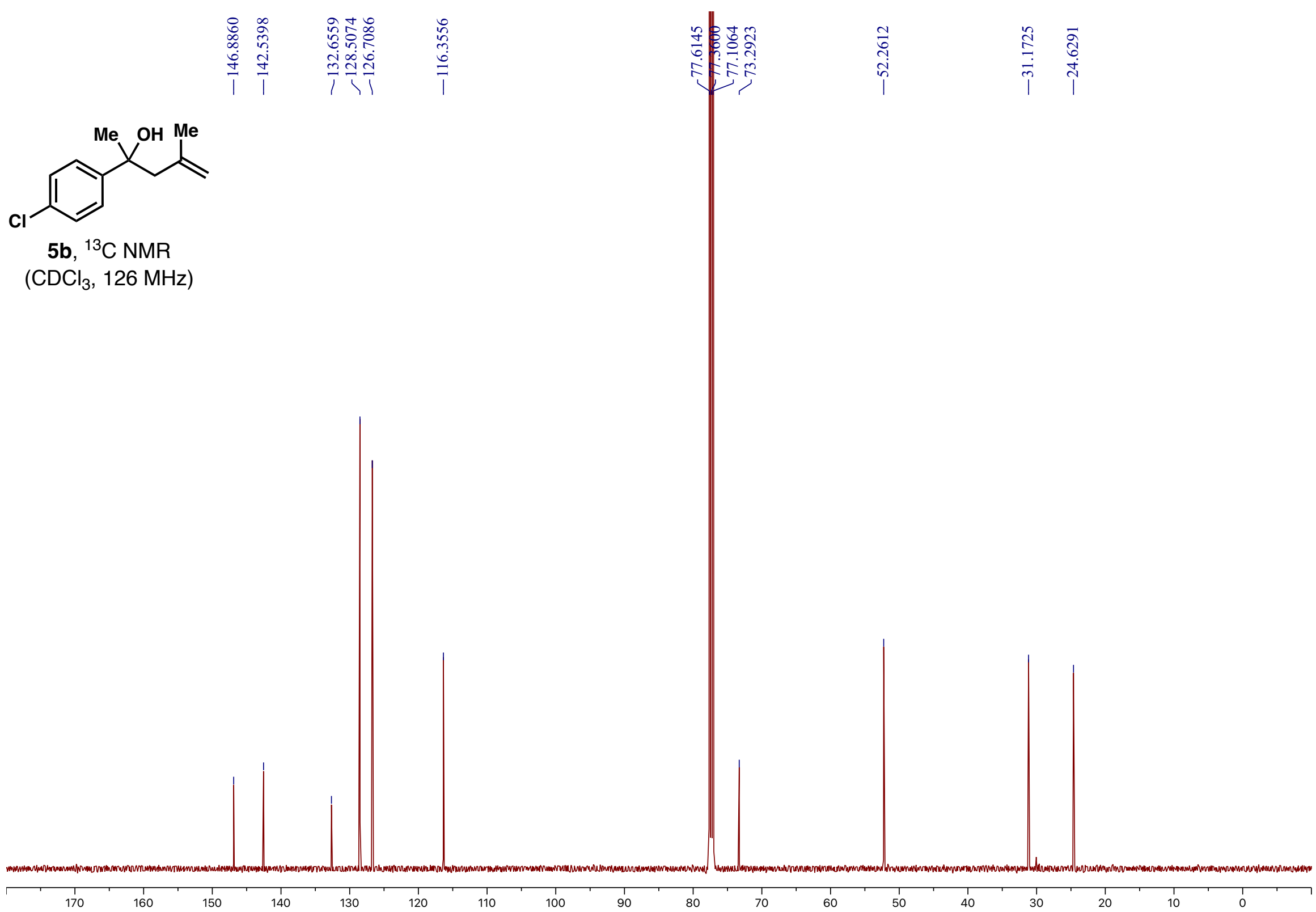

5b, ^{13}C NMR
($CDCl_3$, 126 MHz)

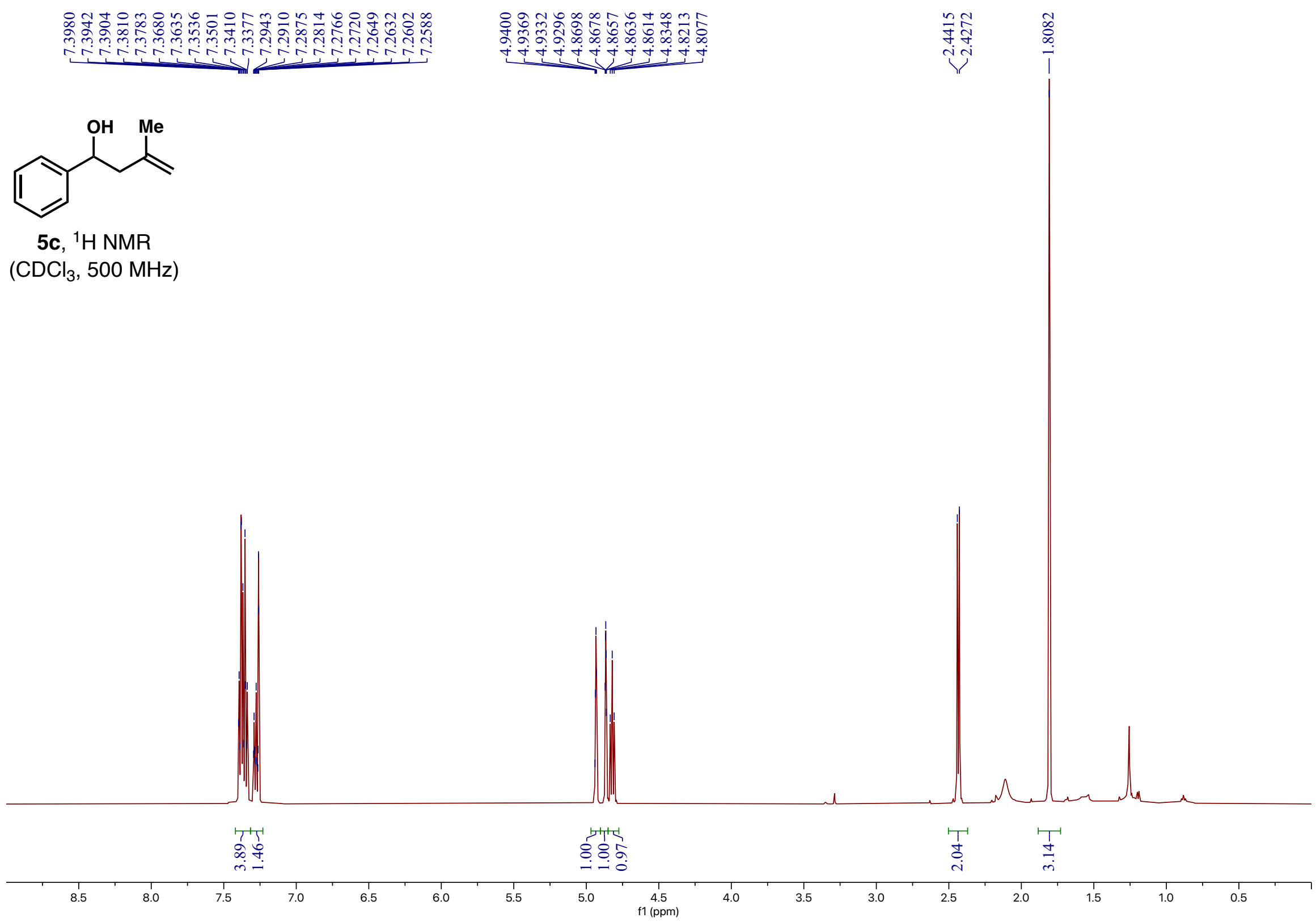
7.3980 7.3942 7.3904 7.3810 7.3783 7.3680 7.3635 7.3536 7.3501 7.3410 7.3377 7.2943 7.2910 7.2875 7.2814 7.2766 7.2720 7.2649 7.2632 7.2602 7.2588
4.9400 4.9369 4.9332 4.9296 4.8698 4.8678 4.8657 4.8636 4.8614 4.8348 4.8213 4.8077
2.4415 2.4272
1.8082
OH
Me
5c, ^{1}H NMR
($CDCl_3$, 500 MHz)
3.89 1.46 1.00 1.00 0.97 2.04 3.14
f1 (ppm)

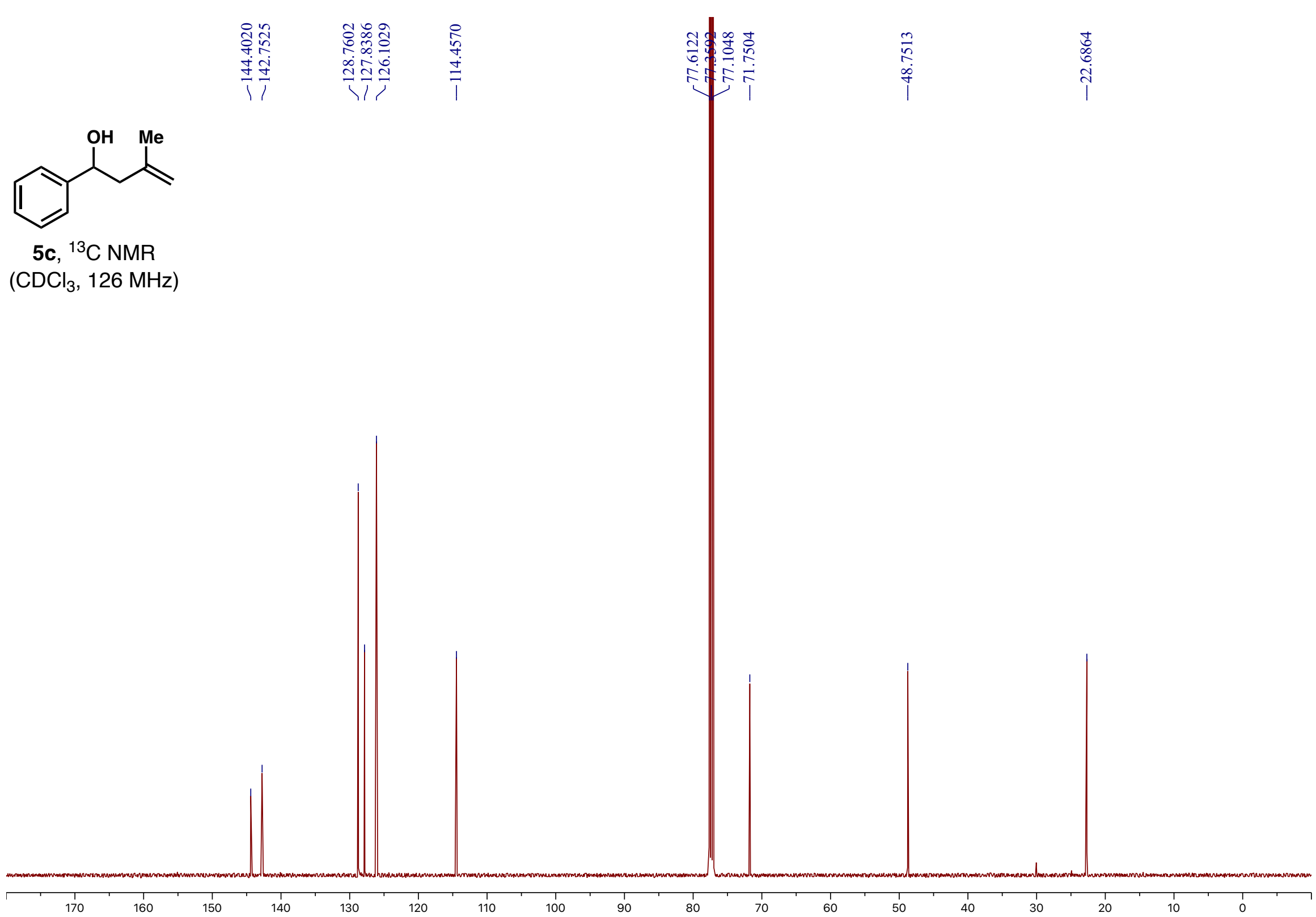
144.4020 142.7525
128.7602 127.8386 126.1029
114.4570
77.6122 77.3592 77.1048 71.7504
48.7513
22.6864
OH
Me
5c, ^{13}C NMR
($CDCl_3$, 126 MHz)
f1 (ppm)

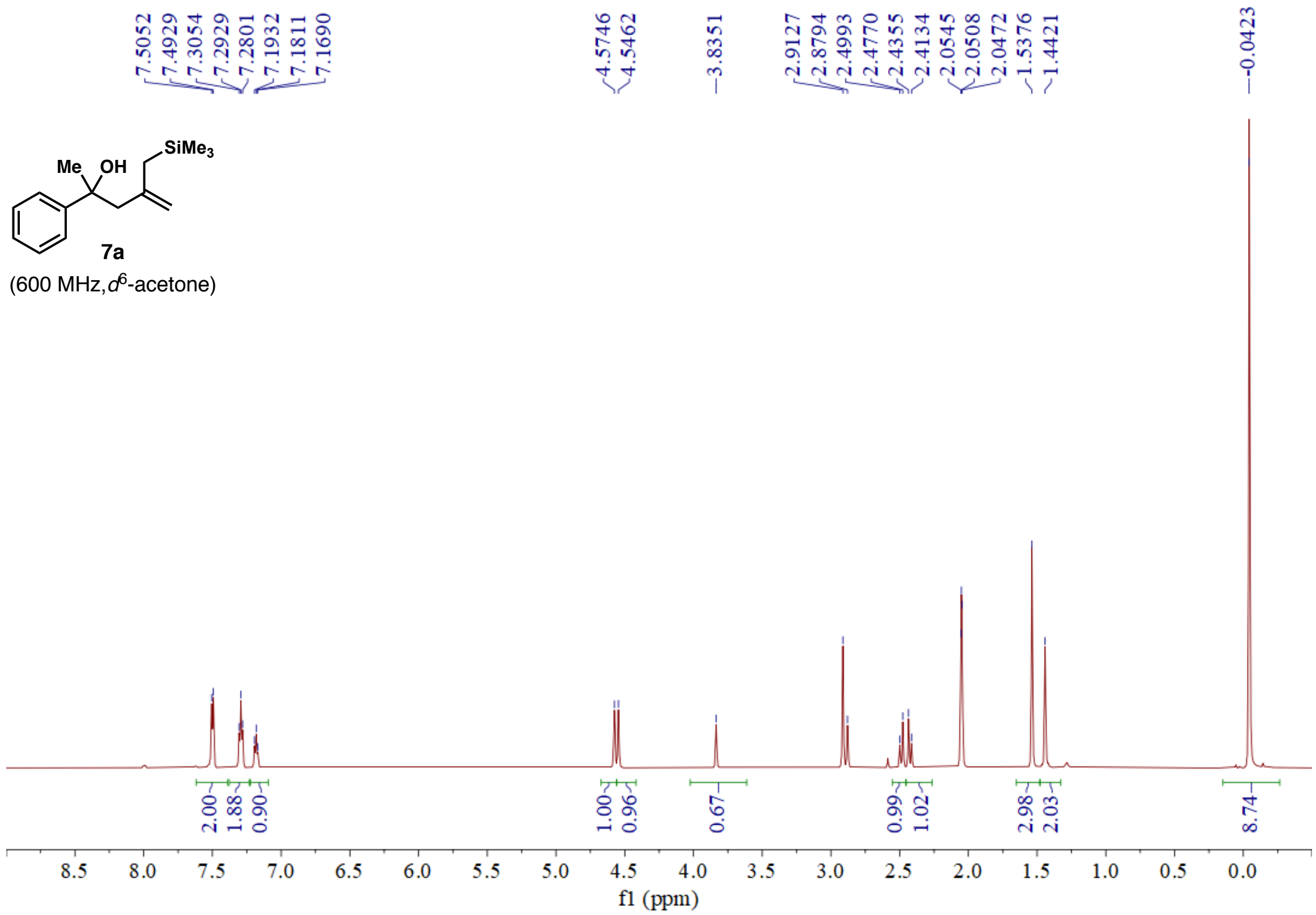
7.5052
7.4929
7.3054
7.2929
7.2801
7.1932
7.1811
7.1690
4.5746
4.5462
3.8351
2.9127
2.8794
2.4993
2.4770
2.4355
2.4134
2.0545
2.0508
2.0472
1.5376
1.4421
-0.0423
Me
OH
SiMe3
7a
(600 MHz,d6-acetone)
2.00
1.88
0.90
1.00
0.96
0.67
0.99
1.02
2.98
2.03
8.74
f1 (ppm)

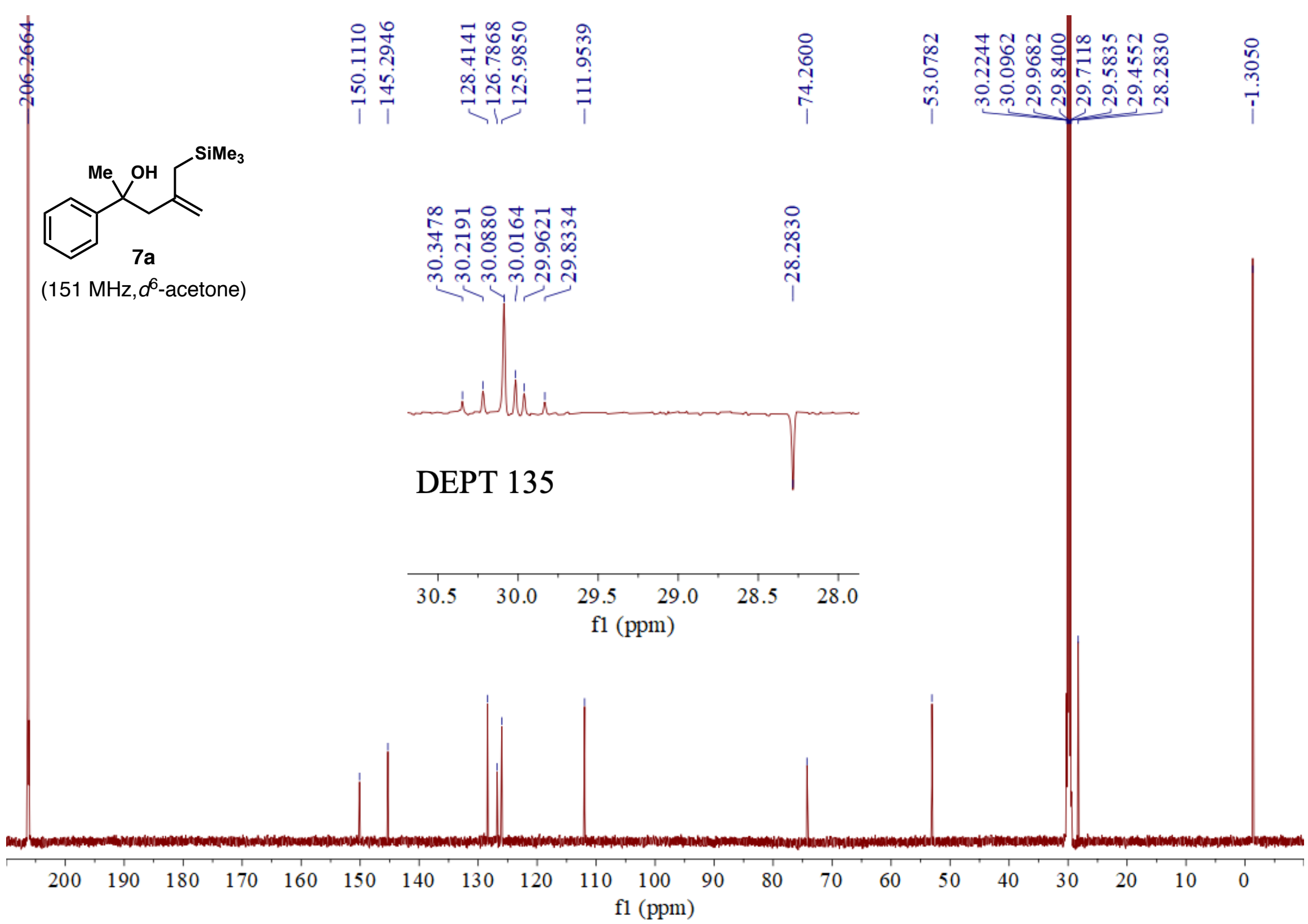
206.2664
150.1110
145.2946
128.4141
126.7868
125.9850
111.9539
74.2600
53.0782
30.2244
30.0962
29.9682
29.8400
29.7118
29.5835
29.4552
28.2830
-1.3050
Me
OH
SiMe3
7a
(151 MHz,d6-acetone)
30.3478
30.2191
30.0880
30.0164
29.9621
29.8334
28.2830
DEPT 135
f1 (ppm)

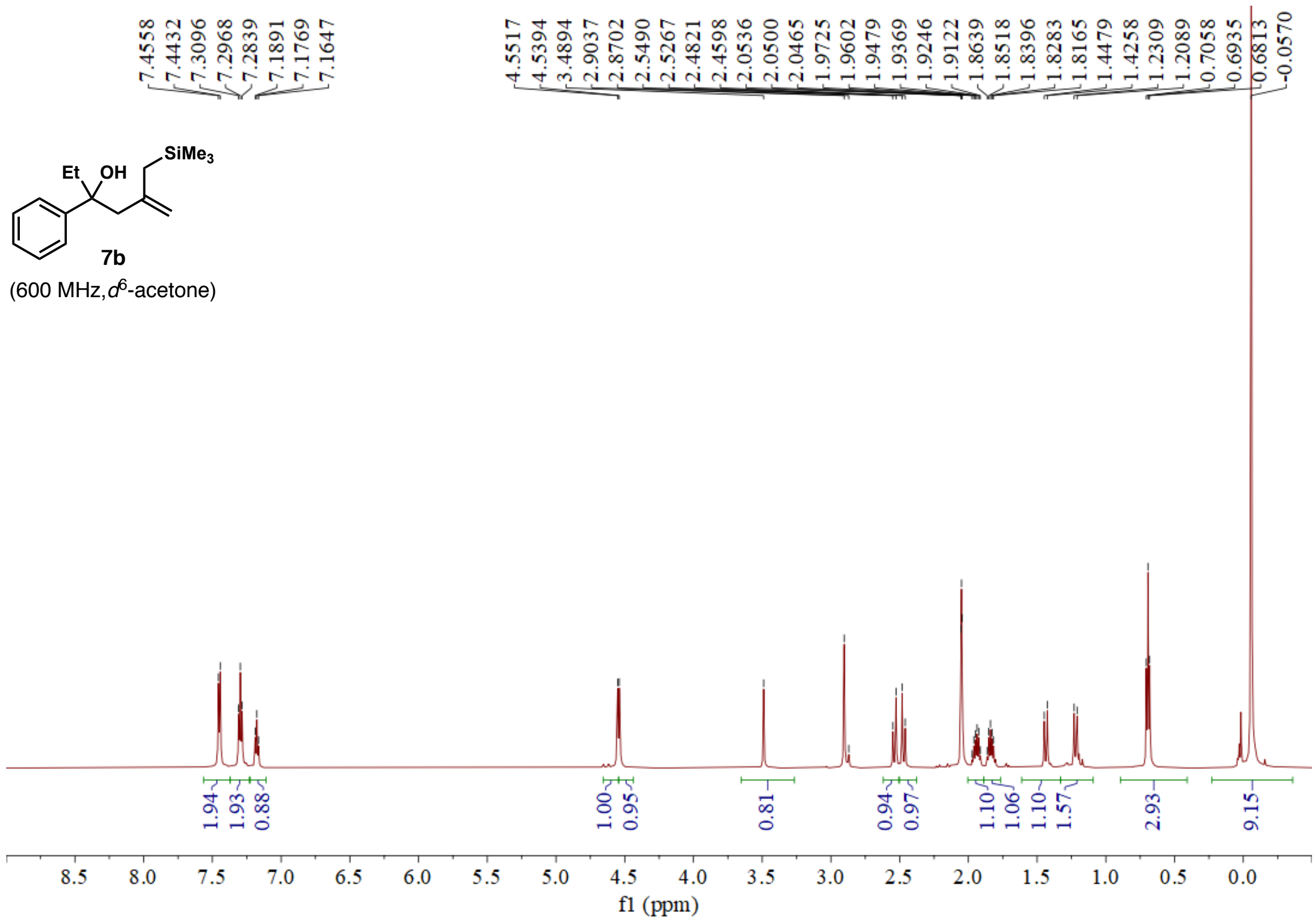
7.4558
7.4432
7.3096
7.2968
7.2839
7.1891
7.1769
7.1647
4.5517
4.5394
3.4894
2.9037
2.8702
2.5490
2.5267
2.4821
2.4598
2.0536
2.0500
2.0465
1.9725
1.9602
1.9479
1.9369
1.9246
1.9122
1.8639
1.8518
1.8396
1.8283
1.8165
1.4479
1.4258
1.2309
1.2089
0.7058
0.6935
0.6813
-0.0570
Et
OH
SiMe3
7b
(600 MHz, d6-acetone)
1.94
1.93
0.88
1.00
0.95
0.81
0.94
0.97
1.10
1.06
1.10
1.57
2.93
9.15
8.5 8.0 7.5 7.0 6.5 6.0 5.5 5.0 4.5 4.0 3.5 3.0 2.5 2.0 1.5 1.0 0.5 0.0
f1 (ppm)

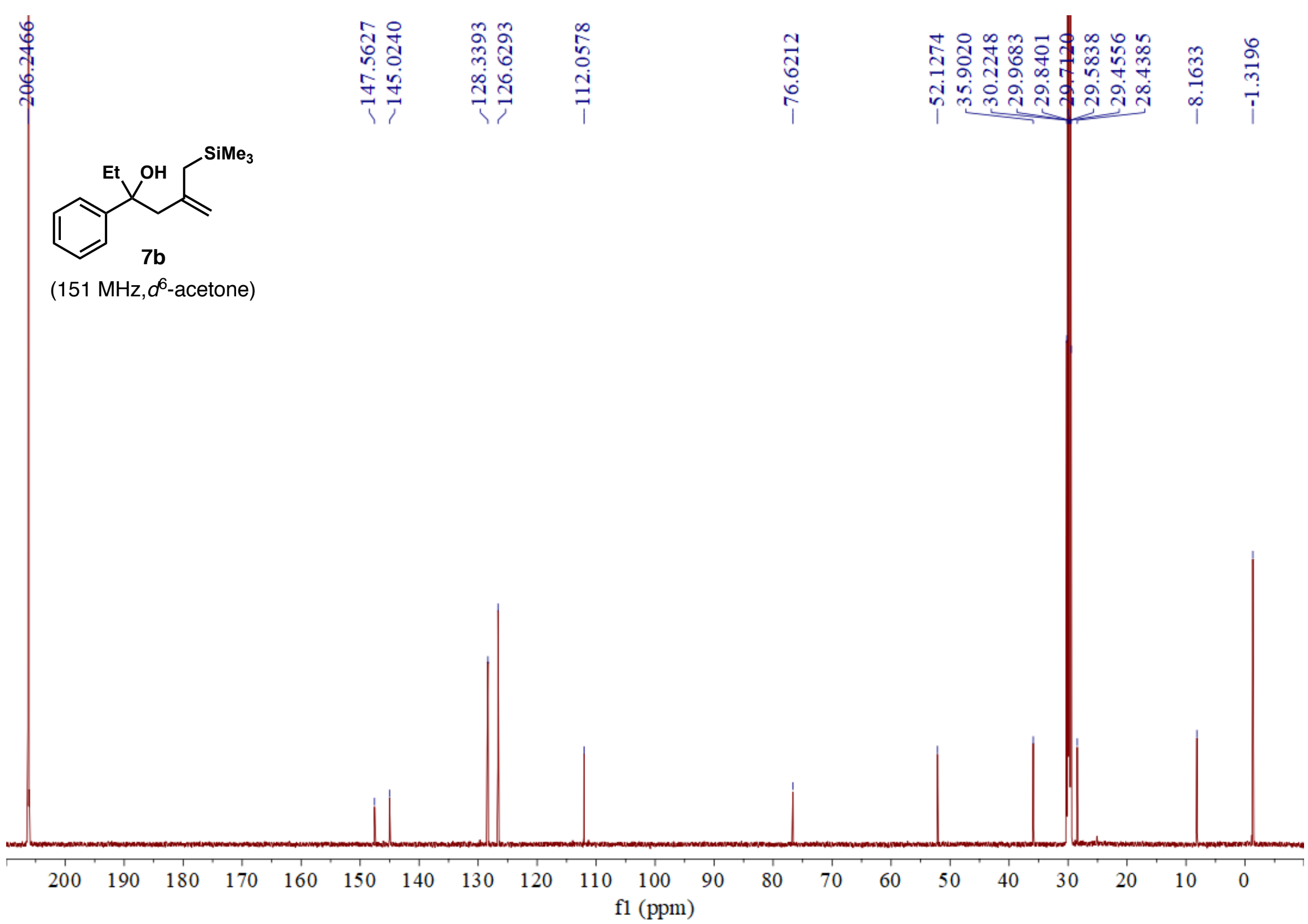
206.2466
147.5627
145.0240
128.3393
126.6293
112.0578
76.6212
52.1274
35.9020
30.2248
29.9683
29.8401
29.7120
29.5838
29.4556
28.4385
8.1633
-1.3196
Et
OH
SiMe3
7b
(151 MHz, d6-acetone)
200 190 180 170 160 150 140 130 120 110 100 90 80 70 60 50 40 30 20 10 0
f1 (ppm)

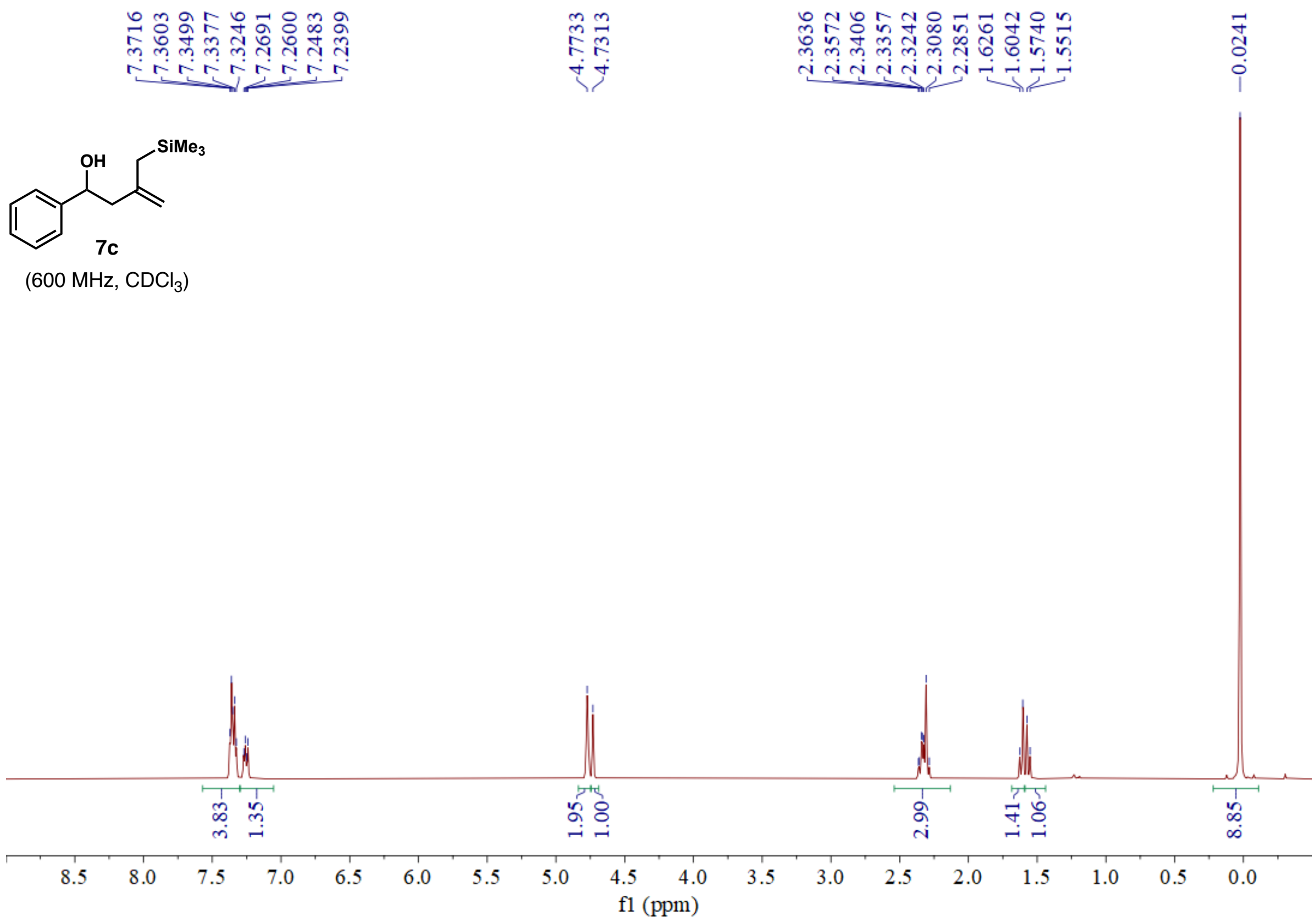
7.3716
7.3603
7.3499
7.3377
7.3246
7.2691
7.2600
7.2483
7.2399
4.7733
4.7313
2.3636
2.3572
2.3406
2.3357
2.3242
2.3080
2.2851
1.6261
1.6042
1.5740
1.5515
-0.0241
OH
SiMe3
7c
(600 MHz, CDCl3)
3.83
1.35
1.95
1.00
2.99
1.41
1.06
8.85
f1 (ppm)

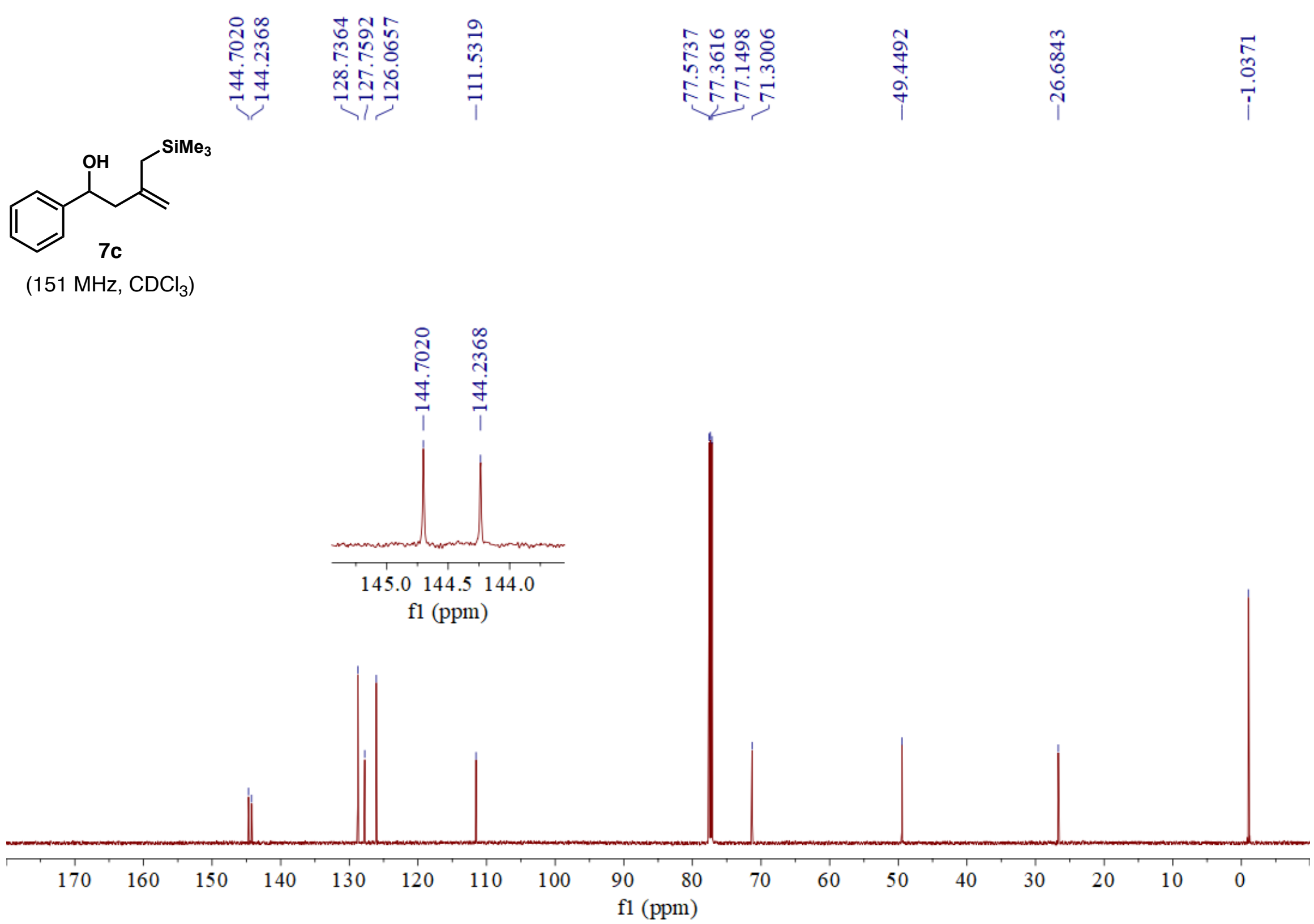
144.7020
144.2368
128.7364
127.7592
126.0657
111.5319
77.5737
77.3616
77.1498
71.3006
49.4492
26.6843
-1.0371
OH
SiMe3
7c
(151 MHz, CDCl3)
144.7020
144.2368
f1 (ppm)
f1 (ppm)

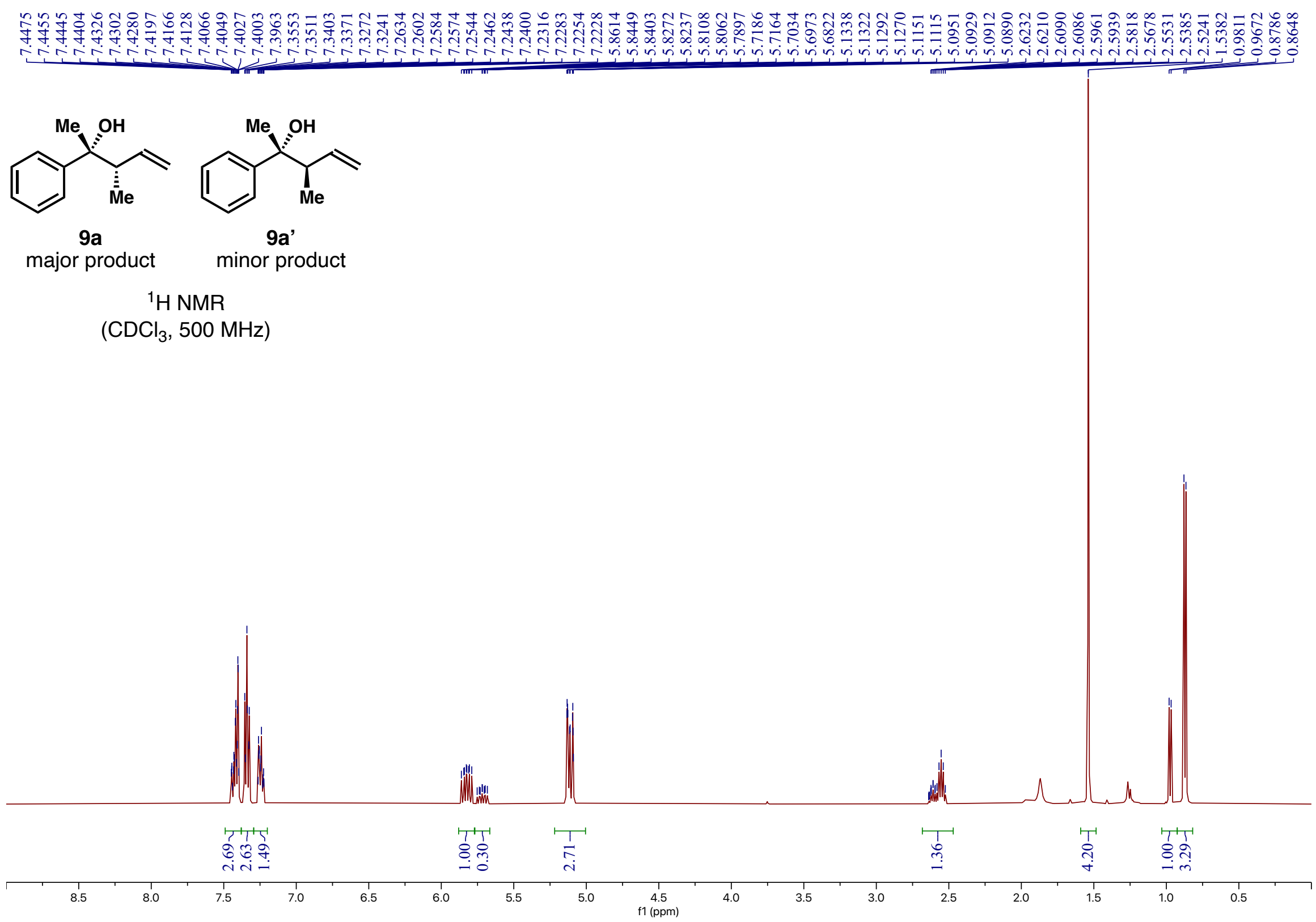
7.4475
7.4455
7.4445
7.4404
7.4326
7.4302
7.4280
7.4197
7.4166
7.4128
7.4066
7.4049
7.4027
7.4003
7.3963
7.3553
7.3511
7.3403
7.3371
7.3272
7.3241
7.2634
7.2602
7.2584
7.2574
7.2544
7.2462
7.2438
7.2400
7.2316
7.2283
7.2254
7.2228
5.8614
5.8449
5.8403
5.8272
5.8237
5.8108
5.8062
5.7897
5.7186
5.7164
5.7034
5.6973
5.6822
5.1338
5.1322
5.1292
5.1270
5.1151
5.1115
5.0951
5.0929
5.0912
5.0890
2.6232
2.6210
2.6090
2.6086
2.5961
2.5939
2.5818
2.5678
2.5531
2.5385
2.5241
1.5382
0.9811
0.9672
0.8786
0.8648
Me
OH
Me
9a
major product
Me
OH
Me
9a'
minor product
1H NMR
(CDCl3, 500 MHz)
2.69
2.63
1.49
1.00
0.30
2.71
1.36
4.20
1.00
3.29
8.5
8.0
7.5
7.0
6.5
6.0
5.5
5.0
4.5
4.0
3.5
3.0
2.5
2.0
1.5
1.0
0.5
f1 (ppm)

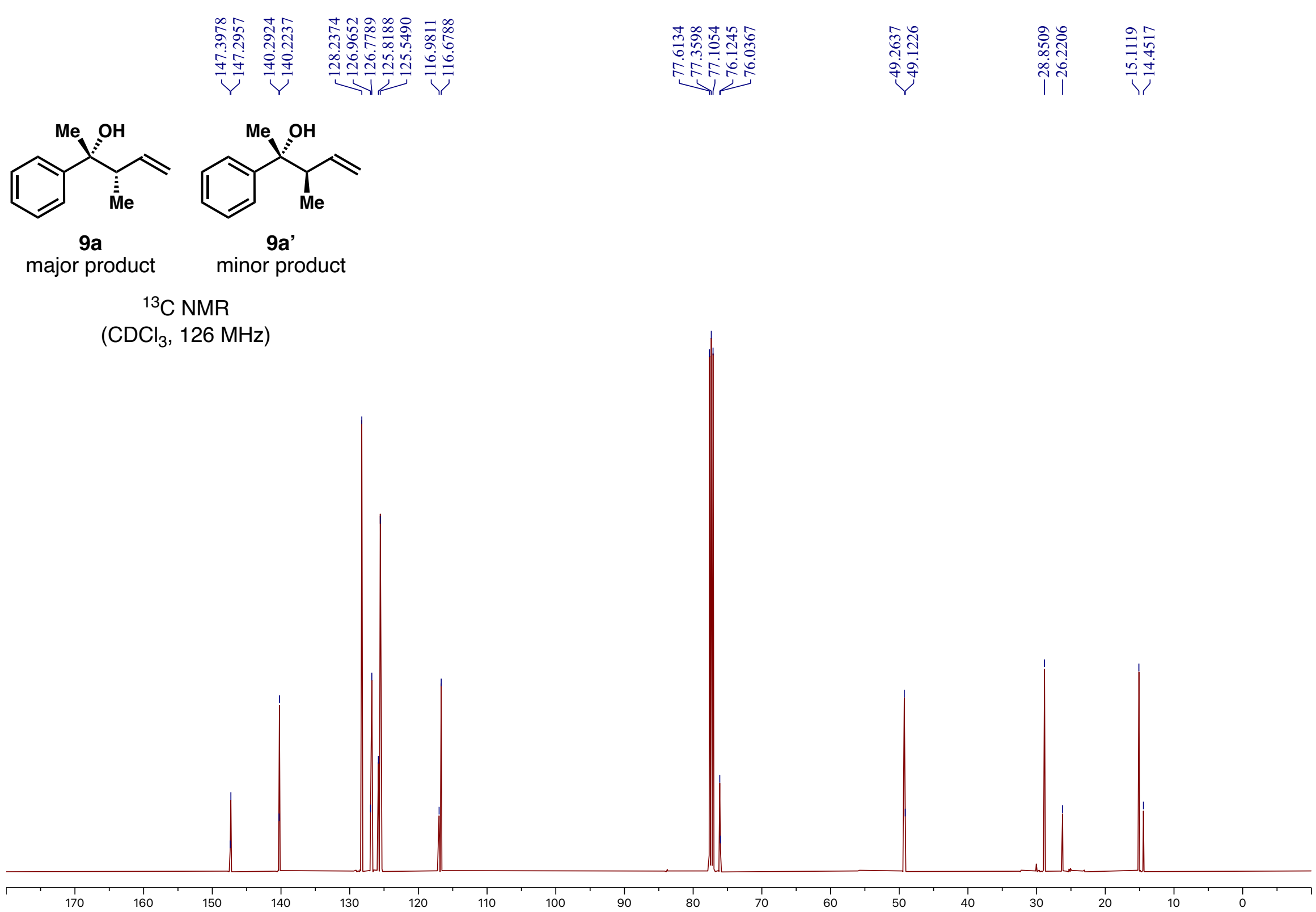
147.3978
147.2957
140.2924
140.2237
128.2374
126.9652
126.7789
125.8188
125.5490
116.9811
116.6788
77.6134
77.3598
77.1054
76.1245
76.0367
49.2637
49.1226
28.8509
26.2206
15.1119
14.4517
Me
OH
Me
9a
major product
Me
OH
Me
9a'
minor product
13C NMR
(CDCl3, 126 MHz)
170
160
150
140
130
120
110
100
90
80
70
60
50
40
30
20
10
0
f1 (ppm)

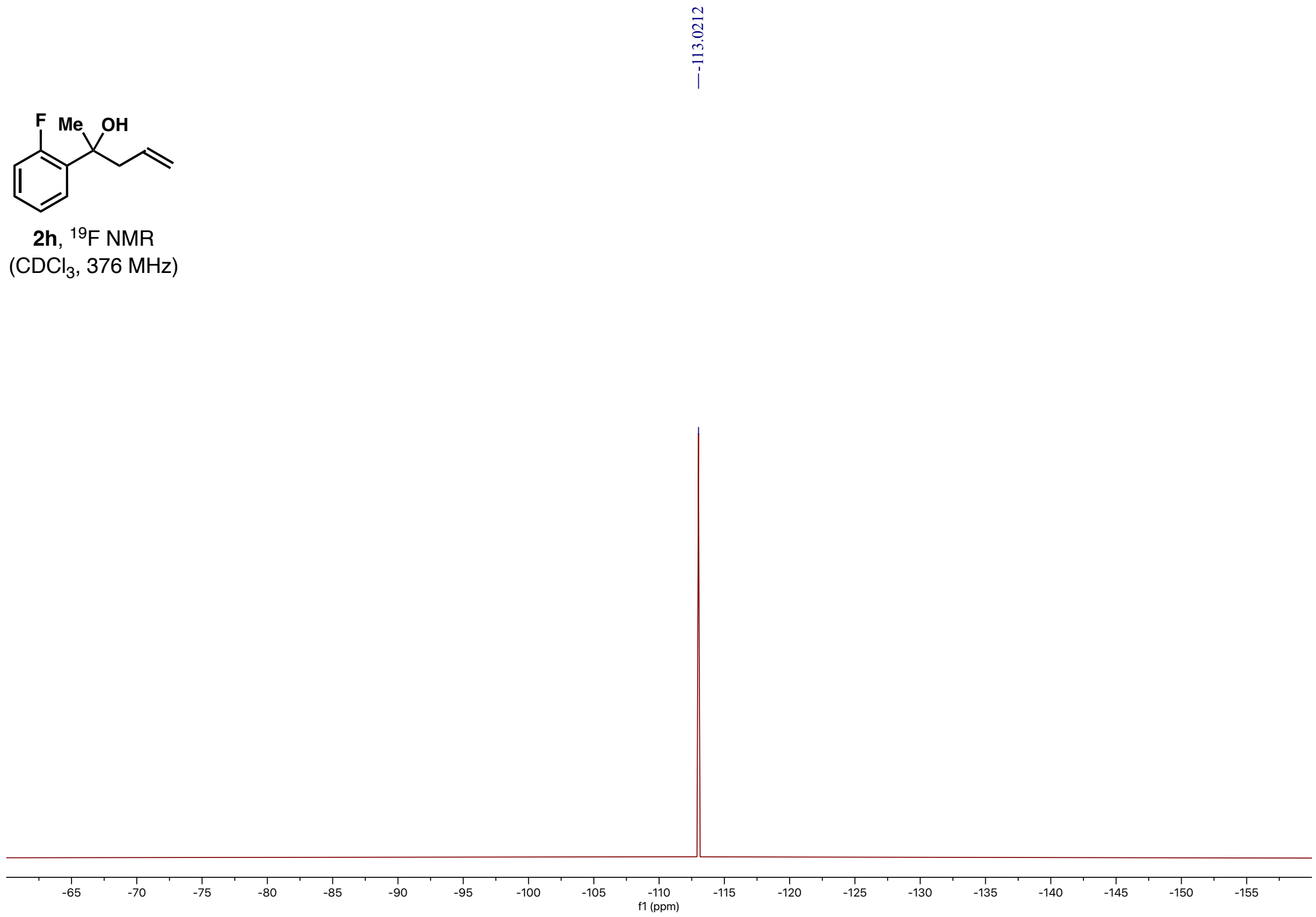

2h, ^{19}F NMR
($CDCl_3$, 376 MHz)
